# Development of Visible-Light Driven Cu(I) Complex Photosensitizers for Photocatalytic CO_2_ Reduction

**DOI:** 10.3389/fchem.2019.00418

**Published:** 2019-06-06

**Authors:** Hiroyuki Takeda, Yu Monma, Haruki Sugiyama, Hidehiro Uekusa, Osamu Ishitani

**Affiliations:** Department of Chemistry, School of Science, Tokyo Institute of Technology, Tokyo, Japan

**Keywords:** Cu(I) diimine complex, CO_2_ reduction photocatalyst, redox photosensitizer, visible-light absorption, emission

## Abstract

The visible-light responsive Cu(I)-complex photosensitizers were developed by introducing various aromatic substituents at the 4,7-positions of a 2,9-dimethyl-1,10-phenanthroline (dmp) ligand in a heteroleptic Cu^I^(dmp)(DPEphos)^+^-type complexes (DPEphos = [2-(diphenylphosphino)phenyl]ether) for photocatalytic CO_2_ reduction. Introducing biphenyl groups (Bp-) on the dmp ligand enhanced the molar extinction coefficient (ε) of the metal-to-ligand charge transfer (MLCT) band in the visible region (ε = 7,500 M^−1^cm^−1^) compared to that of the phenyl (Ph-)-containing analog (ε = 5,700 M^−1^cm^−1^ at λ_max_ = 388 nm). However, introducing 4-R-Ph- groups (R = the electron-withdrawing groups NC-, or NO_2_-) led to a red shift in the band to λ_max_ = 390, 400, and 401 nm, respectively. Single-crystal X-ray analysis showed the Ph- groups were twisted because of the steric repulsion between the 2,6-protons of the Ph- groups and 5,6-protons of the dmp ligand. The result strongly indicated that the π-conjugation effect of the 4-R-Ph- groups is so weak that the lowering of the energy of the dmp π^*^ orbitals is small. However, when 4-R-ph- was substituted by a 5-membered heterorings, there was a larger red shift, leading to an increase in the ε value of the MLCT absorption band. Thus, the substitution to 2-benzofuranyl- groups resulted in visible-light absorption up to 500 nm and a shoulder peak at around 420 nm (ε = 12,300 M^−1^cm^−1^) due to the expansion of π-conjugation over the substituted dmp ligand. The photocatalytic reaction for CO_2_ reduction was tested using the obtained Cu^I^ complexes as photosensitizers in the presence of a Fe(dmp)_2_(NCS)_2_ catalyst and 1,3-dimethyl-2-phenyl-2,3-dihydro-1*H*-benzo[d]imidazole as a sacrificial reductant, which showed improved CO generation.

## Introduction

As one of the most important components of artificial photosynthesis, photocatalytic CO_2_ reduction is attracting much attention. Although metal complexes are key players in CO_2_ reduction due to their promising photosensitizing and/or catalytic ability, the metals used are often limited to low abundance ones, such as Ru, Re, Os, or Ir (Yamazaki et al., 2015). Thus, substituting these metals with more abundant elements is now being widely focused on in many groups, using the first row transition metals such as Mn, Fe, Co, and Ni to produce a multi-electron catalyst for CO_2_ reduction (Takeda et al., 2017). However, such the attempts, especially to produce a redox photosensitizer (which functions as a light absorber to transfer an excited electron in its excited state to the catalyst), are limited; the excited state deactivates quickly because of low lying d-d excited state.

Thus, the heteroleptic Cu^I^ phenanthroline complexes, such as [Cu^I^(dmp)(P)_2_]^+^ (dmp = 2,9-dimethyl-1,10-phenanthroline, P = phosphine ligand), are gaining popularity, because of their long lifetimes, showing strong metal-to-ligand (MLCT) excited-state emission even in a solution at room temperature due to the Cu^I^ center's *d*^10^ configuration (Ruthkosky et al., 1998). Although homoleptic-type [Cu^I^(dmp)_2_]^+^ has the advantage as a photosensitizer in terms of visible-light utilization because it can absorb longer wavelength light (Khnayzer et al., 2013), in the excited state, its oxidation power is less and lifetime shorter than those of a heteroleptic complex. Thus, heteroleptic-type of Cu complexes are now being used as redox photosensitizers to construct many photocatalytic systems (Lazorski and Castellano, 2014). Since these Cu complexes had previously been reported to have excellent emissive properties by McMillin et al. (Breddels et al., 1982), recent developments by Beller et al. on the catalytic H_2_ evolution reaction using such complexes (Luo et al., 2013; Mejía et al., 2013; Rosas-Hernández et al., 2017) have opened up the way in this area.

However, when using heteroleptic Cu complexes as redox photosensitizers in photocatalytic reactions, some issues remain, such as their stability and light-absorption in the visible region. The first issue, stability, has previously been tackled by our group, where the stability of the Cu^I^ complex photosensitizer was improved by connecting phenanthroline phosphine ligands together using –C_4_H_8_– alkyl groups (Takeda et al., 2016). Because the tetrahedral coordination around the Cu^I^ center was maintained in this molecule, the complex has a long lifetime in the excited state, exhibiting strong emission. Thus, the resulting Cu^I^ dimer complexes were much more stable, not only against thermal ligand exchanges in the ground state (Kaeser et al., 2013; Lennox et al., 2016) but also against “exciplex” deactivation of the excited state, even when CH_3_CN was used as a coordinating solvent (McMillin et al., 1985) and a Cu^0^ metal particle formed via ligand dissociation in the one-electron reduced state (Eggleston et al., 1997). Thus, utilizing this Cu^I^ complex as a redox photosensitizer in the photocatalytic CO_2_ reduction, we could obtain the best photocatalytic performances for CO_2_ reduction and clarified the photosensitizing scheme, which forms the corresponding one-electron reduced species through reductive quenching by a reductant such as BIH (1,3-dimethyl-2-phenyl-2,3-dihydro-1*H*-benzo[d]imidazole) in the excited state, donating the added electrons to CO_2_ reduction catalyst (Takeda et al., 2018).

So far, many groups have attempted to red shift the ^1^MLCT absorption band by expanding the dmp ligand's π-conjugation. These attempts are reasonable because the lowest excited state of the Cu^I^ complexes can be mainly attributed to the MLCT state, in which a charge on the Cu^I^ center transfers to the phenanthroline ligand's low-lying π^*^ orbitals. Tsubomura et al. introduced phenyl groups at the 4,7-positions of dmp, which is known *neocuproine*, to produce a ligand called *bathocuproine* (bcp), enhancing the molar extinction coefficient of the resulting Cu^I^ complex, thus causing a red shift in the absorption maximum of the MLCT band (Tsubomura et al., 2015). The Cu^I^ complexes having a pyrazine- or phenazine-fused dmp ligand have been also reported. However, the resulting Cu complexes showed absorption maxima at around 380 nm (Xu et al., 2015; Heberle et al., 2017). The same group also reported introducing (thiophen-2-yl)vinyl groups at the 2,9-positions of the phenanthroline, but the MLCT absorption band maximum of the Cu complex was still only observed at 388 nm (Chen et al., 2017). Although introducing 4-benzoic acid or thiophene-3-carboxylic acid at the 4,7-positions shifts the absorption band to a longer wavelength, the absorption maxima are limited to 394 and 392 nm, respectively (Chen et al., 2017). Introducing sulfonate groups at the 5,6-positions of bcp also limits the absorption maximum to 390 nm (Rockstroh et al., 2014). Recently, Giereth et al. reported a Cu complex having anthracene-fused dmp, which shows absorption at a longer wavelength at around 430 nm (Giereth et al., 2019). McCullough et al. also reported a unique Cu complex incorporating 2,2′-biquinoline as an α-diimine and applied it as a photocatalytic system for H_2_ evolution (McCullough et al., 2018). This type of complex showed a surprising red shift in its maximum to 455 nm (Zhang et al., 2007). However, the first-reduction potential of these Cu complexes underwent a positive shift from −2 V for the dmp complex to −1.19 V vs. Fc^+^/Fc in CH_3_CN for the anthracene-fused dmp complex and −1.69 V vs. Fc^+^/Fc in CH_2_Cl_2_ for the biquinoline complex, that are more positive than that of the common redox-photosensitizer ${\text{Ru}(\text{dmb})}_{3}^{2+}$ (dmb = 4,4′-dimethyl-2,2′-bipyridine). Thus, further study of these types of Cu^I^ complexes is required so that they can be used as visible-light absorbing redox-photosensitizers.

In this study, Cu^I^ complexes are synthesized with new bcp-based phenanthroline ligands, with electron-withdrawing groups on the phenyl groups, or various different aromatic groups such as heterorings, instead of the phenyl groups of the bcp ligand. The aim is improving these systems' visible-light absorption. The Cu^I^ complexes' UV-Vis absorption spectra, photophysical properties, and electrochemical properties were examined, providing evidence for a significant red shift in the MLCT absorption band. The redox-photosensitizing ability for photocatalytic CO_2_ reduction of these Cu^I^ complexes was also tested using Fe(dmp)_2_(NCS)_2_ as a catalyst in the presence of a reductant.

## Results and Discussion

**Figure 1:**
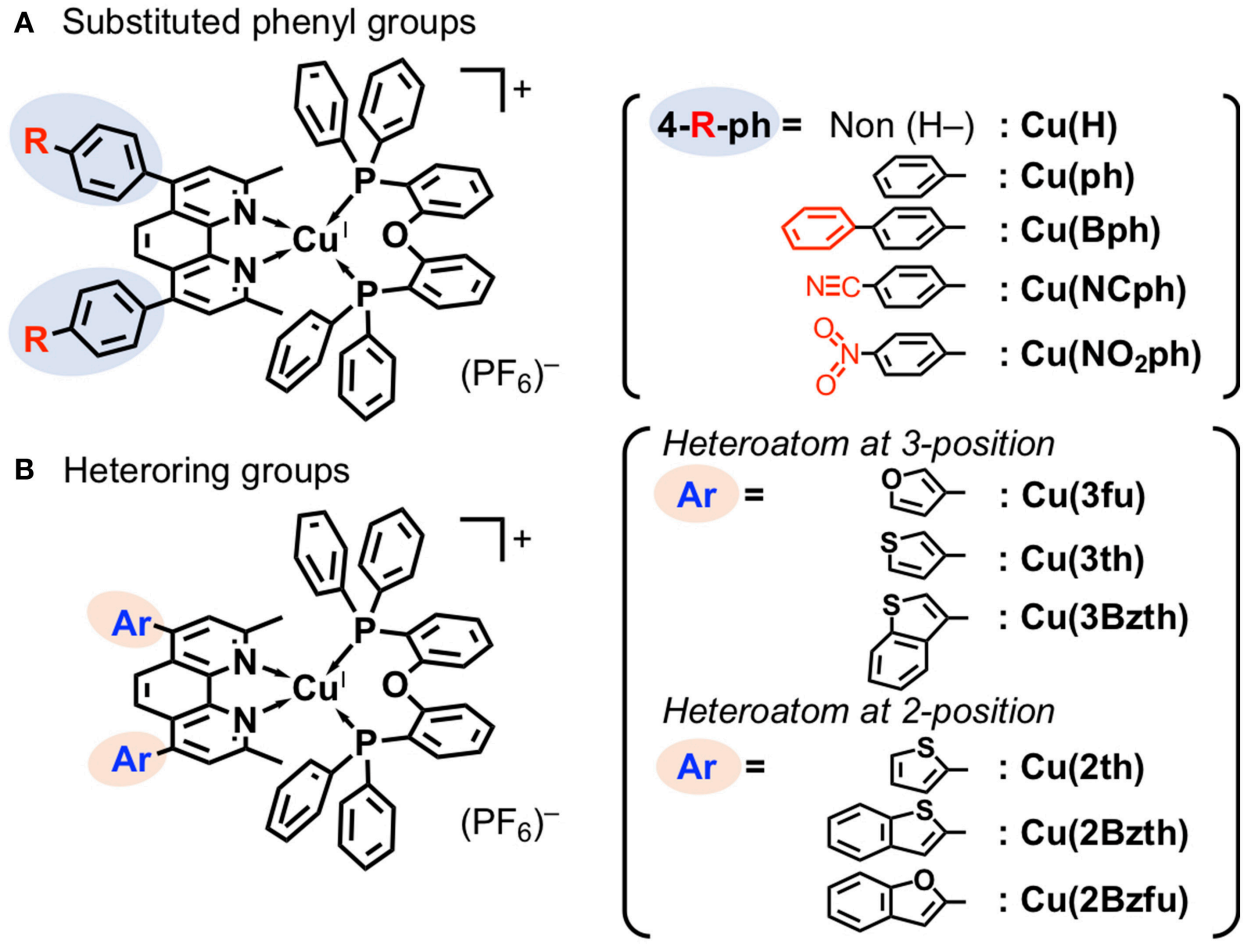
Cu^I^ complexes with an aryl-substituted dmp [aryl = substituted phenyl **(A)** and heteroring **(B)** groups] and a DPEphos ligands.

The structures of newly synthesized Cu^I^ complexes' structures are shown in Figure 1 alongside the reported Cu^I^ complexes **Cu(H)** (Cuttell et al., 2002) and **Cu(ph)** (Luo et al., 2013). Two types of substituents were introduced at the 4,7-positions of the dmp ligand, (a) 4-substituted phenyl groups (Figure 1A) and (b) heteroring aryl groups (Figure 1B). In the type (a) substituents, **Cu(Bph)** is an expanded form of **Cu(ph)** with an increased number of the phenyl groups, while **Cu(NCph)** and **Cu(NO**_**2**_**ph)** have π-electron withdrawing cyano and nitro substituents on the phenyl groups, respectively. In the (b) substituents, the ligands have aryl groups that are smaller in size, i.e., 5-membered heterorings containing O or S atoms at 2- or 3-position, than the phenyl groups adjacent to the dmp ligand. Because S and O atoms have different electronegativities of 3.44 and 2.58, respectively, and van der Waals radii (Bondi, 1964) of 1.52 and 1.80 Å, respectively, the electronic properties of the Cu^I^ complexes should be different.

**Figure 2:**
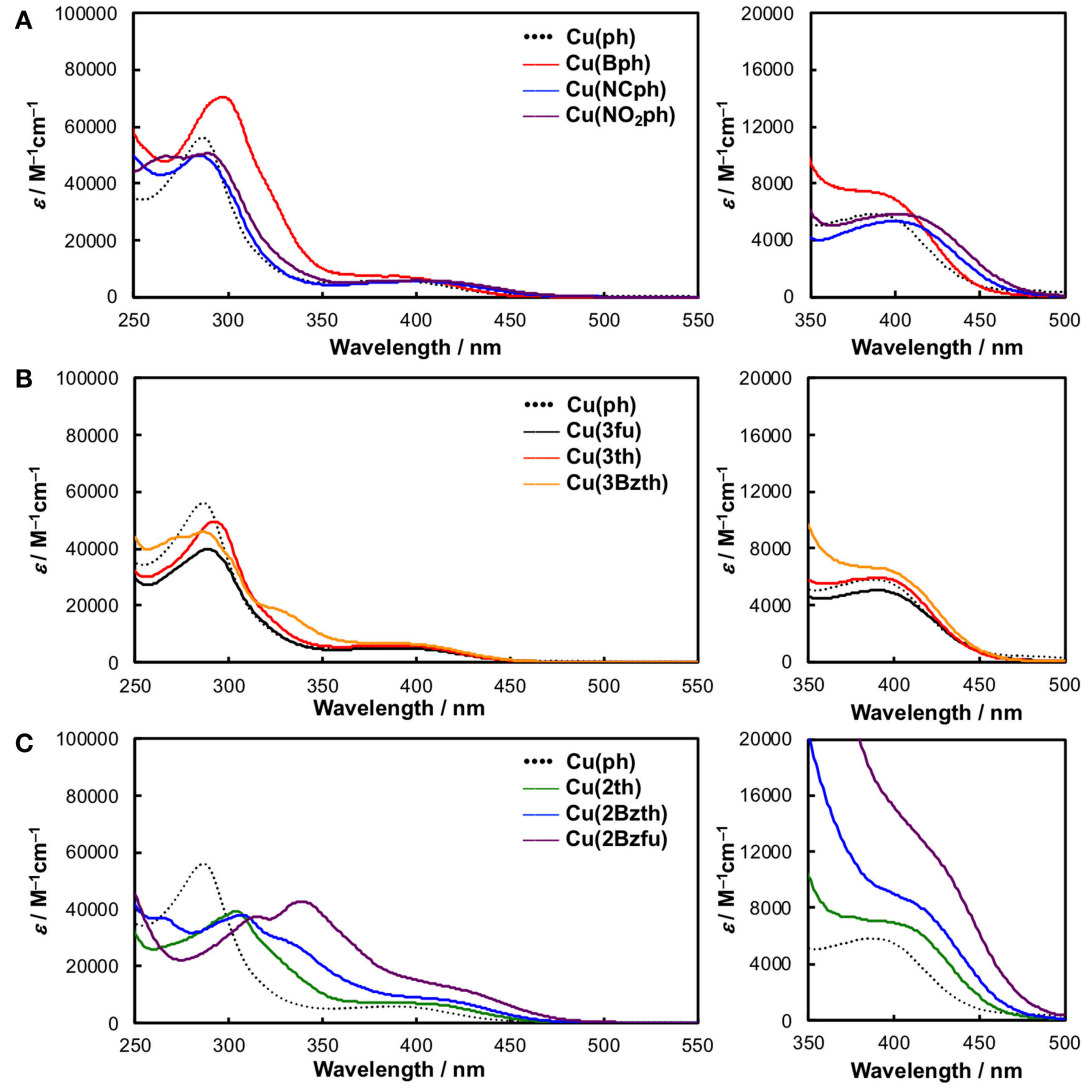
UV-Vis absorption spectra of the Cu^I^ complexes in CH_2_Cl_2_ alongside the spectrum of **Cu(ph)** (dotted black line) as a reference. **(A) Cu(ph)** (dotted black line), **Cu(Bph)** (red), **Cu(NCph)** (blue), and **Cu(NO**_**2**_**ph)** (purple). **(B) Cu(3fu)** (solid black line), **Cu(3th)** (red), and **Cu(3Bzth)** (yellow). **(C) Cu(2th)** (green), **Cu(2Bzth)** (blue), and **Cu(2Bzfu)** (purple). Right panels on each spectrum show magnified section of the spectra, zoomed in on the ^1^MLCT absorption.

The Cu^I^ complexes' UV-Vis absorption spectra in a non-coordinating solvent, such as CH_2_Cl_2_, are shown in Figure 2, and the data are summarized in Table 1. As a representative system, **Cu(ph)** exhibited a ^1^MLCT transition band at 350–500 nm that was moderate in intensity (molar extinction coefficient (ε) ~6000 M^−1^s^−1^) and an intraligand π-π^*^ transition band in the shorter-wavelength range with stronger intensity (ε ~50000 M^−1^s^−1^), as shown in Figures 2A–C (dotted line). Because the ^1^MLCT transition is the lowest in energy from the spectrum, the lowest excited state of this complex is strongly indicated to be the MLCT state. Although the π-π^*^ excited state which is higher in energy can proceed thermal relaxation to populate the lowest state, direct excitation of the lowest MLCT transition in the visible region can be assumed to be the most significant in a redox-photosensitizing reaction. Thus, we focused ^1^MLCT band of the Cu^I^ complexes, referring the π-π^*^ band as an indicator of π-system expansion over the aryl-substituted diimine ligands.

**Table 1:** Photophysical Properties of the Cu^I^ Complexes in CH_2_Cl_2._.

Complex	λ_abs_^a^/nm (ε^b^/M^−1^cm^−1^)	λ_em_^c^/nm	Φ_em_^d^	τ_em_^e^/μs
**Cu(H)**	380 (2,700)	562	0.43	15.5
**Cu(ph)**	388 (5,700)	575	0.52	19.4
**Cu(Bph)**	388sh (7,500)	577	0.60	22.5
**Cu(NCph)**	400 (5,300)	609	0.16	5.6
**Cu(NO**_**2**_**ph)**	401 (5,800)	640	0.00	0.01
**Cu(3fu)**	390 (5,000)	580	0.24	0.06, 0.69, 8.3, 23.6
**Cu(3th)**	391 (5,900)	579	0.35	22.6
**Cu(3Bzth)**	395sh (6,600)	580	0.45	0.03, 16.5
**Cu(2th)**	400 (6,800)	601	0.20	0.02, 93.3
**Cu(2Bzth)**	410sh (8,200)	614	0.06	0.02, 0.5, ~130^f^
**Cu(2Bzfu)**	420sh (12,300)	611, 650sh	0.07	0.02, ~240^f^

a *λ_abs_, ^1^MLCT absorption maxima*;

b *ε, molar extinction coefficients*;

c *λ_em_, emission maxima*;

d *Φ_em_, emission quantum yield*;

e *τ_em_, emission lifetimes*;

f *out of range for accurate determination*;

*^c−e^ samples degassed using the freeze–pump–thaw method*.

In the case of **Cu(Bph)** (Figure 2A, red), the intensities of both the ^1^MLCT and π-π^*^ bands were enhanced to 7500 and ~70000 M^−1^cm^−1^, respectively, with no shift observed for the ^1^MLCT band and a red-shift observed for the π-π^*^ band. Reports indicate that, for the similar types of heteroleptic Cu^I^ complexes, adding phenyl groups at the 4,7-positions of the dmp ligand, as in **Cu(ph)**, enhances the ^1^MLCT absorption band intensity. This enhancement results in a red shifts of the band to λ_abs_ = 388 nm (ε = 5700 M^−1^cm^−1^) from that of **Cu(H)** (λ_abs_ = 380 nm, ε = 2700 M^−1^cm^−1^) due to the enhancement in the corresponding transition dipole moment and stabilization of the π^*^ orbitals in the dmp ligand (Tsubomura et al., 2015). In our case, **Cu(Bph)** also retained this tendency, where the phenyl groups were modified from those in **Cu(ph)**, but to a much lesser degree.

Introducing NC- groups or NO_2_- groups at the 4-position of the two phenyl groups, as in **Cu(NCph)** and **Cu(NO**_**2**_**ph)** (Figure 2A, blue and purple), respectively, resulted in significant red shift in the ^1^MLCT absorption bands to 400 and 401 nm, with almost no changes observed in their intensities. Because these substituents are known as π-electron-withdrawing substituents, these red shifts indicate that the π^*^-orbitals on the dmp ligand were stabilized by these substituents through the π-systems containing the phenyl groups.

Such the red shifts of the ^1^MLCT bands were also observed for the Cu^I^ complexes featuring aryl substituents (Figure 2B). In the case of **Cu(3fu)** and **Cu(3th)** (Figure 2B, black and red, respectively), both ^1^MLCT bands underwent slight red shifts to 390 and 391 nm, respectively, while retaining their intensities, compared to **Cu(ph)**. This result indicates the different electronegativities of the O and S atoms made only negligible effect on the absorption spectra. **Cu(3Bzth)** also underwent a further red shift of the ^1^MLCT band than the other complexes in the form of a shoulder at 395 nm (Figure 2B, yellow). In this case, the π-π^*^ absorption band was also red shifted to 330 nm, indicating destabilized π orbitals over the substituted dmp ligand due to the expansion of the π-systems.

Compared to these aryl groups, substituting the 2-positions of the 5-membered rings with heteroatoms resulted in much larger red shifts in both the ^1^MLCT and π-π^*^ absorption bands. For **Cu(2th)** (Figure 2C, green), **Cu(2Bzth)**, and **Cu(Bz2fu)** (Figure 2C, blue and purple, respectively) gradual shifts in the ^1^MLCT bands of these complexes to longer wavelengths (400, 410, and 420 nm, respectively) occurred alongside an increase in the ε values to 6800, 8200, and 12300 M^−1^cm^−1^, respectively. Additionally, the π-π^*^ bands were also red shifted largely up to 350 nm. Thus, the π-conjugation over the substituted dmp ligand was effectively observed, especially for the complex featuring 2-benzofuryl substituents [**Cu(Bzfu)**]. These trends were almost similar in CH_3_CN solutions (Figure S1 and Table 2), although undesired generation of the corresponding homoleptic-type Cu^I^ complexes with ^1^MLCT absorption bands at over 450 nm obscured the original heteroleptic complexes.

**Table 2:** Photophysical Properties of the Cu^I^ Complexes in CH_3_CN.

Complex	λ_abs_^a^/nm (ε^b^/M^−1^ cm^−1^)	λ_em_^c^/nm	Φ_em_^d^	τ_em_^e^/μs
**Cu(H)**	375 (~2,300^f^)	575	0.021	0.96
**Cu(ph)**	384 (~6,000^f^)	590	0.027	0.99
**Cu(Bph)**	388sh (~7,200^f^)	596	0.028	1.2
**Cu(NCph)**	392 (~5,200^f^)	624	0.0073	0.19, 0.74
**Cu(NO**_**2**_**ph)**	390 (~6,300^f^)	620	0.0009	0.004, 0.89
**Cu(3fu)**	387 (~4,900^f^)	593	0.026	0.0015, 0.0075, 0.054, 1.37
**Cu(3th)**	386 (~6,000^f^)	596	0.041	1.26
**Cu(3Bzth)**	385sh (~6,100^f^)	600	0.042	0.98
**Cu(2th)**	390sh (~6,600^f^)	618	0.021	0.001, 0.006, 8.29
**Cu(2Bzth)**	400sh (~8,700^f^)	631	0.0093	0.005, 1.2, 14.1
**Cu(2Bzfu)**	410sh (~11,600^f^)	620sh, 650	0.0081	0.004, 32.2

a *λ_abs_, ^1^MLCT absorption maxima*;

b *ε, molar extinction coefficients*;

c *λ_em_, emission maxima*;

d *Φ_em_, emission quantum yield*;

e *τ_em_, emission lifetimes*;

f *minimum values considering partial decomposition to the corresponding homoleptic-type Cu^I^ complexes (see the spectra in Figure S1)*;

*^c−e^samples degassed using the freeze–pump–thaw method*.

**Figure 3:**
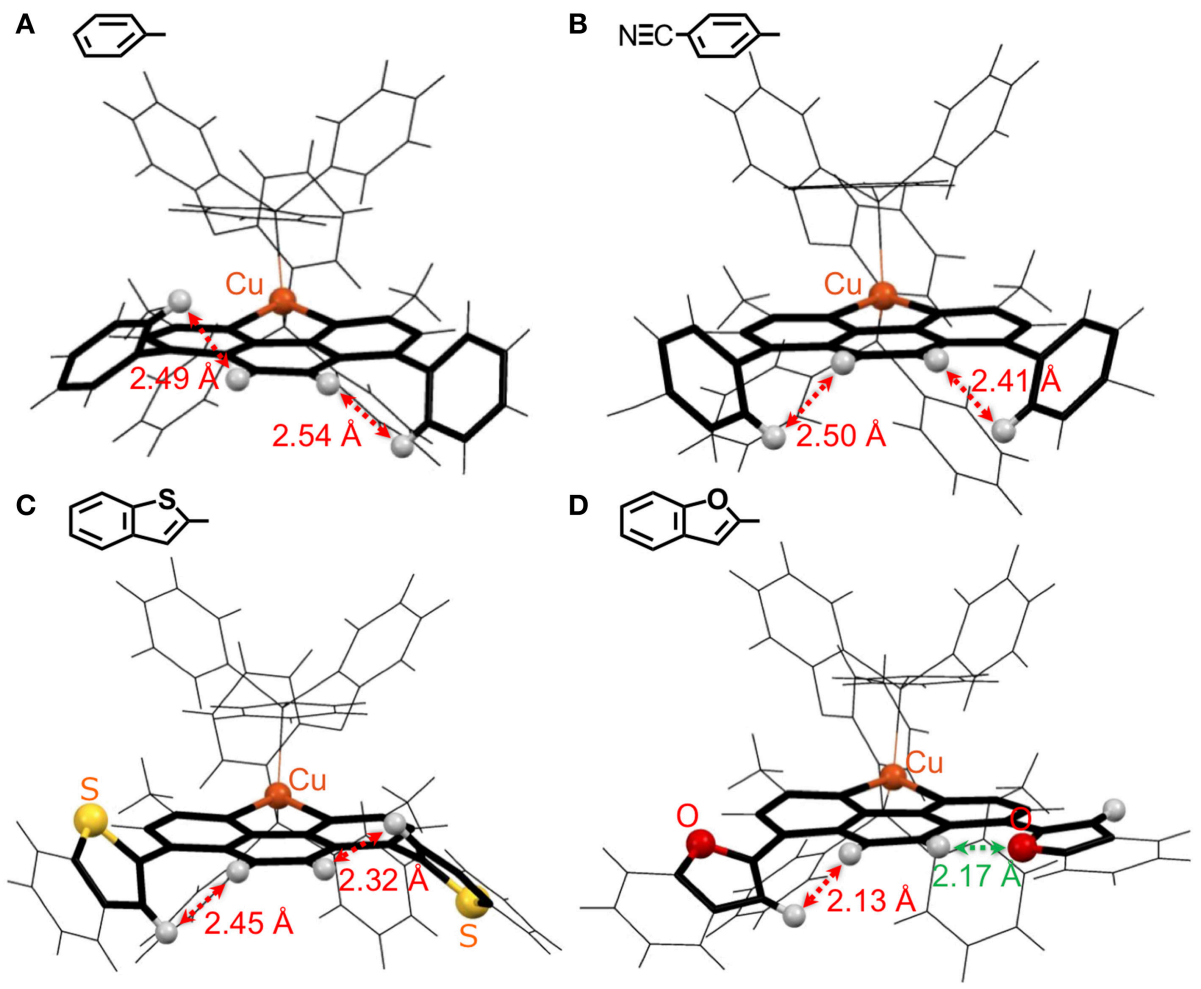
Wire model representations of the single-crystal Cu^I^-complexes: **(A) Cu(ph)**, **(B) Cu(NCph)**, **(C) Cu(2Bzth)**, and **(D) Cu(2Bzfu)**: the phen ligand and adjacent aryl substituents at the 4,7-positions are highlighted. The gray, yellow, red, and orange balls represent H atoms on the phen ligand and on the adjacent positions in the aryl rings, and the S, and O atoms on the aryl substituents, and Cu atoms, respectively.

Then, the single-crystal structures of these Cu^I^ complexes were determined to obtain information about the planarity of the substituted dmp ligand, which determines the overlap of the π^*^ orbitals between the aryl substituents and dmp. Here, we could obtain four types of single crystals of Cu^I^ complexes, **Cu(ph)**, **Cu(NCph)**, **Cu(2Bzth)**, and **Cu(2Bzfu)**. The structures of these are shown in Figure 3 and full analytical details are in the Supplementary Information (Figures S44–47 and Tables S2–6). All of these complexes have tetrahedral Cu^I^ center with an almost right angle between NCuN and PCuP planes (88.6, 89.0, 88.6, and 86.8°, respectively), a configuration required for these types of Cu^I^ complexes to exhibit strong emission. There are no remarkable differences in the complexes' residual parts, other than in the substituted dmp ligand, as follows.

The twisted angles between the phen and ph planes in the **Cu(ph)** crystal were found to be 60.2 and 53.9°. Because the H atoms at the 5,6-positions of the phen ligand and the 2,6-positions of the ph groups are in close contact, these ph groups cannot lie in the same plane as that of the phen ligand. Actually, the shortest distances between these H atoms were measured as 2.49 and 2.54 Å. These values are closing to the limitation of van der Waals radius of the H atom, at 1.2 Å (Bondi, 1964). Thus, the steric repulsion between these H atoms hinders the planarity of the ph-phen plane, resulting in a small expansion of the π-conjugation over the substituted dmp ligand. Even when π-electron withdrawing NC- groups were introduced at the 4-position of ph as for **Cu(NCph)**, the twist angles and shortest distances between the H atoms were 49.4 and 53.9°, and 2.50 and 2.41 Å, respectively. Thus, the steric hindrance of the H atoms should still play a role in hindering the NCph-phen bonds' planarity.

Complexes with 5-membered, heteroring substituents exhibited decreased twist angles. In the case of **Cu(2Bzth)**, the angles were 51.1 and 46.3°, maintaining the distances between the H atoms of 2.45 and 2.32 Å. However, the angles in **Cu(2Bzfu)** drastically decreased to 32.2 and 7.1°, shortening the H–H distance to 2.13 Å and changing the direction of O atom in another 2-benzofuryl substituent to neighbor to the 5-H atom of phen with the shorter distance of 2.17 Å. This means the substituents were able to lie in the same plane as the phen moiety. Because the van der Waals radius of the O atom is 1.52 Å, which is smaller than the 1.80 Å radius of the S atom (Bondi, 1964), the steric hindrance of the planarity over the substituted dmp ligand is not strong enough to result in a planner structure. Thus, the π-systems over the substituted dmp should be the strongest for **Cu(2Bzfu)** among the Cu^I^ complexes in this work. This structural accessibility of a planner structure should lower the π^*^ orbitals over the substituted dmp ligand, resulting in a red shift in the ^1^MLCT absorption band (Figure 2C, purple).

**Figure 4:**
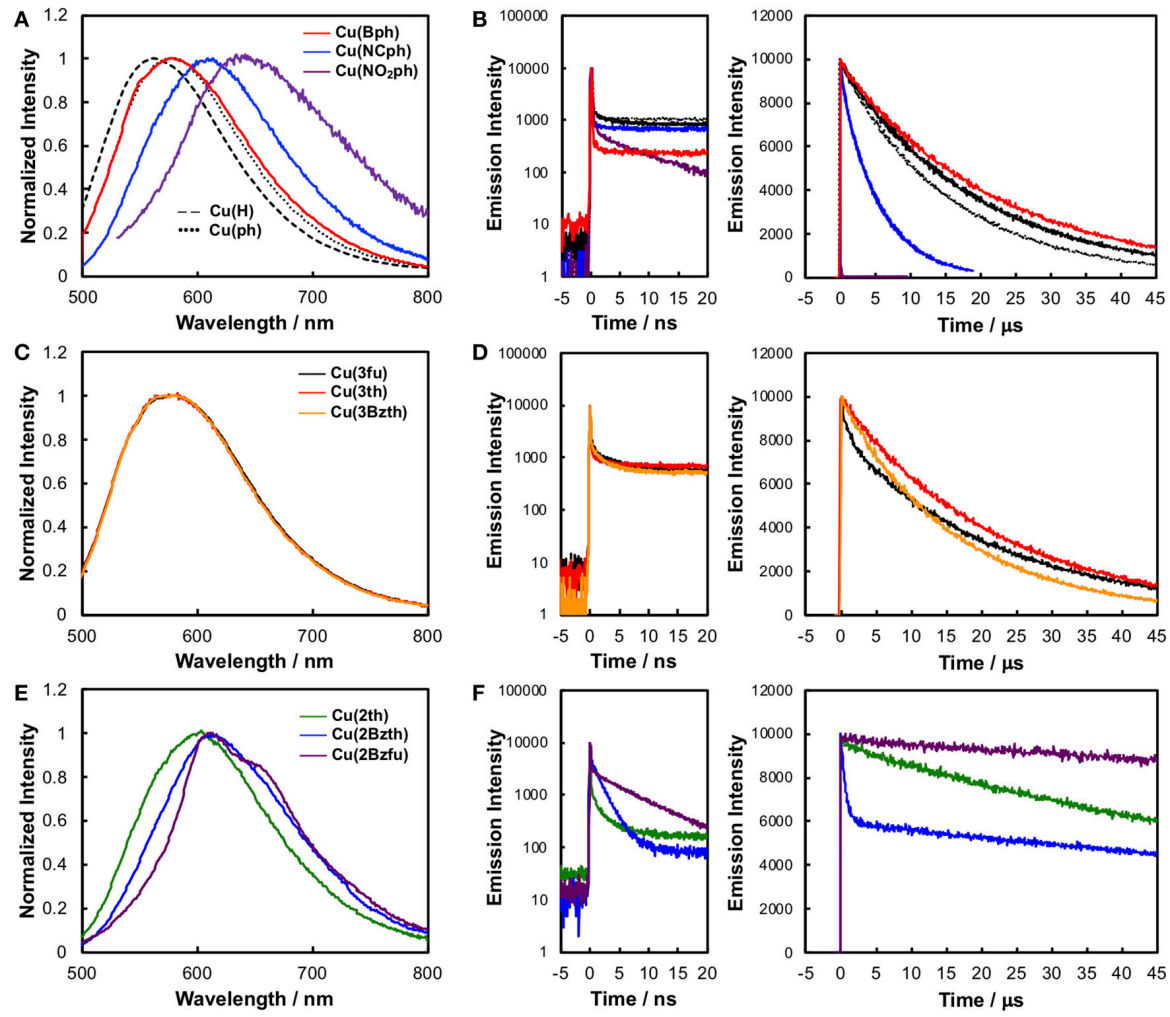
The corrected emission spectra **(A, C**, and **E)** and time-dependence data of the emission intensity **(B, D**, and **F)** of the Cu^I^ complexes in CH_2_Cl_2_ at RT. **(A)** Emission spectra and **(B)** time-dependence data of the intensity of **Cu(H)** (broken black line), **Cu(ph)** (dotted black line), **Cu(Bph)** (red), **Cu(NCph)** (blue), and **Cu(NO**_**2**_**ph)** (purple). **(C)** Emission spectra and **(D)** time-dependence data of the intensity of **Cu(3fu)** (black line), **Cu(3th)** (red), and **Cu(3Bzth)** (yellow). **(E)** Emission spectra and **(F)** time-dependence data of the intensity of **Cu(2th)** (green), **Cu(2Bzth)** (blue), and **Cu(2Bzfu)** (purple). Left panels on **(B, D**, and **F)** were observed from a shorter range of each right panel. The samples were degassed using the freeze–pump–thaw method. Table S1 summarizes the excitation and monitored wavelengths.

Figure 4 shows the emission spectra and time-dependences of the emission intensity, and the data are summarized in Table 1. All of the Cu^I^ complexes showed strong emission, apart from **Cu(NO**_**2**_**ph)**. In the case of **Cu(Bph)** (Figures 4A,B, red), the spectrum was almost the same as that of **Cu(ph)**, with a maximum (λ_em_) at 577 nm; however, its quantum yield (Φ_r_) of 0.60 was greater than that of **Cu(ph)**, which was 0.52. This is reasonable when considering the increased oscillator strength for the MLCT transition, as observed in the increased ε value of the ^1^MLCT absorption band in the UV-Vis spectrum. Thus, the radiative transition rate (*k*_r_) of the excited state should be enhanced, increasing the Φ_r_ because this value is defined in the context of *k*_r_ in all of the deactivation paths of the excited state (*k*_r_ + *k*_nr_ in this case). The increased, excited-state lifetime [τ_em_ = 1/(*k*_r_ + *k*_nr_)] for **Cu(Bph)** of 22.5 μs from that of **Cu(ph)** (19.4 μs) indicates that the *k*_nr_ value of **Cu(Bph)** is lower than that of **Cu(ph)**. This should be due to the increased mixing of the π-π^*^ excited state in the lowest MLCT state, which is indicated by a red shift in the intraligand ^1^π-π^*^ absorption band close to the ^1^MLCT band. Because these Cu^I^ complexes exhibit delayed fluorescence, the actual deactivation rates of *k*_r_ and *k*_nr_ could not be obtained in these measurements.

In the cases of **Cu(NCph)** and **Cu(NO**_**2**_**ph)** (Figures 4A,B, blue and purple, respectively), the emission maxima were red shifted to 609 and 640 nm, respectively, manifesting as red shifts in the corresponding ^1^MLCT absorption bands. In both cases, the Φ_r_ and τ_em_ values were significantly decreased [Φ_r_ = 0.16 and τ_em_ = 5.6 μs for **Cu(NCph)**], and **Cu(NO**_**2**_**ph)** showed almost no emission (Φ_r_ < 0.00, τ_em_ = 0.01 μs). These can be reasonably explained by the energy-gap law similarly to the substituent effects on the emission behaviors of α-diimine complexes of Ru and Re (Cook et al., 1984; Worl et al., 1991). The lowered energy of the MLCT transition resulted in a lower energy gap between the excited state and the ground state, thus leading to an increased *k*_nr_ value. **Cu(NO**_**2**_**ph)** exhibited almost no emissions, which can be attributed to charge localization on the strong, electron-withdrawing NO_2_ groups in the excited state, leading to a charge-separated radical-like state (Prei et al., 2015). This, in turn, should lead to an increase in the *k*_nr_ value.

The Cu^I^ complexes, substituted with smaller aryl groups, showed two different sets of behavior. The emission spectra of the **Cu(3fu)**, **Cu(3th)**, and **Cu(3Bzth)** complexes containing 5-membered ring systems with heteroatoms at 3-position (Figures 4C,D, black, red, and yellow, respectively), showed almost the same features as those found in the **Cu(ph)** spectrum. All of these complexes showed strong emissions with Φ_r_ values of 0.24–0.45 and τ_em_ values of ca. 20 μs, values that are almost the same as those of **Cu(ph)**. In these cases, changing the heteroatoms from O to S in the aryl rings appeared to have no great effect on the complexes' transition energies, but Φ_r_ value increased from 0.24 for **Cu(3fu)** to 0.35 for **Cu(3th)**, which further increased in **Cu(3Bzth)** (Φ_r_ = 0.45).

However, in systems where the heteroatoms featured at the 2-position of the rings, as in **Cu(2th)**, **Cu(2Bzth)**, and **Cu(2Bzfu)** (Figures 4E,F, green, blue, and purple for **Cu(2th)**, **Cu(2Bzth)**, and **Cu(2Bzfu)**, respectively), the emissions were drastically different from those of the other complexes. The emission spectra were observed to gradually red shift in the complexes in the aforementioned order and their shapes changed to vibronic shapes, particularly for **Cu(2Bzfu)**. Because the excitation spectrum from which this emission was monitored was in accordance with the UV-Vis absorption spectrum of **Cu(2Bzfu)** (Figure S3A), this emission could be confirmed to arise from the excited state of **Cu(2Bzfu)**.

This emission of **Cu(2Bzfu)** was found to be strongly air sensitive. If the emission spectrum was recorded for a sample prepared under an Ar atmosphere instead of a vacuum-degassed sample, the spectrum was observed to drastically change, resulting in a mismatch between the excitation and absorption spectra of **Cu(2Bzfu)** in CH_2_Cl_2_ (Figure S3B). However, this was not the case for the data recorded in a CH_3_CN solution, which showed no remarkable changes in the spectral features, even for a sample prepared under an Ar atmosphere (Figure S12C) compared to under vacuum conditions (Figure S3C).

Interestingly, these Cu^I^ complexes' τ_em_ values increased to over 100 μs, with a corresponding decrease in the Φ_r_ values. These results strongly indicate that the emissive lowest excited state changed from the MLCT state to another state, possibly the intraligand π-π^*^ excited state. This is a reasonable assumption, considering the red shift in the π-π^*^ transition band, as observed in the UV-Vis absorption spectra, from the π-expansion of the dmp ligand. Thus, in these complexes, the emission from the lowest excited state should arise from the delayed fluorescence of the intraligand ^3^π-π^*^ state.

The spectral feature depended strongly on the heteroatoms on the 2-positions of the aryl substituents. In the case of **Cu(2th)** and **Cu(2Bzth)**, the emission spectra were much broadened than that of **Cu(2Bzfu)**, although the lifetimes were still long. These results strongly indicate that the accessibility of the planar structure over the aryl substituted dmp enhances the π-conjugation over the dmp ligand. The similar phenomena about the long excited-state lifetime caused by the planarity between aryl substituents and α-diimine ligand has been discussed for emissive Ru complexes, in which the delocalization of negative charge over the substituted bpy (bpy = 2,2′-bipyridine) in the ^3^MLCT excited state lowers their structural changes from the ground state, leading to decreasing *k*_nr_ (Damrauer et al., 1997; Majewski et al., 2016). It is also reported that the Cu^I^(dmp) complex has the close-lying ^3^π-π^*^ state near the lowest ^3^MLCT excited state as for Cu^I^(dmp)(PPh_3_)${}_{2}^{+}$, which showed the vibronic emission spectrum at 77 K as a long-lifetime component as τ_em_ ~ 10 ms (Rader et al., 1981). Therefore, the long lifetime emission of **Cu(2th)**, **Cu(2Bzth)**, and **Cu(2Bzfu)** can be reasonably attributed to the participation of ^3^π-π^*^ excited state.

All of these tendencies were also valid even in CH_3_CN as a photocatalytic reaction solvent, except that the emission was significantly quenched compared to that observed in CH_2_Cl_2_ solutions due to “exciplex” formation, in which a solvent molecule coordinates to the Cu center as a fifth ligand in the excited-state Cu^I^ complexes (Figure S2 and Table 2). This type of quenching is characteristic of tetrahedral Cu^I^ complexes as the *d*^10^ Cu^I^ center decreases its charges upon closing to a *d*^9^ configuration in the MLCT excited state, changing the structure to planer one because of the Jahn-Teller effect (McMillin et al., 1985).

Thus, the quenching all of the Cu^I^ complexes studied in CH_3_CN indicates that they have MLCT character in their excited state to some extent, even for **Cu(2th)**, **Cu(2Bzth)**, and **Cu(2Bzfu)**, in which the excited state was mainly the π-π^*^ state. The extents of the MLCT character should increase especially in CH_3_CN because this state has CT in character, that are stabilized in a polarized solvent such as CH_3_CN more strongly than the intraligand π-π^*^ excited state. Actually, such the stabilizations were seen as red-shift of emission maxima in CH_3_CN compared to those in CH_2_Cl_2_. In the case of **Cu(NO**_**2**_**ph)**, the emission behavior completely changed in CH_3_CN, possibly as a result of photodecomposition, manifesting in a different excitation spectrum than **Cu(NO**_**2**_**ph)**'s original absorption spectrum.

**Figure 5:**
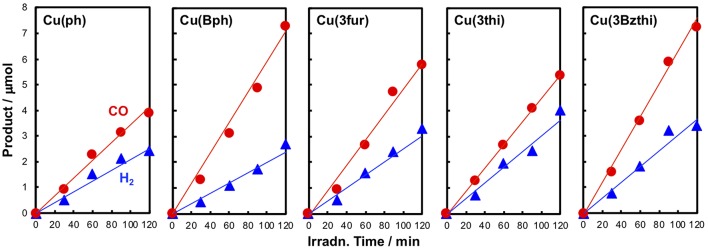
Time-course data of CO (red) and H_2_ (blue) evolution during the photocatalytic reactions using the Cu^I^ complexes as redox photosensitizers. A 4 ml CH_3_CN-TEOA (5:1 v/v) solution containing a Cu^I^ complex (0.5 mM), Fe(dmp)_2_(NCS)_2_ (0.05 mM), and BIH (10 mM) was irradiated using the 436 nm monochromatic light (intensity: 3 × 10^−8^ einstein s^−1^) of a Xe lamp under a CO_2_ atmosphere at 25°C.

Photocatalytic CO_2_ reduction reactions were performed using these newly designed Cu^I^ complexes as redox photosensitizers mixed with Fe^II^(dmp)_2_(NCS)_2_ (0.05 mM) as a catalyst and BIH (10 mM) as a reductant under a 436 nm monochromic light in a mixed solution of CH_3_CN–triethanolamine [TEOA, 5:1 (v/v)]. The Cu photosensitizer's concentration was fixed at 0.5 mM, showing almost no transparency at 436 nm in reaction cells with a 1-cm optical path length. Figure 5 shows the photocatalytic reaction results. The products were found to be only CO and H_2_, without the formation of any HCOOH as reported in a previous study performed under similar conditions (Takeda et al., 2016), and these compounds were linearly generated against the photoirradiation time for up to 2 h.

The photocatalytic properties were highly dependent on the Cu complex photosensitizer used (Table 3). When **Cu(Bph)** was used, the efficiencies were enhanced to Φ_r_ = 3.9 with H_2_ formation (Φ_r_ = 1.4). Take into account the low results for **Cu(H)** [Φ_r_ = 1.1 (Takeda et al., 2016)] and **Cu(ph)** (Φ_r_ = 2.3), increasing the number of the ph groups at the 4,7-positions of dmp tended to enhance the photosensitizing ability of these complexes for CO_2_ reduction.

**Table 3:** Quantum Yields of the Photocatalytic CO_2_ Reduction (Φ_r_) Using the Cu^I^ Complexes as Redox-Photosensitizer,^a^ Reduction Potentials (*E*_1/2_), and Excited-State Quenching Rate (*k*_q_) by BIH of the Cu^I^ Complexes.

Complex	*Φ*_r_		*E*_1/2_/V^b^	*k*_q_/10^−9^ M^−1^s^−1^	η_q_/%^c^
	**CO**	**H_**2**_**			
**Cu(H)**	1.1^d^	0.6 ^d^	−2.03^d^	4.4^d^	96^d^
**Cu(ph)**	2.3	1.5	−1.96^d^	5.6^d^	95^d^
**Cu(Bph)**	3.9	1.4	−1.92	7.6	99
**Cu(NCph)**	n.d.^e^	n.d.^e^	−1.75	9.9	95
**Cu(NO**_**2**_**ph)**	n.d.^e^	n.d.^e^	−1.31	– ^f^	– ^*f*^
**Cu(3fu)**	3.5	1.9	−1.96	10.6	99
**Cu(3th)**	2.9	2.1	−1.94	7.8	99
**Cu(3Bzth)**	4.1	2.0	−1.93	7.4	99
**Cu(2th)**	n.d.^e^	n.d.^e^	−1.83	9.0	100
**Cu(2Bzth)**	n.d.^e^	n.d.^e^	−1.74	7.9	100
**Cu(2Bzfu)**	n.d.^e^	n.d.^e^	−1.70	7.7	100

a *photocatalytic system consisting of Fe(dmp)_2_(NCS)_2_ (0.05 mM) as a catalyst and BIH (10 mM) as a reductant in CH_3_CN–TEOA (5:1 v/v) (see Figure 4)*;

b Potentials [V] vs. Ag/AgNO_3_ (0.01 M);

c η_q_, quenching fraction of the excited-state Cu^I^ complexes by BIH (10 mM), estimated by η_q_ = K_SV_[BIH]/(1 + K_SV_[BIH]), in which the K_SV_ [M^-1^] values are the slopes of the Stern-Volmer plots in Figures S5–S12;

d from Takeda et al. (2016);

e not determined due to the low product amount;

f *not detected due to weak emission*.

Such the enhancement in the photosensitizing ability was also confirmed in the cases of complexes featuring 5-membered ring systems with heteroatoms at the 3-position, such as **Cu(3fu)**, **Cu(3th)**, and **Cu(3Bzth)**. The most efficient case was **Cu(3Bzth)**, which showed Φ_r_ values of 4.1 and 2.0 for CO and H_2_ evolution, respectively. Even in the cases of **Cu(3fu)** and **Cu(3th)**, the efficiencies for the CO_2_ reduction were improved to Φ_r_ (CO) = 3.5 and 2.9, respectively, over those of **Cu(ph)**.

However, the Cu^I^ complexes containing 5-membered ring systems with heteroatoms at 2-position, such as **Cu(2th)**, **Cu(2Bzth)**, and **Cu(2Bzfu)**, showed no any photosensitizing ability in this reaction system. These complexes may have lower oxidative power in their excited state because their highest occupied molecular orbital (HOMO) energies should now increase since the π orbitals in the substituted dmp, as well as the *d* orbitals on the Cu^I^ center, are destabilized. Actually, the first oxidation wave in the cyclic voltammetry (CV) measurements, an indicator of the HOMO energy, showed positive shifts for **Cu(2th)**, **Cu(2Bzth)**, and **Cu(2Bzfu)** (shoulder waves around 0.9 V *vs*. Ag/AgNO_3_) compared to that of **Cu(ph)** (*E*_p_ = 0.92 V vs. Ag/AgNO_3_) (Figure S4).

The excited states of these Cu^I^ complexes were effectively quenched in the presence of BIH, except for that of **Cu(NO**_**2**_**ph)**, which means that all of these complexes might generate the corresponding one-electron reduced state through the reductive quenching of their excited states by BIH during photoirradiation (Tamaki et al., 2013; Takeda et al., 2018). The quenching rate constants (*k*_q_) were estimated to be ~10^10^ M^−1^s^−1^, corresponding to quantitative quenching (η_q_) under photocatalytic conditions with 10 mM of BIH (Table 3). Because the quenching behavior in terms of the emission lifetimes was in good agreement with the emission intensity changes, and no changes were observed in the UV-vis and emission spectra of the Cu^I^ complexes, even in the presence of BIH (Figures S5–S12). These quenching reactions can be attributed to a dynamic quenching process, in which the excited state reacts with BIH bimolecularly.

Then, *in situ* UV-Vis absorption spectral changes in the reaction solutions during the photocatalytic reactions were investigated and compared to those of the complexes recorded under an Ar atmosphere for up to 1 h of photoirradiation. In the cases of **Cu(3fu)**, **Cu(3th)**, and **Cu(3Bzth)** (Figures S15–17), which were good photosensitizers for photocatalytic CO_2_ reduction, almost no changes occurred in their spectra. This indicates that there was almost no decomposition of the Cu complexes in the photosensitizing cycles, donating electrons to the Fe catalyst, for the oxidation of BIH and CO_2_ reduction. Under an Ar atmosphere, the MLCT absorption bands of the original Cu^I^ complexes decreased just after the photoirradiation caused broad absorption up to 950 nm. These changes clearly indicate the original Cu^I^ complexes' photodecomposition. Because the one-electron reduced state produced by the reductive quenching of the excited state cannot donate to the CO_2_ reduction cycle of the Fe catalyst, the reduced species of the Cu^I^ complexes accumulate in the reaction solutions, decomposing due to their anionic radical character if CO_2_ is not present.

On the other hand, non-photosensitizing Cu^I^ complexes, such as **Cu(NCph)**, **Cu(2th)**, **Cu(2Bzth)**, and **Cu(2Bzfu)** (Figures S13, S18–20, respectively) showed signs of drastic decomposition even in the presence of CO_2_. This means that the Cu complexes cannot donate their electrons generated through the reductive quenching by BIH in the excited state for the CO_2_ reduction. Thus, these complexes showed no photocatalytic CO_2_ reduction properties. However, the lack of the photosensitizing ability of **Cu(NO**_**2**_**ph)** should be due to its unstable excited state because no changes were observed, neither under a CO_2_ nor an Ar atmosphere, meaning that no any redox cycles occurred under photoirradiation.

**Figure 6:**
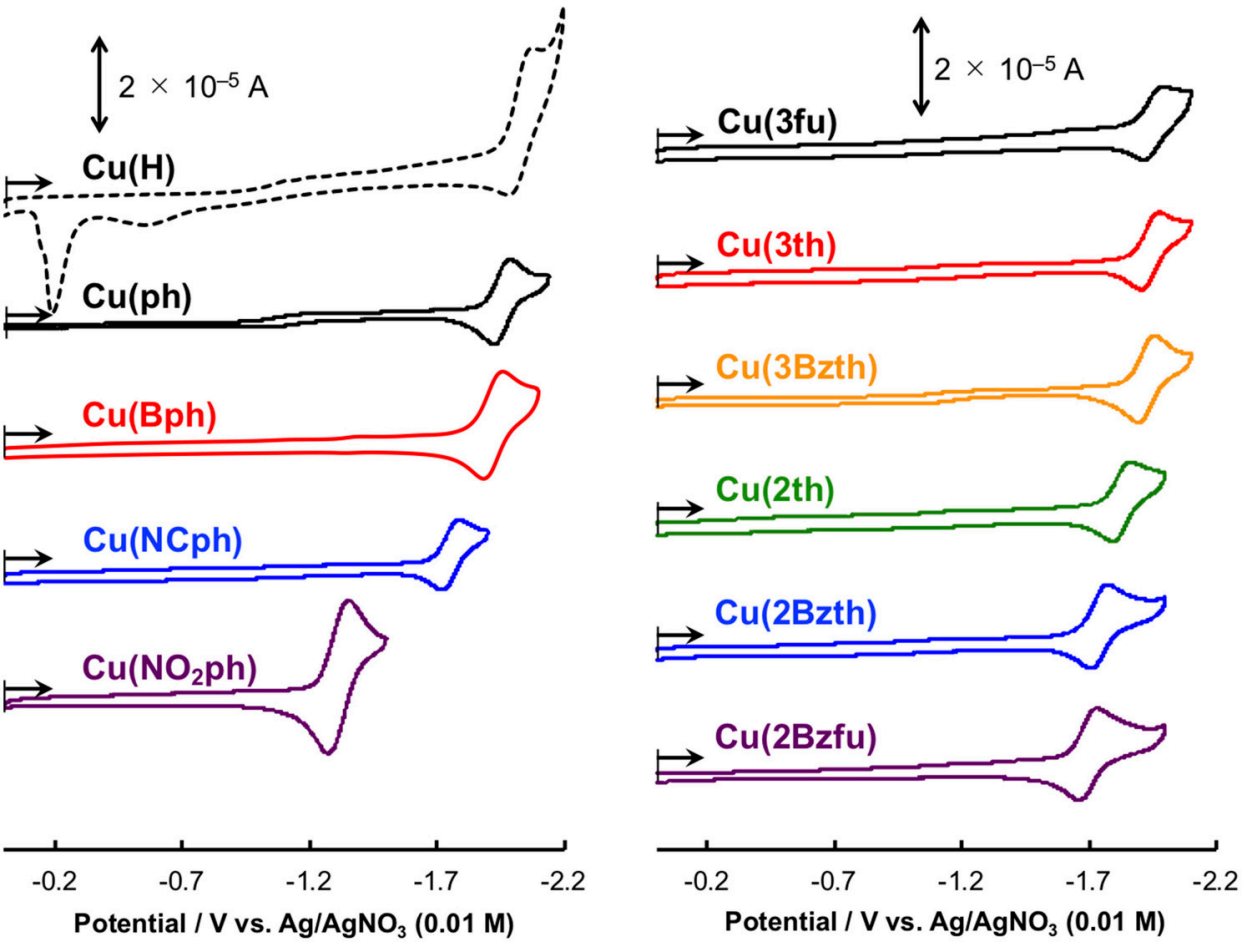
Cyclic voltammograms of the Cu^I^ complexes (0.5 mM) in CH_3_CN containing 0.1 M Et_4_NBF_4_ as a supporting electrolyte at a scan rate of 0.1 V s^−1^. WE: glassy carbon (Φ 3 mm); CE: Pt wire; RE: Ag/AgNO_3_ (0.01 M). The **Cu(H)** and **Cu(ph)** voltammograms are reproduced from ref. (Takeda et al., 2016) for comparison.

The Cu^I^ complexes' electrochemical properties were then examined, and the results are shown in Figure 6. All of the Cu^I^ complexes, apart from **Cu(H)**, showed a reversible redox couple in the CV as the first reduction wave, indicating that the corresponding one-electron reduced species was stabilized *via* the introduction of the aryl substituents on the dmp ligand, as observed for **Cu(ph)**. This is quite reasonable considering that the first reduction occurs in the dmp ligand's low-lying π^*^ orbital. However, for the CV of **Cu(NO**_**2**_**ph)**, the redox couple current was much higher without any sharpening. Thus, the first reduction wave in the CV of **Cu(NO**_**2**_**ph)** arises as a result of two sequential local electron reductions in the two NO_2_ph parts of one **Cu(NO**_**2**_**ph)**. The redox potentials (*E*_1/2_) of these first reduction waves are summarized in Table 3. Apparently, introducing electron-withdrawing groups, such as NCph and NO_2_ph, resulted in positive shifts in the *E*_1/2_ values up to −1.75 and −1.31 V vs. Ag/AgNO_3_ for **Cu(NCph)** and **Cu(NO**_**2**_**ph)**, respectively, indicating that the electron-donating ability of the corresponding reduced species was lowered. Those of the **Cu(Bph)**, **Cu(3fu)**, **Cu(3th)**, and **Cu(3Bzth)** complexes that have photosensitizing ability in this photocatalytic system maintained values more negative than −1.9 V, although these *E*_1/2_ values were positively shifted from those of **Cu(H)** and **Cu(ph)** (*E*_1/2_ = −2.03 and −1.96 V vs. Ag/AgNO_3_, respectively). For **Cu(2th)**, **Cu(2Bzth)**, and **Cu(2Bzfu)**, in which the π-system largely extends to the aryl substituents, exhibited more positive first reduction potentials of −1.83, −1.74, and −1.70 V, respectively, due to the stabilization of the π^*^ orbital over the substituted dmp ligand. Thus, the lowering energy of the π^*^ orbital on the dmp ligand *via* the introduction of phenyl rings with π-electron-withdrawing groups or 5-membered aryl groups was clearly confirmed.

Compared to the first reduction wave potential of Fe(dmp)_2_(NCS)_2_ in CH_3_CN–TEOA (5:1 v/v) solution (−1.61 V vs. Ag/AgNO_3_) under a CO_2_ atmosphere (Takeda et al., 2016), the reducing power of these Cu complexes is enough for them to donate their first single electron on the corresponding reduced species to the Fe catalyst. However, the decomposition behavior during the photoirradiation process indicates that the electron transfer to the Fe catalyst is inefficient, resulting in an accumulation of the reduced species of the Cu complexes and decomposition over longer time scales than those of the CV measurements. We are now continuing the photocatalytic reaction using the other catalysts for the CO_2_ reduction that function at a more positive potential.

## Conclusion

This study found the heteroleptic Cu^I^ complexes' visible-light absorption properties could be improved via expanding the dmp ligand's π-systems. Simple expansion using biphenyl substitution at the 4,7-popsitions of dmp enhanced ε without red shift of the ^1^MLCT band compared to that of the ph substituted one. Although π-electron accepting substituents such as 4-NCph or 4-NO_2_ph shifted the band, the λ_max_ values were still around 400 nm. The X-ray single crystal analysis showed the presence of protons at the 2,6-positions of the ph substituents slows the π-conjugation between the ph groups and dmp due to the steric hindrance of the 5,6-protons in the dmp ligand. Thus, substitution with 5-membered heterorings, with heteroatoms and no protons at the 2-positions, was enough to make improvements to the properties of the complexes. Thus, the complexes containing 2-benzofuryl groups having an O atom which size is smaller than a S atom at each 2-position showed a planner structure over the substituted dmp ligand, resulting in a red shift in the ^1^MLCT absorption bands up to 500 nm. Because the 3-thienyl and 3-furyl substitutions showed almost no effect on the light absorbing property, this red shift in **Cu(2Bzfu)** was not from a difference of electronegativities of the S and O atoms. Photocatalytic CO_2_ reduction reactions were carried out with the complexes in the presence of a Fe catalyst, showing quantum yields of up to 4% for the maximum amount of CO generated. The knowledge obtained in this study should help not only for further expansion of visible-light absorbing but also for designing the efficient redox-photosensitizer using the Cu^I^ complex to construct efficient earth-abundant system for the photocatalytic CO_2_ reduction.

## Experimental

### General Measurements

^1^H NMR spectra were recorded on a JEOL ECA-400II NMR spectrometer. The chemical shifts and coupling values arising from the second order effects of the protons in the phenanthroline derivatives were assigned by analyzing the obtained ^1^H NMR spectra using the iNMR (ver. 6.1.8) and WINDNMR (ver. 7.1.14) software (Reich, 1995). UV-vis absorption spectra were recorded on a JASCO V-565 or V-670 spectrometers. To measure the molar extinction coefficiencies (ε [M^−1^cm^−1^]), a 10 mL solution containing quantitatively weighted each Cu^I^ complexes (~2 mg) was prepared by dissolving in CH_2_Cl_2_ or CH_3_CN as a stock solution. An 1 mL of the stock solution was further diluted to 5 mL by each solvent and UV-vis absorption spectra of these two solutions were recorded using a cuvette cell having an 1-cm path length. After confirmation of no concentration effect on the spectral features indicating no dimer formation by comparison these two spectra, the absorbance was divided by each concentration to obtain ε value. The spectrum of the stock solution was used for assessment of the ^1^MLCT band and the latter was used for π-π^*^ absorption band because the detection range of absorbance (0.1−1.0) is suitable. Emission and excitation spectra were recorded on a JASCO FP8600 fluorescence spectrometer. Emission quantum yields of the Cu^I^ complexes in CH_3_CN were determined by a relative method, using an air-saturated CH_3_CN solution containing Ru(bpy)_3_(PF_6_) as a standard (Φ = 0.018) (Suzuki et al., 2009; Ishida and Beeby, 2016). Emission quantum yields in CH_2_Cl_2_ were collected using a Hamamatsu Photonics C9920–02G absolute photoluminescence quantum yield measurement system, consisting of a calibrated integrating sphere and a multi-channel spectrometer. Excited-state lifetimes were recorded on a HORIBA FluoroCube 1000U-S single-photon counting system. The sample solutions were degassed, *via* freeze–pump–thaw cycles, before the emission and lifetime measurements. For the quenching experiments, changes in the emission intensities and excited-state lifetimes of the Cu^I^ complexes in CH_3_CN, with or without various concentrations of BIH, were monitored under an Ar atmosphere. CVs were measured using Et_4_NBF_4_ (0.1 M) as a supporting electrolyte under an Ar atmosphere using a BAS CHI620EX or CHI760Es electrochemical analyzer with a glassy carbon working electrode (diameter, 3 mm), an Ag/AgNO_3_ (0.01 M) as a reference electrode, and a Pt counter electrode.

### Photocatalytic Reactions

A 4-mL CH_3_CN–TEOA (5:1 v/v) solution containing Fe(dmp)_2_(NCS)_2_ (0.05 mM), and the Cu photosensitizer (0.5 mM), and BIH (10 mM) as a reductant in a quartz cubic cell (1 cm path length; 11.0 mL volume) was bubbled with CO_2_ for 20 min. For the quantum yield determinations, a SHIMADZU QYM-01 photoreaction, quantum yield, evaluation system was adopted. The sample solution was irradiated using an Asahi Spectra Co. MAX-303 300-W Xe lamp with a 436 nm band-pass filter (FWHM: 10 nm), with the irradiated light intensity set at 3 × 10^−8^ einstein s^−1^ and the temperature of the reaction solution was maintained at 25 ± 0.1 °C using an IWAKI CTS-134A cooling thermo pump during the irradiation. The gaseous products, i.e., CO and H_2_, were analyzed using GC-TCD (GL science GC323) with an active carbon column. The amount of formic acid in the reaction solution with diluted water was analyzed using a capillary electrophoresis system (Otsuka Electronics Co. 7100L) with a buffer solution (pH 5.9) consisting of quinolinic acid, hexadecyltrimethylammonium hydroxide, and 2-amino-2-hydroxymethyl-1,3-propanediol as the electrolyte. To observe *in situ* UV-Vis spectral changes of the photocatalytic-reaction solution, the solution was irradiated, using an Ushio Optical Module high-pressure Hg lamp (BA-H500), with a 436 nm band-pass filter (FWHM = 10 nm), purchased from Asahi Spectra Co., and a CuSO_4_ solution (250 g L^−1^, 5 cm path length) filter with a 436 nm band-pass filter (FWHM: 10 nm) purchased from Asahi Spectra Co. Neutral density glass filters reduced the light intensity. The spectral changes were recorded on a Photal MCPD-6800 photodiode array detector equipped with a MC-2530 light source.

## Data Availability

All datasets generated for this study are included in the manuscript and/or the Supplementary Files.

## Author Contributions

HT and OI planned the all experiments. HT and YM synthesized the samples and performed the photophysical, electrochemical, and photocatalytic measurements. HT, HS, and HU analyzed the single crystal structures. HT wrote the manuscript and all authors have read and approved it.

### Conflict of Interest Statement

The authors declare that the research was conducted in the absence of any commercial or financial relationships that could be construed as a potential conflict of interest.

## Supplementary Material

Data Sheet 1 The Cu^I^ complexes' photophysical properties in CH_3_CN, emission and excitation spectra of **Cu(2Bzfu)** in CH_2_Cl_2_ under Ar or degassed atmospheres, positive scans of the cyclic voltammograms of the Cu^I^ complexes, quenching experiments of the excited state of the Cu^I^ complexes by BIH, *in situ* UV-Vis spectral changes during the photoirradiation reactions, experimental details such as materials, synthesis, ^1^H NMR spectra, peak analysis of the ^1^H NMR spectra of the newly synthesized ligands, and details of the single-crystal X-ray crystallography of **Cu(ph)**, **Cu(NCph)**, **Cu(2Bzth)**, and **Cu(2Bzfu)**.

Data Sheet 2 Crystallographic data for **Cu(ph)**, **Cu(NCph)**, **Cu(2Bzth)**, and **Cu(2Bzfu)** (CCDC numbers: 1895526-1895529) embedding RES and structure factors.
