# Supplementary material for: Development of Visible-Light Driven Cu(I) Complex Photosensitizers for Photocatalytic CO2 Reduction
Source: Front Chem. 2019 Jun 6;7:418. doi: 10.3389/fchem.2019.00418 (PMC6562897; doi:10.3389/fchem.2019.00418)
Supplement: Data Sheet 1 — The CuI complexes' photophysical properties in CH3CN, emission and excitation spectra of Cu(2Bzfu) in CH2Cl2 under Ar or degassed atmospheres, positive scans of the cyclic voltammograms of the CuI complexes, quenching experiments of the excited state of the CuI complexes by BIH, in situ UV-Vis spectral changes during the photoirradiation reactions, experimental details such as materials, synthesis, 1H NMR spectra, peak analysis of the 1H NMR spectra of the newly synthesized ligands, and details of the single-crystal X-ray crystallography of Cu(ph), Cu(NCph), Cu(2Bzth), and Cu(2Bzfu). [file Data_Sheet_1.pdf]

Supporting Information

Photophysical Properties of Cu^I Complexes in CH_3CN.

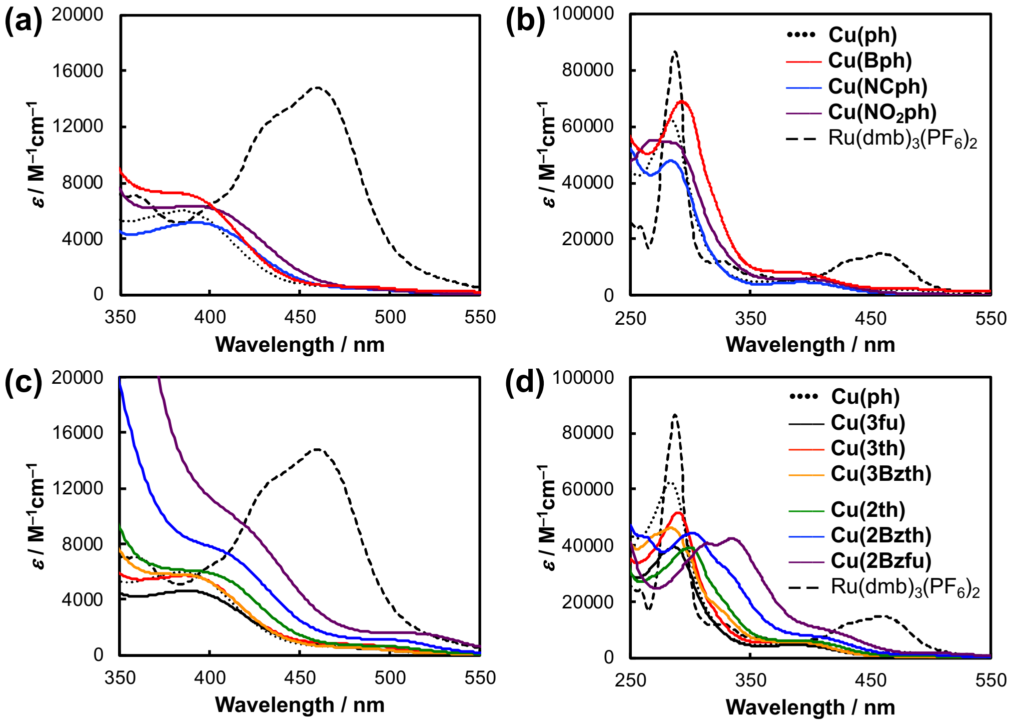

Figure S1. UV-Vis absorption spectra of the Cu^I complexes in CH_3CN alongside that of Ru(dmb)$_3$(PF$_6$)$_2$ in CH_3CN as a reference. (a) The ^{1}MLCT region of **Cu(ph)** (dotted black line), **Cu(Bph)** (red), **Cu(NCph)** (blue), **Cu(NO$_2$ph)** (purple), and Ru(dmb)$_3$(PF$_6$)$_2$ (broken black line). (c) The ^{1}MLCT region of **Cu(ph)** (dotted black line), **Cu(3fu)** (black), **Cu(3th)** (red), **Cu(3Bzth)** (yellow), **Cu(2th)** (green), **Cu(2Bzth)** (blue), **Cu(2Bzfu)** (purple), and Ru(dmb)$_3$(PF$_6$)$_2$ (broken black line). (b) and (d) show the full spectra of (a) and (b), respectively. The shoulder bands over 450 nm indicate partial decomposition to form the corresponding homoleptic-type Cu^I complexes. The **Cu(ph)** spectrum was reproduced from ref. S3 for comparison.

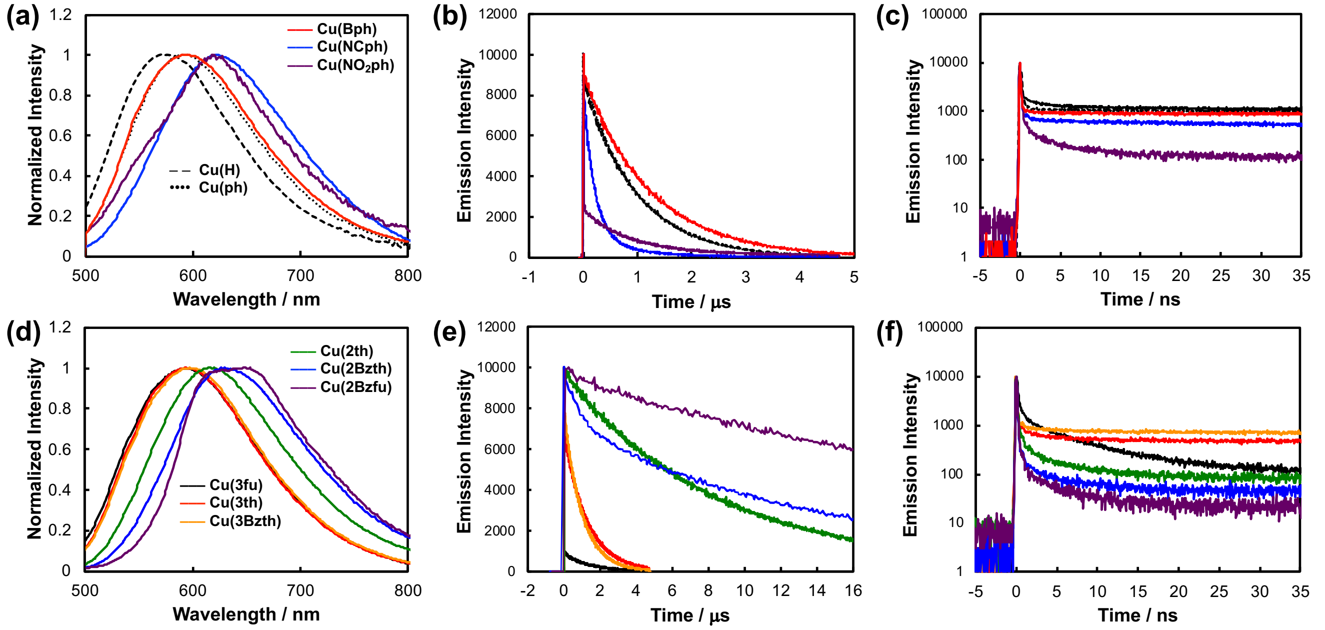

Figure S2. Corrected emission spectra (a and d) and time-dependences of the emission intensities (b, c, e, and f) of the Cu^{I} complexes in CH_3CN at RT. (a) Emission spectra and (b, c) time-dependences of the intensities of **Cu(H)** (broken black line), **Cu(ph)** (dotted black line), **Cu(Bph)** (red), **Cu(NCph)** (blue), and **Cu(NO$_2$ph)** (purple). (d) Emission spectra and (e, f) time-dependences of the intensities of **Cu(3fu)** (black line), **Cu(3th)** (red), **Cu(3Bzth)** (yellow), **Cu(2th)** (green), **Cu(2Bzth)** (blue), and **Cu(2Bzfu)** (purple). (c) and (f) were observed in the shorter range of (b) and (c), respectively. Table S1 summarizes the excitation and monitored wavelengths. **Cu(H)** and **Cu(ph)** emission spectra are reproduced from ref. S3 for comparison.

Table S1 Excitation (λ_{exc}) and Monitored (λ_{mon}) Wavelengths From the Emission Spectra and Time-Dependences Shown in Fig. 3 and Fig. S2.

Cu complex	in CH_2Cl_2			in CH_3CN		
	Emission	Time-dependence		Emission	Time-dependence	
	λ_{exc} / nm	λ_{exc} / nm	λ_{mon} / nm	λ_{exc} / nm	λ_{exc} / nm	λ_{mon} / nm
**Cu(H)**	379	379	562	379	379	575
**Cu(ph)**	379	379	575	379	379	590
**Cu(Bph)**	400	401	577	400	401	596
**Cu(NCph)**	444	444	605	400	444	625
**Cu(NO$_2$ph)**	444	444	640	415	444	620
**Cu(3fu)**	400	444	585	400	444	595
**Cu(3th)**	444	444	600	400	444	595
**Cu(3Bzth)**	400	444	575	400	444	600
**Cu(2th)**	444	444	575	415	444	615
**Cu(2Bzth)**	444	444	600	415	444	630
**Cu(2Bzfu)**	444	444	610	444	444	650

Emission and Excitation Spectra of Cu(2Bzfu) in CH_2Cl_2 under an Ar or Degassed Atmospheres.

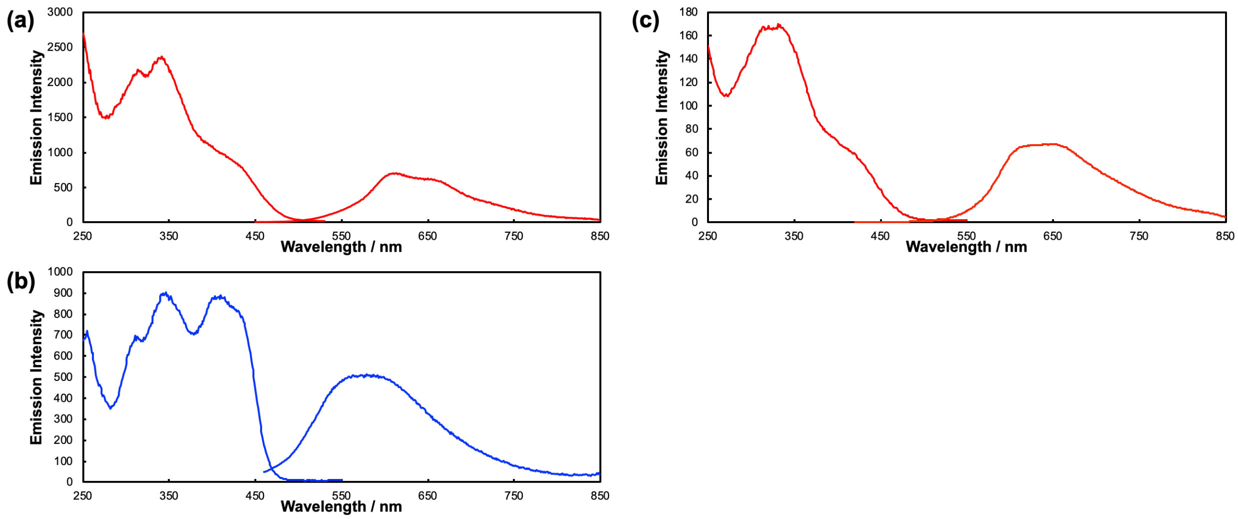

Figure S3. Corrected emission and excitation spectra of **Cu(2Bzfu)** at r.t. (a) Vacuum degassed (λ_{exc} = 444 nm, λ_{mon} = 610 nm) and (b) Ar bubbled (λ_{exc} = 444 nm, λ_{mon} = 580 nm) samples in CH_2Cl_2, and (c) vacuum-degassed sample (λ_{exc} = 415 nm, λ_{mon} = 610 nm) in CH_3CN.

Positive Scans of the Cyclic Voltammograms of the Cu^{I} Complexes.

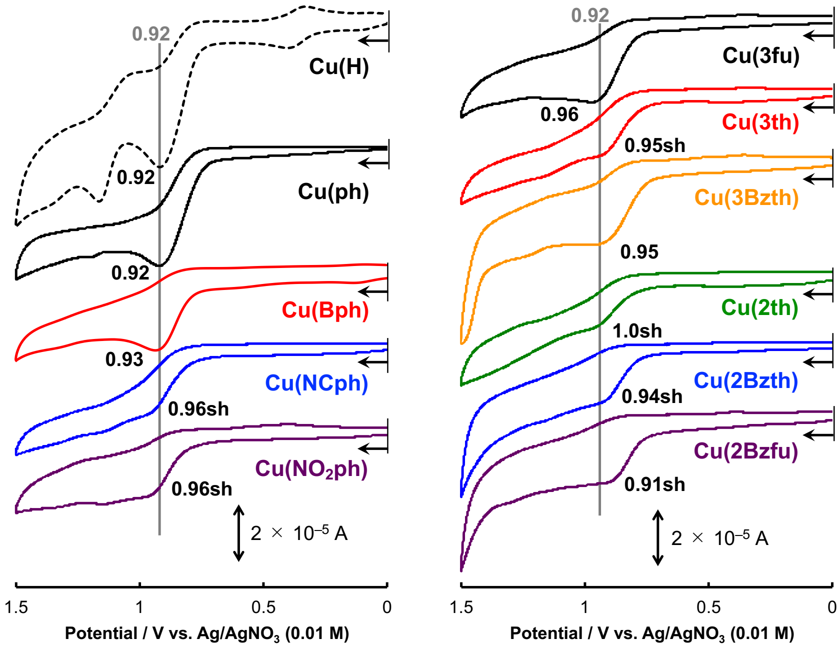

Figure S4. Cyclic voltammograms of the Cu^{I} complexes (0.5 mM) in CH_3CN containing 0.1 M Et_4NBF_4 as a supporting electrolyte at a scan rate of 0.1 V s^{-1}. WE: glassy carton (ϕ 3 mm); CE: Pt wire; RE: Ag/$AgNO_3$ (0.01 M).

Quenching Experiments of the Excited-State of Cu[I] Complexes by BIH.

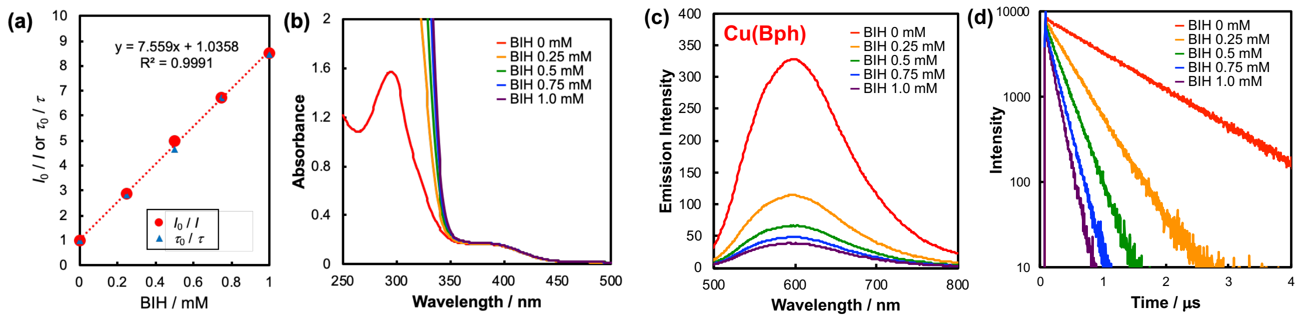

BIH / mM	I (600 nm)	τ / µs	I_0 / I	τ_0 / τ
0	327.5	1.00	1.00	1.00
0.25	114.3	0.36	2.87	2.82
0.5	65.9	0.22	4.97	4.65
0.75	48.8	0.15	6.72	6.71
1	38.4	0.12	8.52	8.47

Figure S5. Quenching experiment of the excited-state of **Cu(Bph)** by BIH: (a) Stern–Volmer plot, (b) UV-Vis absorption spectra, (c) emission spectra (λ_{exc} = 420 nm), and (d) time-dependence of the emission intensity (λ_{exc} = 444 nm, λ_{mon} = 595 nm) of Ar-bubbled CH_3CN solutions containing the same fixed amount of **Cu(Bph)** and varying amounts of BIH. The emission intensities (I) at the emission maxima (600 nm) in (c) and the emission lifetimes (τ) were obtained by analyzing the decay curves in (d) with a single exponential function are summarized in a table.

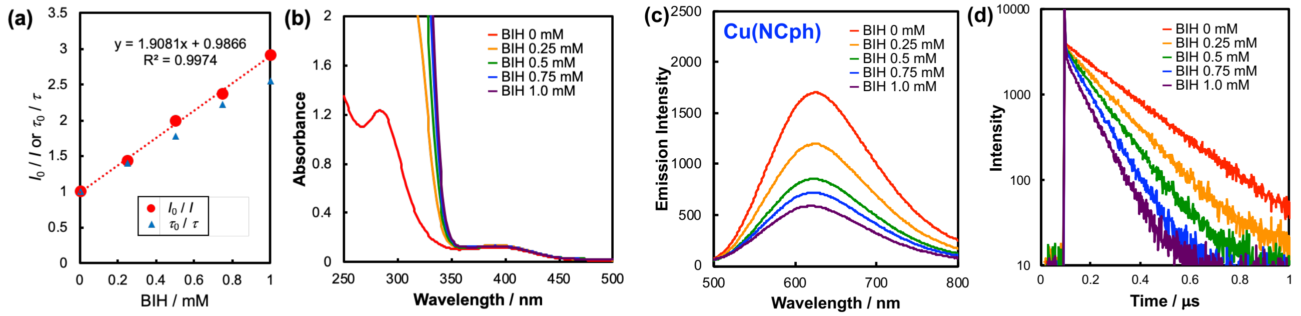

BIH / mM	I (624 nm)	τ / µs	I_0 / I	τ_0 / τ
0	1706	0.19	1.00	1.00
0.25	1197	0.14	1.43	1.40
0.5	857	0.11	1.99	1.78
0.75	717	0.087	2.38	2.22
1	586	0.075	2.91	2.55

Figure S6. Quenching experiment of the excited-state of **Cu(NCph)** by BIH: (a) Stern–Volmer plot, (b) UV-Vis absorption spectra, (c) emission spectra (λ_{exc} = 420 nm), and (d) time-dependence of the emission intensity (λ_{exc} = 444 nm, λ_{mon} = 625 nm) of Ar-bubbled CH_3CN solutions containing the same fixed amount of **Cu(NCph)** and varying amounts of BIH. The emission intensities (I) at the emission maxima (624 nm) in (c) and the emission lifetimes (τ) were obtained by analyzing the decay curves in (d) with a single exponential function are summarized in a table.

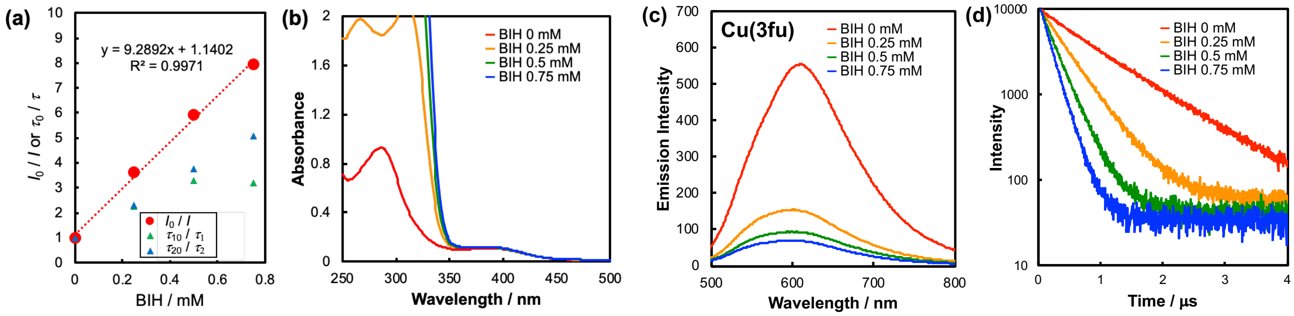

BIH / mM	I (605 nm)	τ_1 / µs	%	τ_2 / µs	%	I_0 / I	τ_{10} / τ_1	τ_{20} / τ_2
0	553	0.43	3.9	0.95	96.1	1.00	1.00	1.00
0.25	154	0.19	6.6	0.41	93.4	3.60	2.25	2.31
0.5	93	0.13	6.7	0.25	93.3	5.93	3.30	3.78
0.75	69	0.14	27.5	0.19	72.5	7.97	3.20	5.07

Figure S7. Quenching experiment of the excited-state of **Cu(3fu)** by BIH: (a) Stern–Volmer plot, (b) UV-Vis absorption spectra, (c) emission spectra (λ_{exc} = 420 nm), and (d) time-dependence of the emission intensity (λ_{exc} = 444 nm, λ_{mon} = 595 nm) of Ar-bubbled CH_3CN solutions containing the same fixed amount of **Cu(3fu)** and varying amounts of BIH. The emission intensities (I) at the emission maxima (605 nm) in (c) and the emission lifetimes (τ) obtained by analyzing the decay curves in (d) with a double exponential function are summarized in a table.

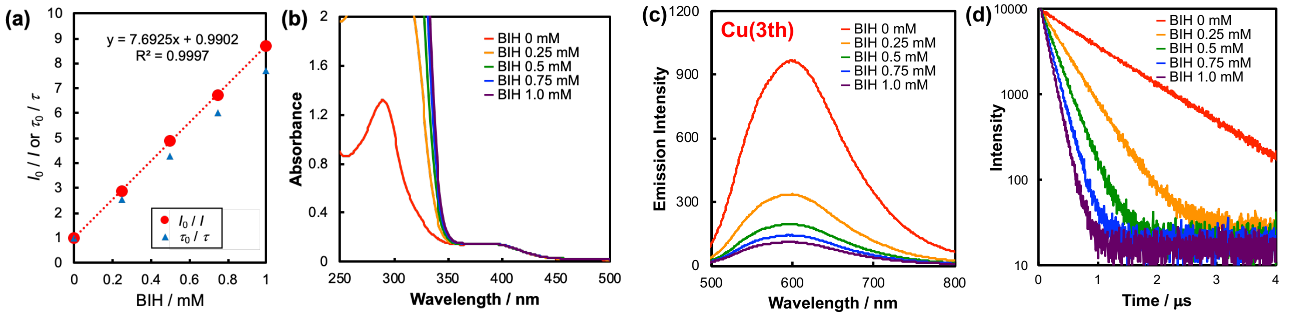

BIH / mM	I (593 nm)	τ / µs	I_0 / I	τ_0 / τ
0	962	0.98	1.00	1.00
0.25	335	0.38	2.87	2.56
0.5	196	0.23	4.91	4.29
0.75	144	0.16	6.70	6.02
1	111	0.13	8.70	7.73

Figure S8. Quenching experiment of the excited-state of **Cu(3th)** by BIH: (a) Stern–Volmer plot, (b) UV-Vis absorption spectra, (c) emission spectra (λ_{exc} = 420 nm), and (d) time-dependence of the emission intensity (λ_{exc} = 444 nm, λ_{mon} = 595 nm) of Ar-bubbled CH_3CN solutions containing the same fixed amount of **Cu(3th)** and varying amounts of BIH. The emission intensities (I) at the emission maxima (593 nm) in (c) and the emission lifetimes (τ) obtained by analyzing the decay curves in (d) with a single exponential function are summarized in a table.

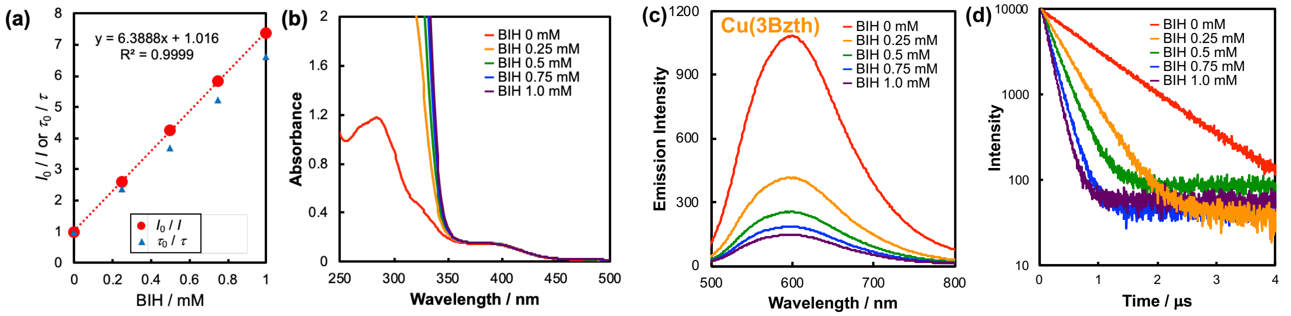

BIH / mM	I (593 nm)	τ / μs	I_0 / I	τ_0 / τ
0	1078	0.87	1.00	1.00
0.25	414	0.36	2.60	2.39
0.5	254	0.24	4.25	3.69
0.75	185	0.17	5.83	5.22
1	146	0.13	7.37	6.62

Figure S9. Quenching experiment of the excited-state of **Cu(3Bzth)** by BIH: (a) Stern–Volmer plot, (b) UV-Vis absorption spectra, (c) emission spectra (λ_{exc} = 420 nm), and (d) time-dependence of the emission intensity (λ_{exc} = 444 nm, λ_{mon} = 625 nm) of Ar-bubbled CH_3CN solutions containing the same fixed amount of **Cu(3Bzth)** and varying amounts of BIH. The emission intensities (I) at the emission maxima (593 nm) in (c) and the emission lifetimes (τ) obtained by analyzing the decay curves in (d) with a single exponential function are summarized in a table.

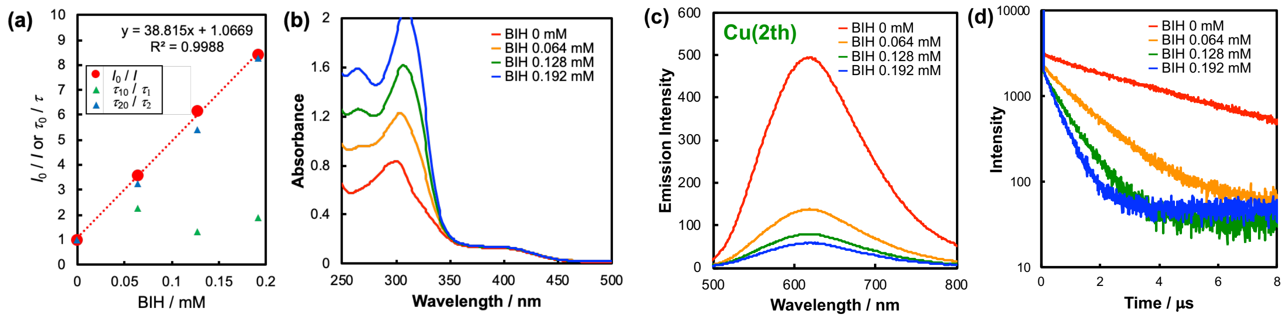

BIH / mM	I (624 nm)	τ_1 / μs	%	τ_2 / μs	%	I_0 / I	τ_{10} / τ_1	τ_{20} / τ_2
0	491	0.48	1.0	4.3	99.0	1.00	1.00	1.00
0.064	137	0.21	1.2	1.3	98.8	3.58	2.25	3.24
0.128	79	0.37	7.5	0.80	92.5	6.18	1.31	5.39
0.192	58	0.25	4.8	0.52	95.2	8.41	1.91	8.29

Figure S10. Quenching experiment of the excited-state of **Cu(2th)** by BIH: (a) Stern–Volmer plot, (b) UV-Vis absorption spectra, (c) emission spectra (λ_{exc} = 420 nm), and (d) time-dependence of the emission intensity (λ_{exc} = 444 nm, λ_{mon} = 630 nm) of Ar-bubbled CH_3CN solutions containing the same fixed amount of **Cu(2th)** and varying amounts of BIH. The emission intensities (I) at the emission maxima (624 nm) in (c) and the emission lifetimes (τ) obtained by analyzing the decay curves in (d) with a double exponential function are summarized in a table.

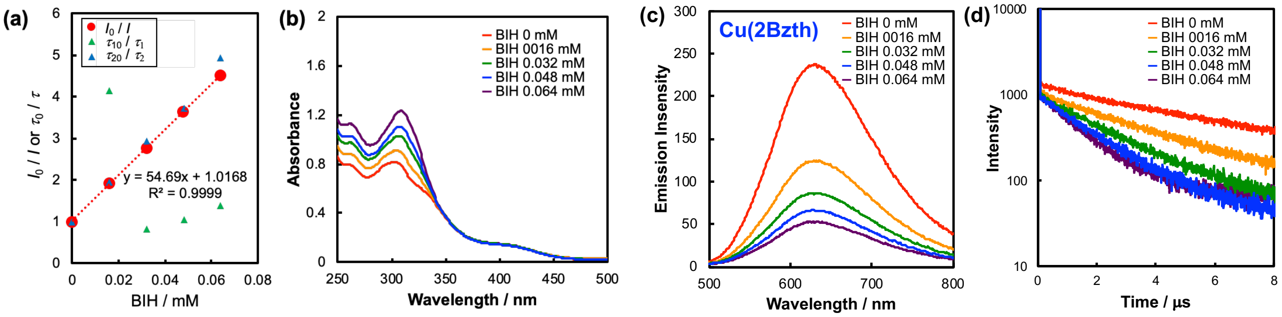

BIH / mM	I (631 nm)	τ_1 / µs	%	τ_2 / µs	%	I_0 / I	τ_{10} / τ_1	τ_{20} / τ_2
0	238	0.94	1.9	6.9	98.1	1.00	1.00	1.00
0.016	124	0.23	0.5	3.5	99.5	1.92	4.15	1.96
0.032	86	1.1	1.4	2.4	98.6	2.77	0.82	2.93
0.048	66	0.90	3.9	1.9	96.1	3.63	1.04	3.74
0.064	53	0.67	3.5	1.4	96.5	4.52	1.40	4.94

Figure S11. Quenching experiment of the excited-state of **Cu(2Bzth)** by BIH: (a) Stern–Volmer plot, (b) UV-Vis absorption spectra, (c) emission spectra (λ_{exc} = 420 nm), and (d) time-dependence of the emission intensity (λ_{exc} = 444 nm, λ_{mon} = 630 nm) of Ar-bubbled CH_3CN solutions containing the same fixed amount of **Cu(2Bzth)** and varying amounts of BIH. The emission intensities (I) at the emission maxima (631 nm) in (c) and the emission lifetimes (τ) obtained by analyzing the decay curves in (d) with a double exponential function are summarized in a table.

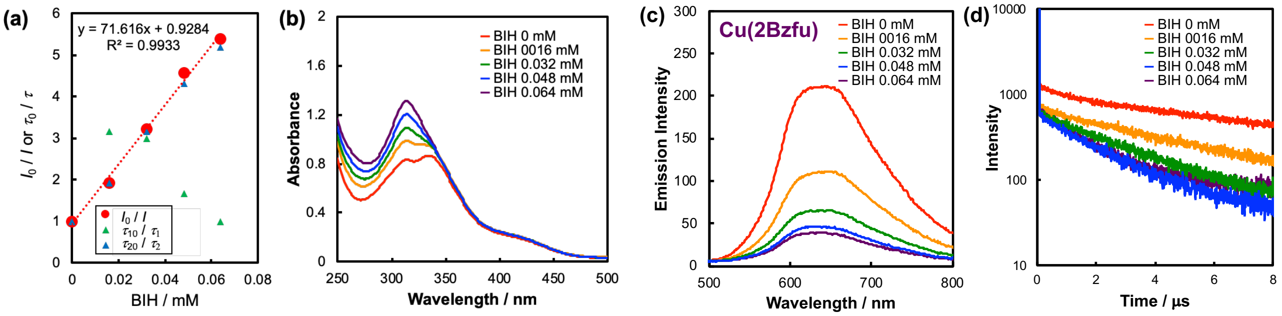

BIH / mM	I (640 nm)	τ_1 / µs	%	τ_2 / µs	%	I_0 / I	τ_{10} / τ_1	τ_{20} / τ_2
0	210	0.73	2.2	9.3	97.8	1.00	1.00	1.00
0.016	110	0.23	0.8	4.9	99.2	1.91	3.15	1.92
0.032	65	0.24	0.6	3.0	99.4	3.22	2.98	3.16
0.048	46	0.44	1.5	2.2	98.5	4.57	1.67	4.30
0.064	39	0.74	13.0	1.8	87.0	5.40	0.99	5.19

Figure S12. Quenching experiment of the excited-state of **Cu(2Bzfu)** by BIH: (a) Stern–Volmer plot, (b) UV-Vis absorption spectra, (c) emission spectra (λ_{exc} = 420 nm), and (d) time-dependence of the emission intensity (λ_{exc} = 444 nm, λ_{mon} = 630 nm) of Ar-bubbled CH_3CN solutions containing the same fixed amount of **Cu(2Bzfu)** and varying amounts of BIH. The emission intensities (I) at the emission maxima (640 nm) in (c) and the emission lifetimes (τ) obtained by analyzing the decay curves in (d) with a double exponential function are summarized in a table.

***In situ* UV-Vis spectral changes during the photoirradiation reactions.**

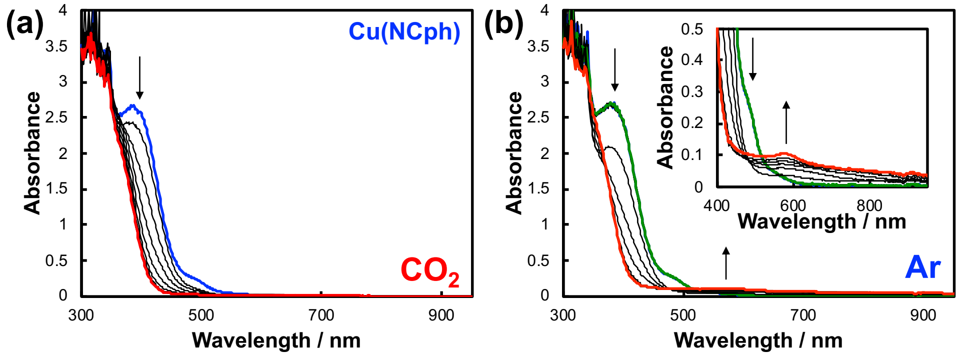

Figure S13. UV-Vis spectral changes during photoirradiation reactions using a **Cu(NCph)** as a photosensitizer. A CH_3CN–TEOA (5:1 v/v) solution containing **Cu(NCph)** (0.5 mM), $Fe(dmp)_2(NCS)_2$ (0.05 mM), and BIH (10 mM) was irradiated using the 436-nm monochromatic light of a Hg lamp under CO_2 (a) and under an Ar atmosphere (b). The blue and red lines show the spectra before and after 1 h of photoirradiation, respectively, at 5 min intervals for (a) and 5 sec intervals until 30 sec (green line) and 10 min intervals from 30 sec to 1 h (red line) for (b).

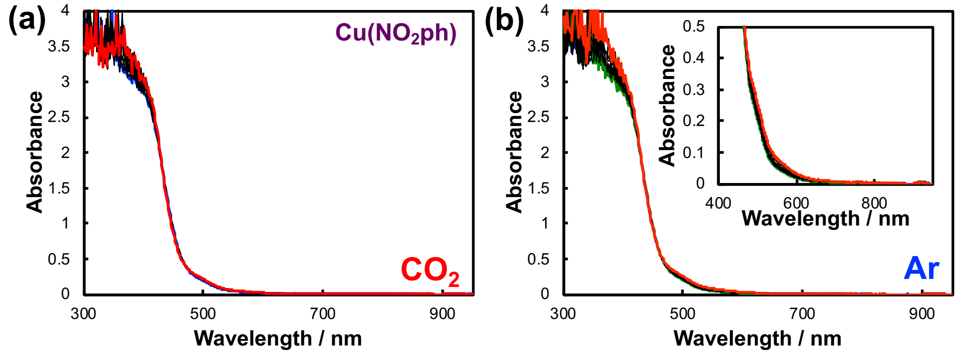

Figure S14. UV-Vis spectral changes during photoirradiation reactions using a **Cu(NO_2ph)** as a photosensitizer. The reaction conditions are same as those detailed in Fig. S13.

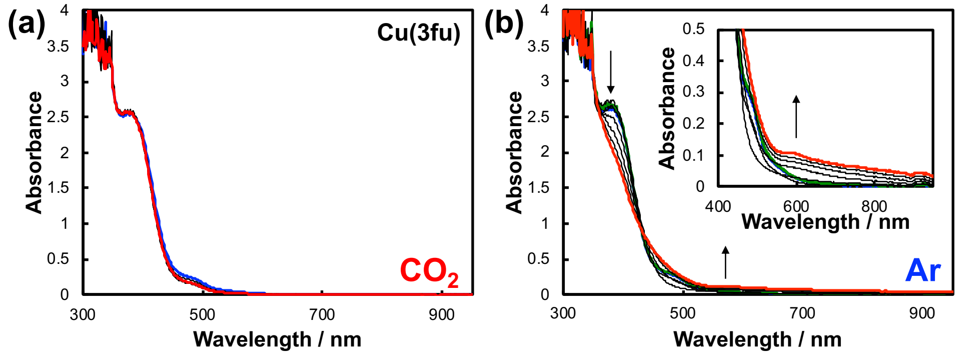

Figure S15. UV-Vis spectral changes during photoirradiation reactions using a **Cu(3fu)** as a photosensitizer. The reaction conditions are same as those detailed in Fig. S13.

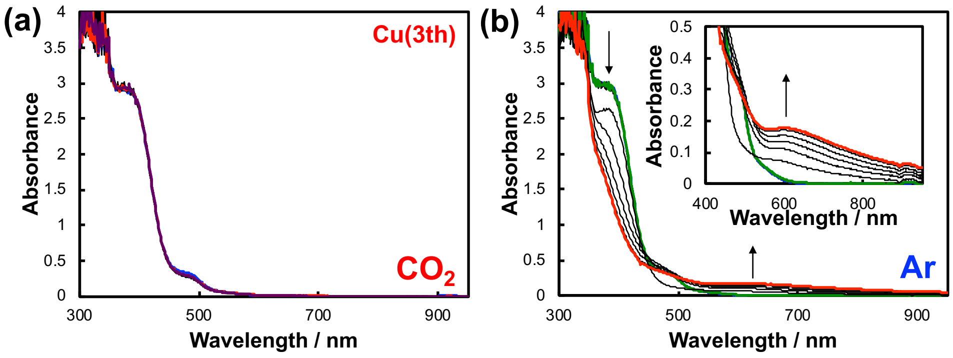

Figure S16. UV-Vis spectral changes during photoirradiation reactions using a **Cu(3th)** as a photosensitizer. The reaction conditions are same as those detailed in Fig. S13.

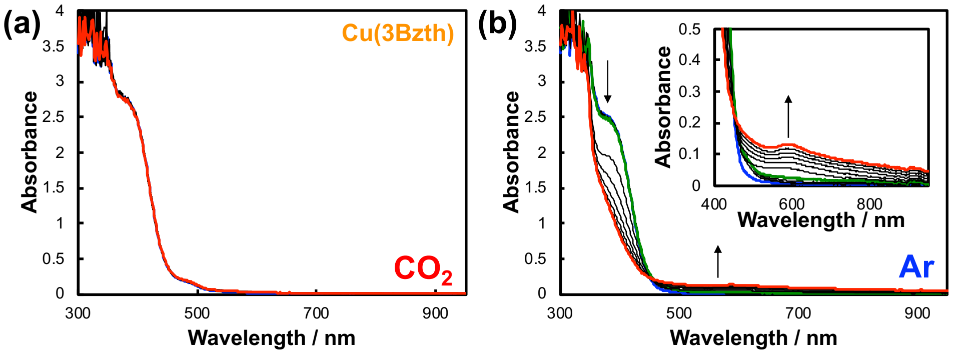

Figure S17. UV-Vis spectral changes during photoirradiation reactions using a **Cu(3Bzth)** as a photosensitizer. The reaction conditions are same as those detailed in Fig. S13.

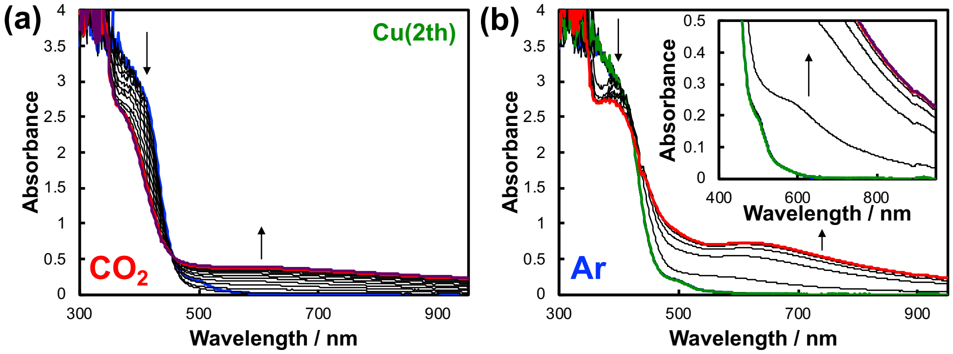

Figure S18. UV-Vis spectral changes during photoirradiation reactions using a **Cu(2th)** as a photosensitizer. The reaction conditions are same as those detailed in Fig. S13.

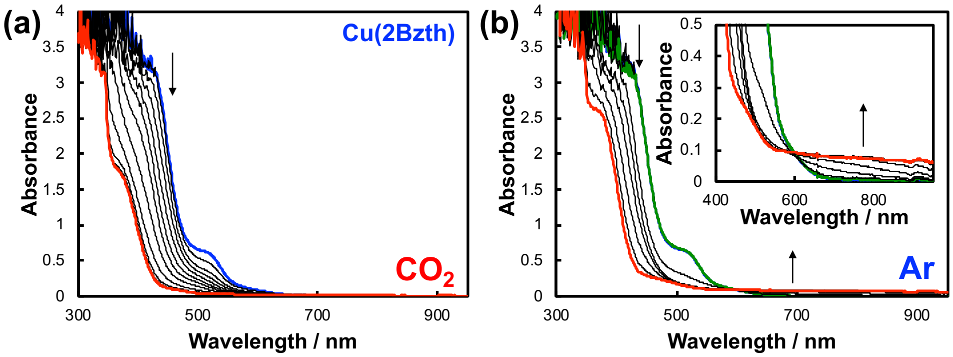

Figure S19. UV-Vis spectral changes during photoirradiation reactions using a **Cu(2Bzth)** as a photosensitizer. The reaction conditions are same as those detailed in Fig. S13.

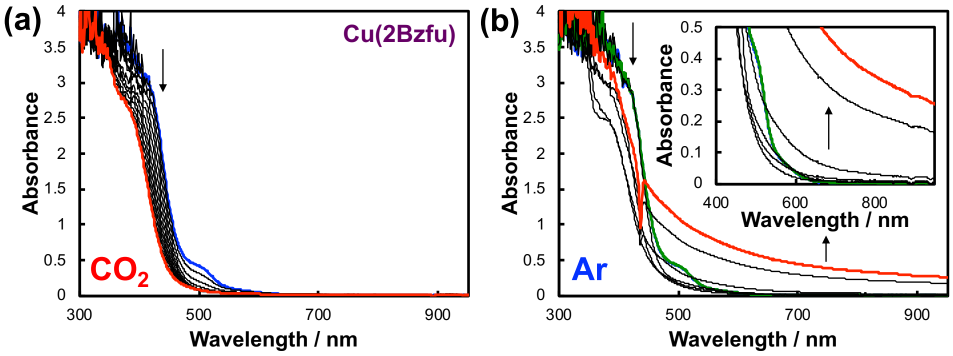

Figure S20. UV-Vis spectral changes during photoirradiation reactions using a **Cu(2Bzfu)** as a photosensitizer. The reaction conditions are same as those detailed in Fig. S13.

Experimental Details.

Materials.

The ligands 2,9-dimethyl-1,10-phenahthroline (dmp) and 4,7-diphenyl-2,9-dimethyl-1,10-phenahthroline (bathocuproine: bcp) were purchased commercially and used without further purification. 4,7-dichloro-2,9-dimethyl-1,10-phenanthroline (**dmp-Cl**) was synthesized according to a procedure referenced in the literatures.[S1] $[Cu^{I}(CH_3CN)_4](PF_6)$,[S2] **Cu(H)**,[S3,S4] **Cu(ph)**,[S3,S5] $Fe^{II}(dmp)(NCS)_2$,[S6] $Ru(bpy)_3(PF_6)_2$,[S7] and $Ru(dmb)_3(PF_6)_2$[S7,S8] were prepared according to literature procedures. BIH was synthesized according to a reported procedure.[S9] Tetraethylammonium tetrafluoroborate (Et_4NBF_4) was dried in vacuo at 100 °C overnight before use. CH_3CN was distilled three times over P_2O_5 and

then over CaH_2 just before use. TEOA was distilled under a reduced pressure under an Ar atmosphere. Other reagents and solvents were of the highest commercial quality and used without further purification.

Synthesis.

4,7-di([1,1'-biphenyl]-4-yl)-2,9-dimethyl-1,10-phenanthroline ***(dmp-Bph)*** To a mixture of **dmp-Cl** (502 mg, 1.81 mmol), 4-biphenylboronic acid (865 mg, 4.37 mmol), and *N,N'*-dimethylformamide (DMF, 40 ml), an aqueous solution (10 ml) containing Na_2CO_3 (1.5 M) was added and degassed by N_2 bubbling. Then, $Pd(PPh_3)_4$ (97.0 mg, 0.0839 mmol) and EtOH (0.89 ml) were added and the solution was refluxed for one night under a N_2 atmosphere. After cooling to room temperature (r.t.), a 0.1 M NaOH aqueous solution (100 ml) was added to the reaction mixture and the product was extracted using CH_2Cl_2 and washed with water several times. The collected CH_2Cl_2 phase was dried with Na_2SO_4, and the solvent was removed with a rotary evaporator. The resulting white powder was dried *in vacuo*. Yield: 717 mg (77.2%). ^{1}H NMR (chloroform-*d*): δ (ppm) 7.86 (2H, s, phen-*5*), 7.75 (2H, A1A1'X1X1'm, *J*(A1X1) = 8.5 Hz, *J*(A1A1') = 2.2 Hz, *J*(A1X1') = 0.5 Hz, bph-*2* (A1)), 7.75 (2H, A1A1'X1X1'm, *J*(A1'X1') = 8.5 Hz, *J*(A1A1') = 2.2 Hz, *J*(X1A1') = 0.5 Hz, bph-*6* (A1')), 7.69 (2H, A2A2'M2M2'X2m, *J*(A2M2) = 8.0 Hz, *J*(A2X2) = 1.2 Hz, *J*(A2A2) = 1.0 Hz, *J*(A2M2') = 0.5 Hz, bph-*2′* (A2)), 7.68 (2H, A2A2'M2M2'X2m, *J*(A2'M2') = 8.6 Hz, *J*(X2A2') = 1.2 Hz, *J*(A2A2') = 1.0 Hz, *J*(M2A'2) = 0.5 Hz, bph-*6′* (A2')), 7.62 (2H, A1A1'X1X1'm, *J*(A1'X1') = 8.5 Hz, *J*(X1X1') = 2.2 Hz, *J*(A1X1') = 0.5 Hz, bph-*5* (X1')), 7.62 (2H, A1A1'X1X1'm, *J*(A1X1) = 8.5 Hz, *J*(X1X1') = 2.2 Hz, *J*(X1A1') = 0.5 Hz, bph-*3* (X1)), 7.50 (2H, s, phen-3), 7.49 (2H, A2A2'M2M2'X2m, *J*(A2'M2') = 8.6 Hz, *J*(X2M2') = 7.5 Hz, *J*(M2M2') = 2.0 Hz, *J*(A2M2') = 0.5 Hz, bph-*5′* (M2')), 7.49 (2H, A2A2'M2M2'X2m, *J*(A2M2) = 8.0 Hz,

J(M2X2) = 7.5 Hz, J(M2M2') = 2.0 Hz, J(M2A2') = 0.5 Hz, bph-*3'* (M2)), 7.40 (2H, A2A2'M2M2'X2m, J(X2M2') = J(M2X2) = 7.5 Hz, J(A2X2) = J(X2A2') = 1.2 Hz, bph-*4'* (X2)), 3.02 (6H, s, phen-CH_3).

4,4'-(2,9-dimethyl-1,10-phenanthroline-4,7-diyl)dibenzonitrile ***(dmp-NCph)*** To a mixture of **dmp-Cl** (100 mg, 0.36 mmol), (4-cyanophenyl)boronic acid (132.2 mg, 0.89 mmol), and *N,N'*-dimethylacetoamide (DMA, 7 ml), an aqueous solution (1.8 ml) containing Na_2CO_3 (330 mg, 3.1 mmol) was added and degassed by N_2 bubbling. Then, $Pd(PPh_3)_4$ (16.6 mg, 0.0144 mmol) and EtOH (0.89 ml) were added and the solution was refluxed for one night under a N_2 atmosphere. After cooling to r.t., a 0.1 M NaOH aqueous solution (10 ml) was added to the reaction mixture and the product was extracted using CH_2Cl_2 and washed with water several times. The collected CH_2Cl_2 phase was dried with Na_2SO_4, and the solvent was removed with a rotary evaporator. Column chromatography on alumina (2.5 cm × 11 cm; aluminum oxide 90 standard (Merck)) with CH_2Cl_2 provided a solution containing **dmp-NCph**. The product was purified further by reprecipitation, whereby a white powder was precipitated from a saturated CH_2Cl_2–EtOH (9:1 v/v) solution *via* the partial removing of CH_2Cl_2 with a rotary evaporator. The precipitate was filtered off and dried *in vacuo*. Yield: 32.0 mg (21.7%). ^{1}H NMR (chloroform-*d*): δ (ppm) 7.84 (2H, AA'XX'm, J(AX) = 8.1 Hz, J(AA') = 1.8 Hz, J(AX') = 0.5 Hz, benzonitrile-*2* (A)), 7.83 (2H, AA'XX'm, J(A'X') = 7.7 Hz, J(AA') = 1.8 Hz, J(XA') = 0.5 Hz, benzonitrile-*6* (A')), 7.64 (2H, AA'XX'm, J(A'X') = 7.7 Hz, J(XX') = 1.8 Hz, J(AX') = 0.5 Hz, benzonitrile-*5* (X')), 7.64 (2H, AA'XX'm, J(AX) = 8.1 Hz, J(XX') = 1.8 Hz, J(XA') = 0.5 Hz, benzonitrile-*3* (X)), 7.63 (2H, s, phen-*5*), 7.45 (2H, s, phen-*3*), 3.02 (6H, s, phen-CH_3).

*2,9-dimethyl-4,7-bis(4-nitrophenyl)-1,10-phenanthroline (**dmp-NO$_2$ph**)* To a mixture of **dmp-Cl** (100 mg, 0.36 mmol), (4-nitrophenyl)boronic acid (150.2 mg, 0.89 mmol), and DMA (7 ml), an aqueous solution (1.8 ml) containing Na_2CO_3 (330 mg, 3.1 mmol) was added and degassed by N_2 bubbling. Then, $Pd(PPh_3)_4$ (16.6 mg, 0.0144 mmol) and EtOH (0.89 ml) were added and the solution was refluxed for one night under a N_2 atmosphere. After cooling to r.t., a 0.1 M NaOH aqueous solution (10 ml) was added to the reaction mixture and the product was extracted using CH_2Cl_2 and washed with water several times. The collected CH_2Cl_2 phase was dried with Na_2SO_4, and the solvent was removed with a rotary evaporator. Column chromatography on alumina (2.5 cm × 15 cm; aluminum oxide 90 standard (Merck)) with CH_2Cl_2 provided a solution containing **dmp-NO$_2$ph**. The product was purified further by reprecipitation, whereby a yellow powder precipitated from a saturated CH_2Cl_2–EtOH (9:1 v/v) solution *via* the partial removing of CH_2Cl_2 with a rotary evaporator. The precipitate was filtered off and dried *in vacuo*. Yield: 51.8 mg (31.9%). ^{1}H NMR (chloroform-*d*): δ (ppm) 8.41 (2H, AA'XX'm, *J*(AX) = 8.8 Hz, *J*(AA') = 2.4 Hz, *J*(AX') = 0.5 Hz, NO$_2$ph-*3* (A)), 8.41 (2H, AA'XX'm, *J*(A'X') = 8.8 Hz, *J*(AA') = 2.4 Hz, *J*(XA') = 0.5 Hz, NO$_2$ph-*5* (A')), 7.71 (2H, AA'XX'm, *J*(AX) = 8.8 Hz, *J*(XX') = 2.4 Hz, *J*(XA') = 0.5 Hz, NO$_2$ph-*2* (X)), 7.74 (2H, AA'XX'm, *J*(A'X') = 8.8 Hz, *J*(XX') = 2.4 Hz, *J*(B'A) = 0.5 Hz, NO$_2$ph-*6* (X')), 7.64 (2H, s, phen-*5*), 7.49 (2H, s, phen-*3*), 3.04 (6H, s, phen-C*H*$_3$).

4,7-di(furan-3-yl)-2,9-dimethyl-1,10-phenanthroline (dmp-3fu) This compound was synthesized according to the literature method[S10] with the following modifications. *n*-butanol (10 ml) was added to a mixture of **dmp-Cl** (150 mg, 0.54 mmol), 3-furanylboronic acid (145.5 mg, 1.30 mmol), Xphos (2-dicyclohexylphosphino-2',4',6'-triisopropylbiphenyl; 24.6 mg, 0.052 mmol), and $Pd(OAc)_2$ (9.7 mg,

0.043 mmol), and the mixture was degassed by N_2 bubbling. After stirring at r.t. for 15 min, a degassed aqueous solution of NaOH (147.2 mg in 3 ml water) was added, and the mixture was stirred further at r.t. for 4 h. After adding water, the product was extracted with CH_2Cl_2 and washed with water several times. Column chromatography on alumina (2.0 cm × 9 cm; aluminum oxide 90 standard (Merck)) with CH_2Cl_2 provided a solution containing **dmp-3fu**. The product was purified further by reprecipitation, whereby a white powder was precipitated from a saturated CH_2Cl_2 solution *via* the addition of *n*-hexane. The precipitate was filtered off and dried *in vacuo*. Although the product contained small amounts of impurities, as confirmed by ^{1}H NMR spectroscopy, this compound was reacted on without further purification. Yield: 116.8 mg (62.4%). ^{1}H NMR (chloroform-*d*): δ (ppm) 8.05 (2H, s, phen-*5*), 7.79 (2H, dd, $J(H_{fur2}H_{fur5})$ = 1.8 Hz, $J(H_{fur2}H_{fur4})$ = 1.1 Hz, furyl-*2*), 7.63 (2H, dd, $J(H_{fur5}H_{fur4})$ = 2.1 Hz, $J(H_{fur2}H_{fur5})$ = 1.8 Hz, furyl-*5*), 7.48 (2H, s, phen-*3*), 6.77 (2H, dd, $J(H_{fur5}H_{fur4})$ = 2.1 Hz, $J(H_{fur2}H_{fur4})$ = 1.1 Hz, furyl-*4*), 2.97 (6H, s, phen-CH_3).

*2,9-dimethyl-4,7-di(thiophen-3-yl)-1,10-phenanthroline **(dmp-3th)*** To a mixture of **dmp-Cl** (71.5 mg, 0.26 mmol), 3-thienylboronic acid (81.9 mg, 0.64 mmol), and DMA (7 ml), an aqueous solution (1.8 ml) containing Na_2CO_3 (330 mg, 3.1 mmol) was added and degassed by N_2 bubbling. Then, $Pd(PPh_3)_4$ (12.0 mg, 0.0104 mmol) and EtOH (0.89 ml) were added and the solution was refluxed for one night under a N_2 atmosphere. After cooling to r.t., a 0.1 M NaOH aqueous solution (10 ml) was added to the reaction mixture and the product was extracted using CH_2Cl_2 and washed with water several times. The collected CH_2Cl_2 phase was dried with Na_2SO_4 and the solvent was removed with a rotary evaporator. Column chromatography on alumina (2.5 cm × 7 cm; aluminum oxide 90 standard (Merck)) with CH_2Cl_2 provided a solution containing **dmp-3th**. The product was purified further by reprecipitation, whereby

a white powder precipitated from a saturated CH_2Cl_2 solution *via* the addition of *n*-hexane. The precipitate was filtered off and dried *in vacuo*. Yield: 63.6 mg (65.6%). ^{1}H NMR (chloroform-*d*): δ(ppm) 7.94 (2H, s, phen-*5*), 7.54 (2H, ABXm, *J*(AB) = 3.0 Hz, *J*(AX) = 1.5 Hz, thiophen-*2*), 7.52 (2H, ABXm, *J*(BX) = 5.0 Hz, *J*(AB) = 3.0 Hz, thiophen-*5*), 7.50 (2H, s, phen-*3*), 7.36 (2H, ABXq, *J*(BX) = 5.0 Hz, *J*(AX) = 1.5 Hz, thiophen-*4*), 2.98 (6H, s, phen-C*H*$_3$).

4,7-bis(benzo[b]thiophen-3-yl)-2,9-dimethyl-1,10-phenanthroline ***(dmp-3Bzth)*** This compound was synthesized according to the literature method[S10] with the following modifications. *n*-butanol (10 ml) was added to a mixture of **dmp-Cl** (150 mg, 0.54 mmol), benzo[*b*]thien-3-ylboronic acid (231.4 mg, 1.30 mmol), Xphos (2-dicyclohexylphosphino-2',4',6'-triisopropylbiphenyl; 24.6 mg, 0.052 mmol), and $Pd(OAc)_2$ (9.7 mg, 0.043 mmol), and the mixture was degassed by N_2 bubbling. After stirring at r.t. for 15 min, a degassed aqueous solution of NaOH (147.2 mg in 3 ml water) was added, and the mixture was stirred at r.t. for 4 h. After adding water, the product was extracted with CH_2Cl_2 and washed with water several times. Column chromatography on alumina (2.0 cm × 8.5 cm; aluminum oxide 90 standard (Merck)) with CH_2Cl_2 provided a solution containing **dmp-3Bzth**. The product was purified further by reprecipitation, whereby a white powder precipitated from a saturated CH_2Cl_2-EtOH (9:1 v/v) solution *via* the partial removing of CH_2Cl_2 with a rotary evaporator. The precipitate was filtered off and dried *in vacuo*. Yield: 256.7 mg (100%). ^{1}H NMR (chloroform-*d*): δ(ppm) 7.94 (2H, ABCXm, *J*(BX) = 7.8 Hz, *J*(AX) = *J*(CX) = 1.0 Hz, Benzothiophen-*7* (X)), 7.57 (2H, s, phen-*3*), 7.56 (2H, s, phen-*5*), 7.51 (2H, br, benzothiophen-*2*), 7.40 (2H, ABCXm, *J*(CA) = 8.0 Hz, *J*(CB) = *J*(CX) = 1.0 Hz, Benzothiophen-*4* (C)), 7.38 (2H, ABCXm, *J*(BX) = 7.8 Hz, *J*(AB) = 7.2 Hz, *J*(CB) = 1.0 Hz,

Benzothiophen-*6* (B)), 7.30 (2H, ABCXm, *J*(CA) = 8.0 Hz, *J*(AB) = 7.2 Hz, *J*(AX) = 1.0 Hz, Benzothiophen-*5* (A)), 3.03 (6H, s, phen-CH_3).

2,9-dimethyl-4,7-di(thiophen-2-yl)-1,10-phenanthroline ***(dmp-2th)*** To a mixture of **dmp-Cl** (100 mg, 0.36 mmol), 2-thienylboronic acid (81.9 mg, 0.64 mmol), and DMA (7 ml), an aqueous solution (1.8 ml) containing Na_2CO_3 (330 mg, 3.1 mmol) was added and degassed by N_2 bubbling. Then, $Pd(PPh_3)_4$ (16.6 mg, 0.0144 mmol) and EtOH (0.89 ml) were added and the solution was refluxed for one night under a N_2 atmosphere. After cooling to r.t., a 0.1 M NaOH aqueous solution (10 ml) was added to the reaction mixture and the product was extracted using CH_2Cl_2 and washed with water several times. The collected CH_2Cl_2 phase was dried with Na_2SO_4, and the solvent was removed with a rotary evaporator. Column chromatography three times on alumina (3.0 cm × 8 cm; aluminum oxide 90 standard (Merck)) with CH_2Cl_2 provided a solution containing **dmp-2th**. The product was recovered as a white powder by the evaporation of the solvent and dried *in vacuo*. Yield: 38.9 mg (29.0%). ^1H NMR (chloroform-*d*): δ (ppm) 8.18 (2H, s, phen-*5*), 7.57 (2H, s, phen-*3*), 7.53 (2H, dd, $J(H_{thi5}H_{thi4})$ = 5.3 Hz, $J(H_{thi5}H_{thi3})$ = 1.2 Hz, thiophen-*5*), 7.39 (2H, dd, $J(H_{thi4}H_{thi3})$ = 3.5 Hz, $J(H_{thi5}H_{thi3})$ = 1.2 Hz, thiophen-*3*), 7.23 (2H, dd, $J(H_{thi5}H_{thi4})$ = 5.3 Hz, $J(H_{thi4}H_{thi3})$ = 3.5 Hz, thiophen-*4*), 2.98 (6H, s, phen-CH_3).

4,7-bis(benzo[b]thiophen-2-yl)-2,9-dimethyl-1,10-phenanthroline ***(dmp-2Bzth)*** This compound was synthesized according to the literature method[S10] with the following modifications. *n*-butanol (8 ml) was added to a mixture of **dmp-Cl** (100 mg, 0.36 mmol), benzo[*b*]thien-2-ylboronic acid (153.8 mg, 0.86 mmol), Xphos (2-dicyclohexylphosphino-2',4',6'-triisopropylbiphenyl; 16.5 mg, 0.0346 mmol), and

$Pd(OAc)_2$ (6.5 mg, 0.0288 mmol), and the mixture was degassed by N_2 bubbling. After stirring at r.t. for 15 min, a degassed aqueous solution of NaOH (174 mg in 2 ml water) was added, and the mixture was stirred at r.t. for 4 h. After addiing water, the product was extracted with CH_2Cl_2 and washed with water several times. Column chromatography on alumina (3.0 cm × 14 cm; aluminum oxide 90 standard (Merck)) with CH_2Cl_2 provided a solution containing **dmp-2Bzth**. The product was recovered as a white powder by the evaporation of the solvent and dried *in vacuo*. Yield: 164.9 mg (87.6%). ^{1}H NMR (chloroform-*d*): δ(ppm) 8.25 (2H, s, phen-*5*), 7.93 (2H, AA'MXX'm, *J*(A'X) = 7.9 Hz, *J*(AX) = 1.1 Hz, *J*(X'X) = 0.8 Hz, Benzothiophen-*7* (X)), 7.89 (2H, AA'MXX'm, *J*(X'A) = 8.0 Hz, *J*(X'A') = 0.9 Hz, *J*(X'X) = 0.8 Hz, *J*(MX') = 0.6 Hz, Benzothiophen-*4* (X')), 7.67 (2H, s, phen-*3*), 7.60 (2H, AA'MXX'd, *J*(MX') = 0.6 Hz, Benzothiophen-*3*), 7.45 (2H, AA'MXX'm, *J*(X'A) = 8.0 Hz, *J*(AA') = 7.4 Hz, *J*(AX) = 1.1 Hz, Benzothiophen-*5* (A)), 7.43 (2H, AA'MXX'm, *J*(A'X) = 7.9 Hz, *J*(AA') = 7.4 Hz, *J*(X'A') = 0.9 Hz, Benzothiophen-*6* (A')), 3.03 (6H, s, phen-C*H*$_3$).

*4,7-di(benzofuran-2-yl)-2,9-dimethyl-1,10-phenanthroline (**dmp-2Bzfu**)* This compound was synthesized according to the literature method[S10] with the following modifications. 1-propanol (10 ml) was added to a mixture of **dmp-Cl** (150 mg, 0.54 mmol), benzofuran-2-ylboronic acid (210.5 mg, 1.30 mmol), Xphos (2-dicyclohexylphosphino-2',4',6'-triisopropylbiphenyl; 24.6 mg, 0.052 mmol), and $Pd(OAc)_2$ (9.7 mg, 0.043 mmol), and the mixture was degassed by N_2 bubbling. After stirring at r.t. for 15 min, a degassed aqueous solution of NaOH (196 mg in 3 ml water) was added, and the mixture was stirred at r.t. for 4 h. After adding water, the product was extracted with CH_2Cl_2 and washed with water several times. In the water phase, a pale-yellow powder precipitated, which was also recovered by filtration and combined with the solid obtained from the CH_2Cl_2 phase. Column chromatography

on alumina (2.0 cm × 7 cm; aluminum oxide 90 standard (Merck)) with CH_2Cl_2 provided a solution containing **dmp-2Bzfu**. The product was purified further by reprecipitation, whereby a pale yellow powder precipitated from a saturated CH_2Cl_2–EtOH (9:1 v/v) solution *via* the partial removing of CH_2Cl_2 with a rotary evaporator. The precipitate was filtered off and dried *in vacuo*. Yield: 163.0 mg (68.5%). ^{1}H NMR (chloroform-*d*): δ (ppm) 8.54 (2H, s, phen-*5*), 7.92 (2H, s, phen-*3*), 7.73 (2H, AA'BXX'm, *J*(A'X) = 8.2 Hz, *J*(AX) = 1.1 Hz, *J*(X'X) = 0.8 Hz, Benzofuran-*7* (X)), 7.66 (2H, AA'BXX'm, *J*(X'A) = 8.1 Hz, *J*(X'A') = 0.9 Hz, *J*(BX') = *J*(X'X) = 0.8 Hz, Benzofuran-*4* (X')), 7.42 (2H, AA'BXX'm, *J*(X'A) = 8.1 Hz, *J*(AA') = 7.1 Hz, *J*(AX) = 1.1 Hz, Benzofuran-*5* (A)), 7.36 (2H, AA'BXX'd, *J*(BX') = 0.9 Hz, Benzofuran-*3* (B)), 7.35 (2H, AA'BXX'm, *J*(A'X) = 8.2 Hz, *J*(AA') = 7.1 Hz, *J*(X'A') = 0.8 Hz, Benzofuran-*6* (A')), 3.04 (6H, s, phen-CH_3).

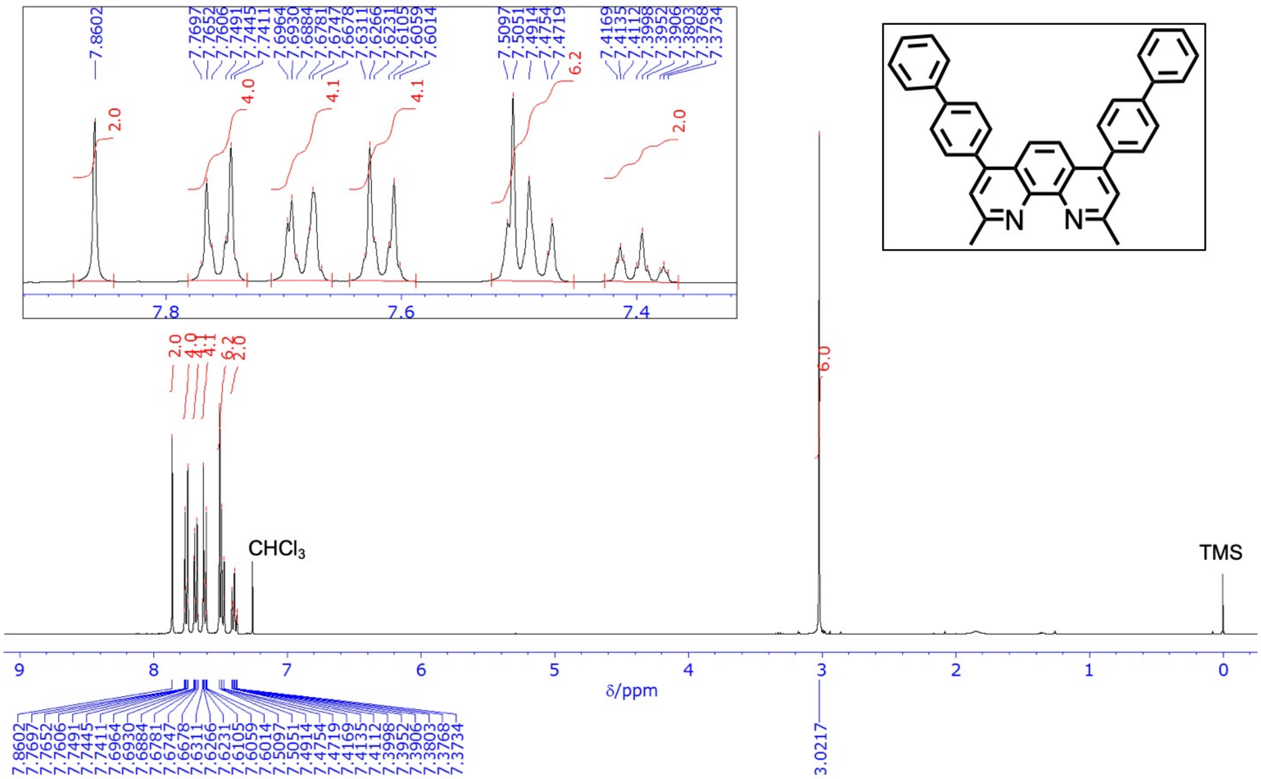

Figure S21. ^{1}H NMR spectrum (400 MHz, chloroform-*d*) of **dmp-Bph**.

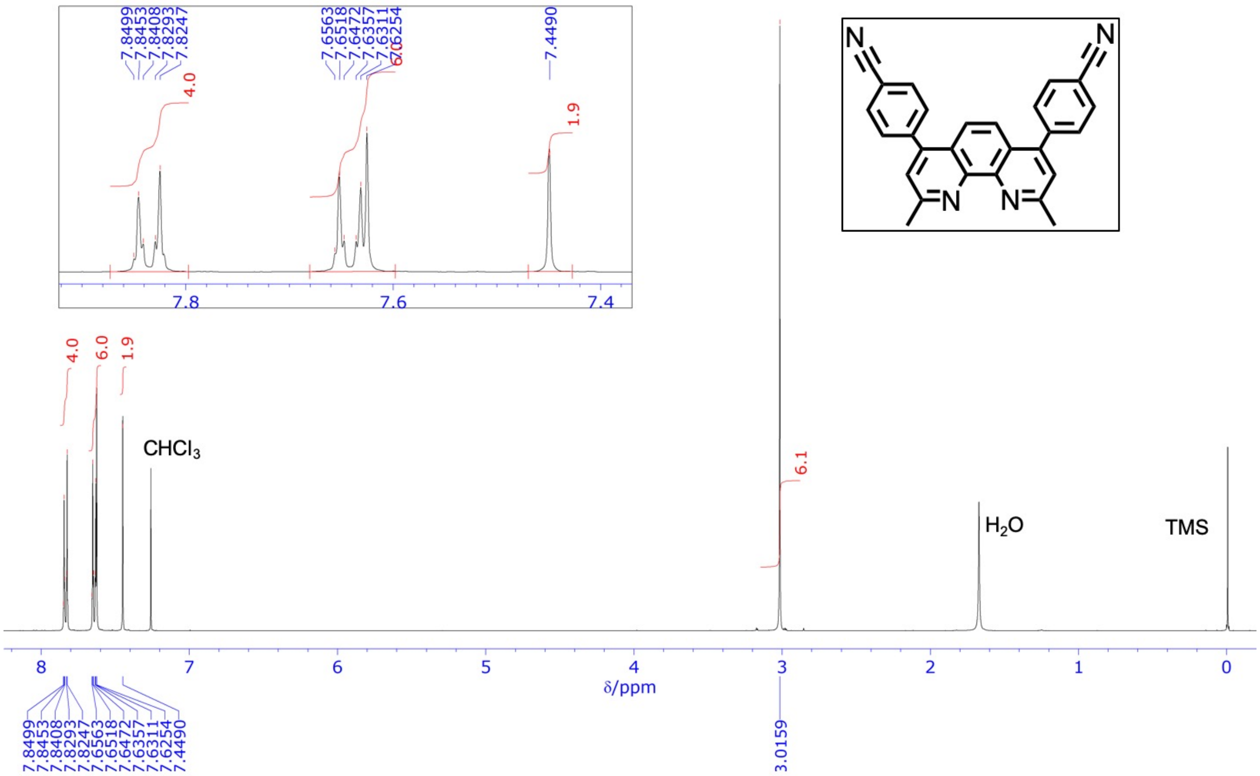

Figure S22. ^{1}H NMR spectrum (400 MHz, chloroform-*d*) of **dmp-NCph**.

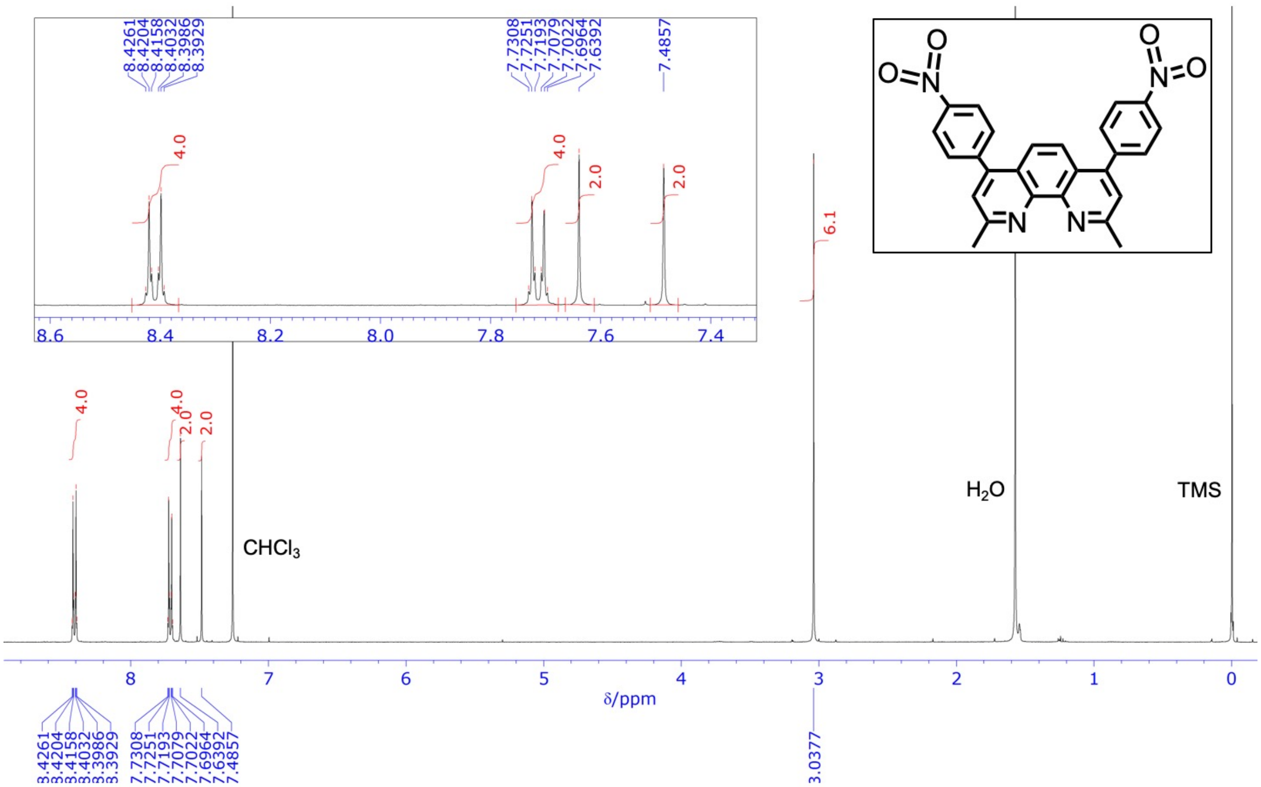

Figure S23. ^{1}H NMR spectrum (400 MHz, chloroform-*d*) of **dmp-NO$_2$ph**.

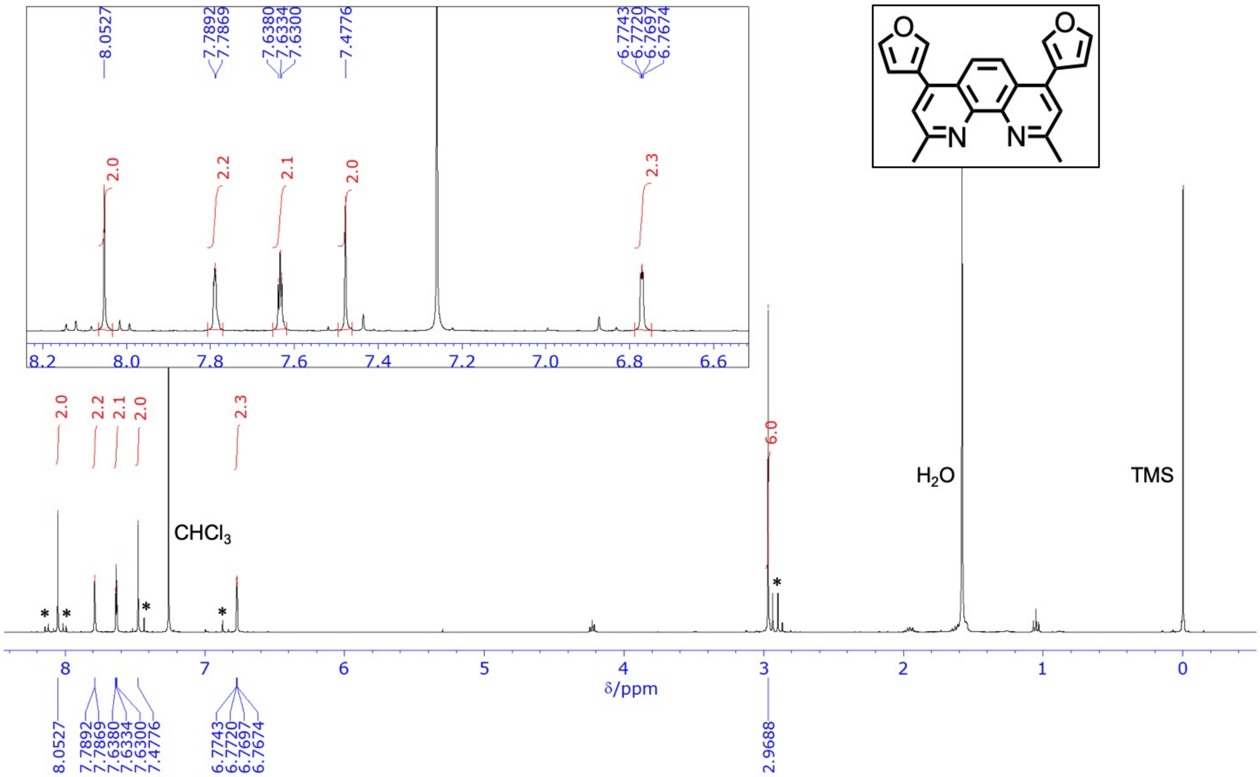

Figure S24. ^{1}H NMR spectrum (400 MHz, chloroform-*d*) of **dmp-3fu**.

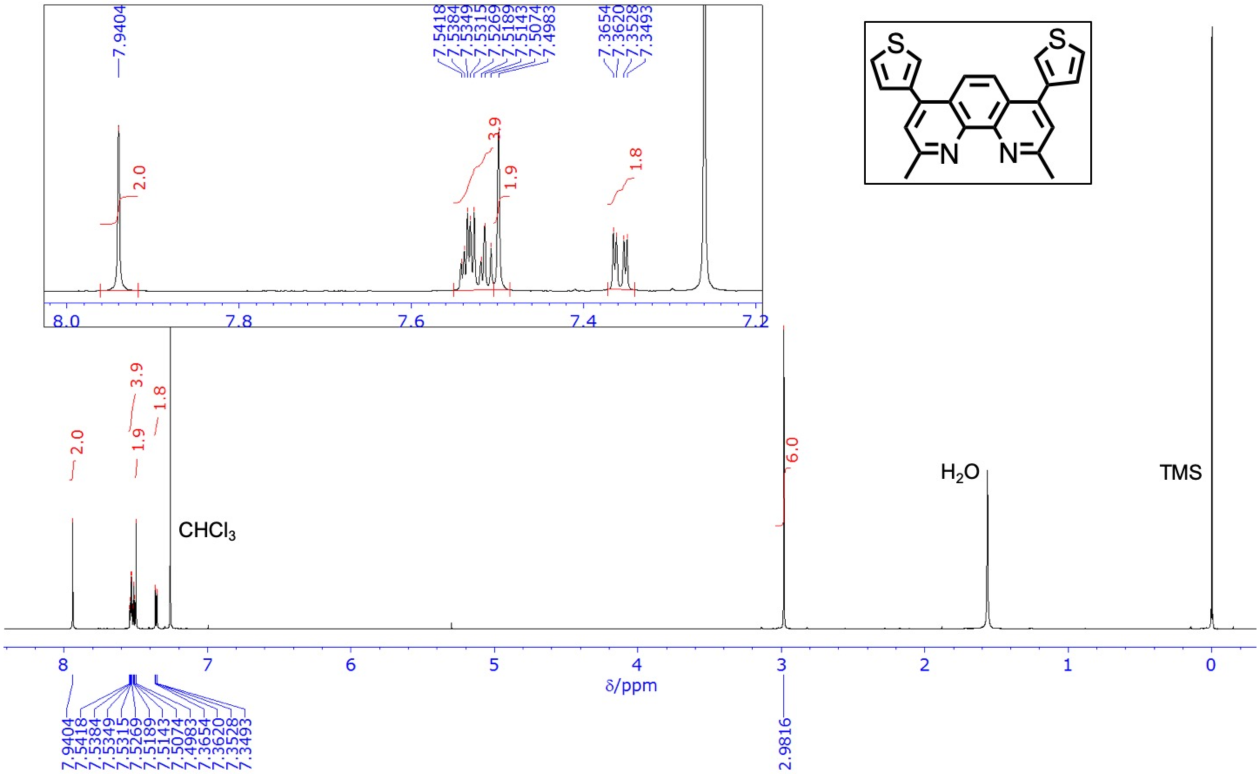

Figure S25. ^{1}H NMR spectrum (400 MHz, chloroform-*d*) of **dmp-3th**.

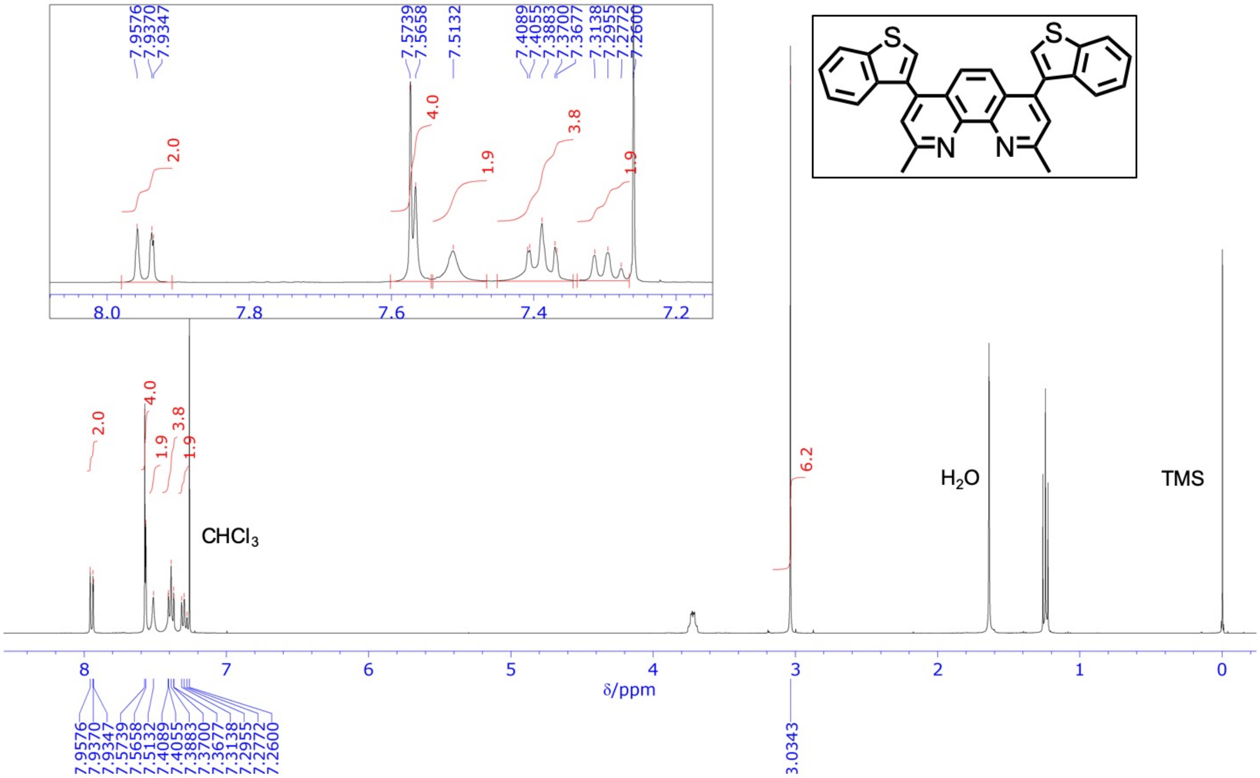

Figure S26. ^{1}H NMR spectrum (400 MHz, chloroform-*d*) of **dmp-3Bzth**.

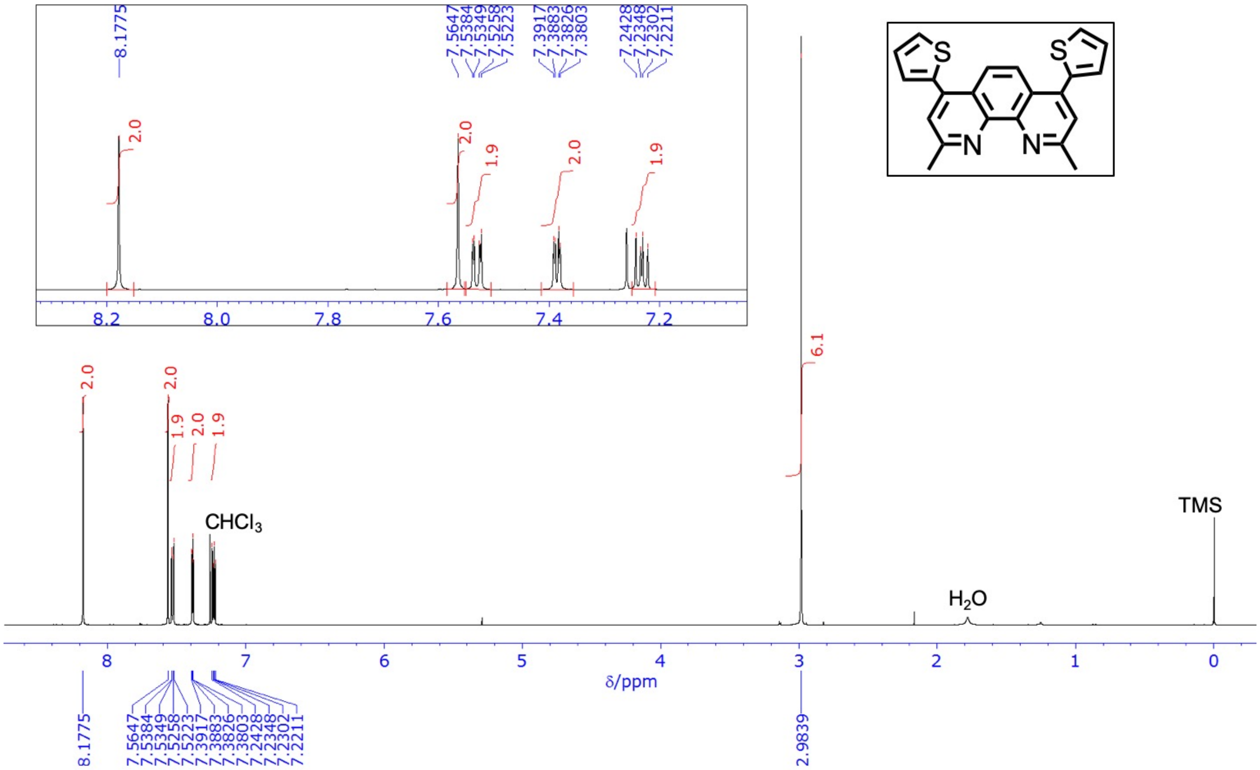

Figure S27. ^{1}H NMR spectrum (400 MHz, chloroform-*d*) of **dmp-2th**.

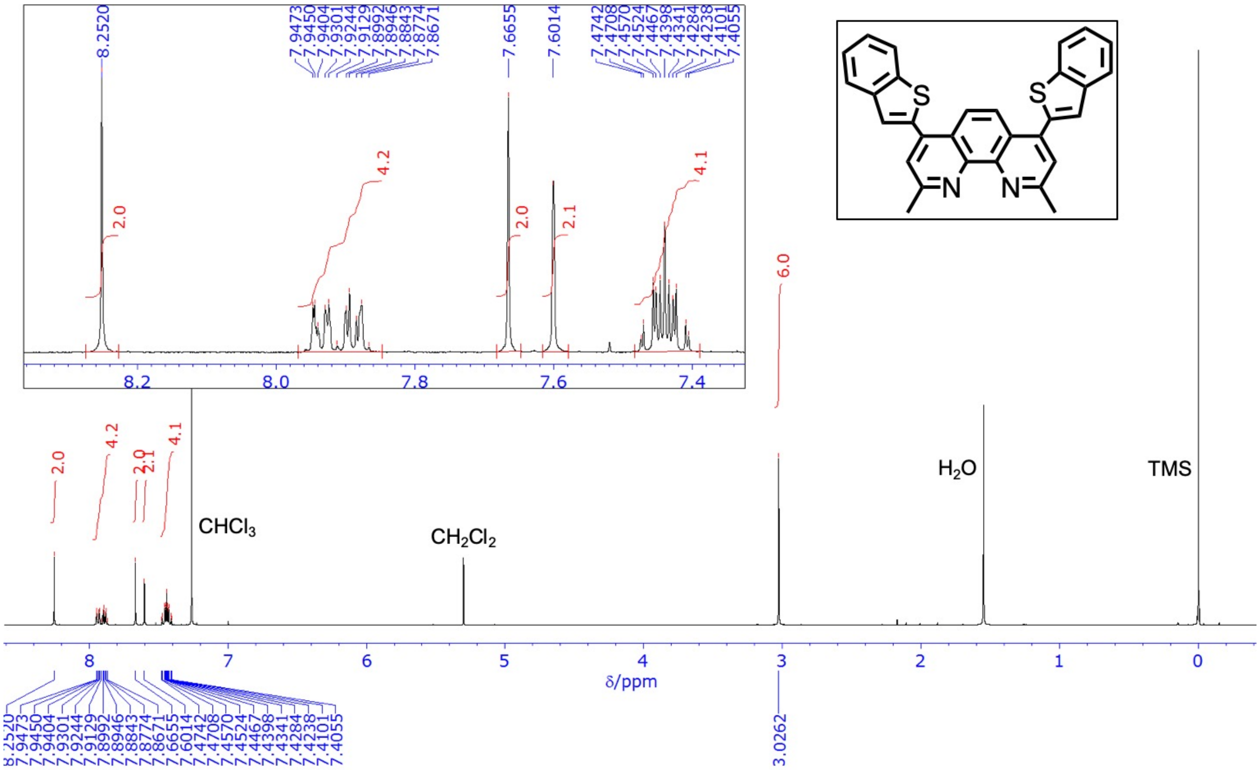

Figure S28. ^{1}H NMR spectrum (400 MHz, chloroform-*d*) of **dmp-2Bzth**.

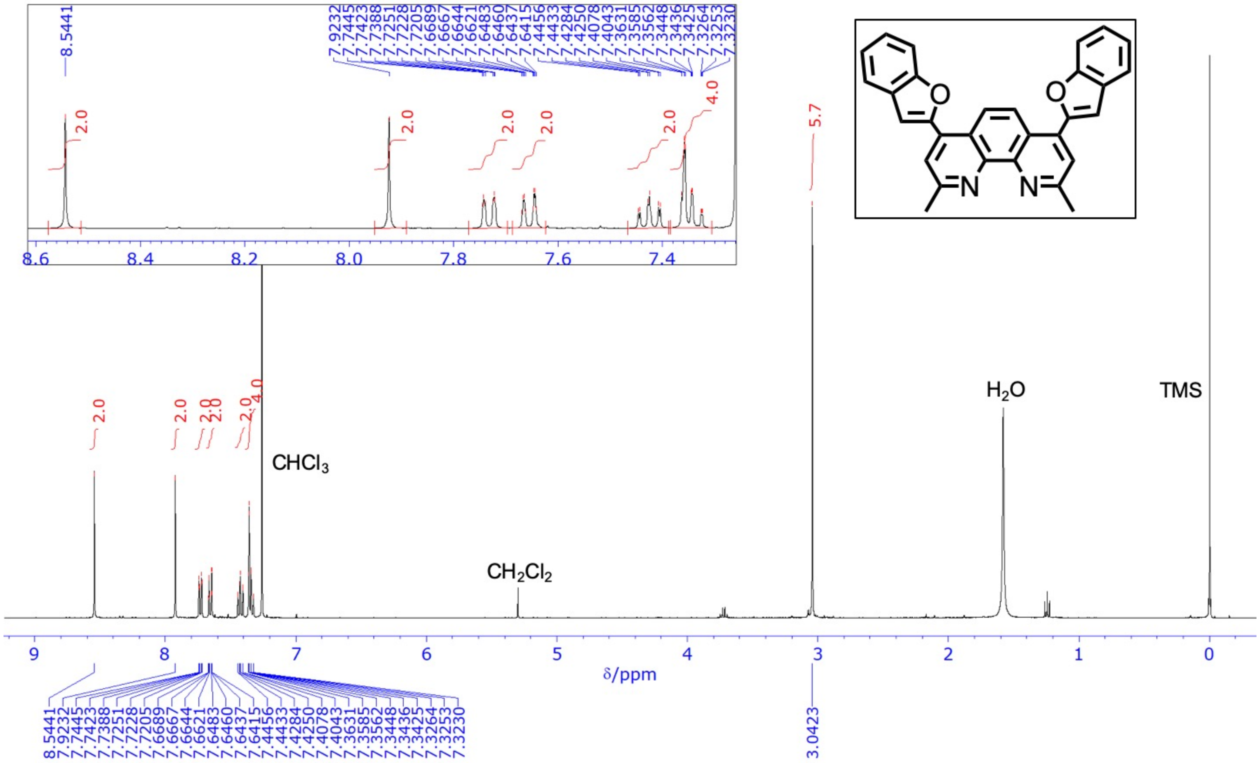

Figure S29. ^{1}H NMR spectrum (400 MHz, chloroform-*d*) of **dmp-2Bzfu**.

dmp-Bph

nucleus	n	δ / ppm	multiplicity	J / Hz							
H_5	2	7.8605	s								
A1	2	7.7545	ddd	J(A1X1)	8.5	J(A1A1')	2.2	J(A1X1')	0.5		
A1'	2	7.7534	ddd	J(A1'X1')	8.5	J(A1A1')	2.2	J(X1A1')	0.5		
A2	2	7.6850	dddd	J(A2M2)	8.0	J(A2X2)	1.2	J(A2A2')	1.0	J(A2M2')	0.5
A2'	2	7.6830	dddd	J(A2'M2')	8.6	J(X2A2')	1.2	J(A2A2')	1.0	J(M2A2')	0.5
X1'	2	7.6179	ddd	J(A1'X1')	8.5	J(X1X1')	2.2	J(A1X1')	0.5		
X1	2	7.6173	ddd	J(A1X1)	8.5	J(X1X1')	2.2	J(X1A1')	0.5		
H_3	2	7.5054	s								
M2'	2	7.4911	dddd	J(A2'M2')	8.6	J(X2M2')	7.5	J(M2M2')	2.0	J(A2M2')	0.5
M2	2	7.4906	dddd	J(A2M2)	8.0	J(M2X2)	7.5	J(M2M2')	2.0	J(M2A2')	0.5
X2	2	7.3975	dddd	J(X2M2')	7.5	J(M2X2)	7.5	J(A2X2)	1.2	J(X2A2')	1.2

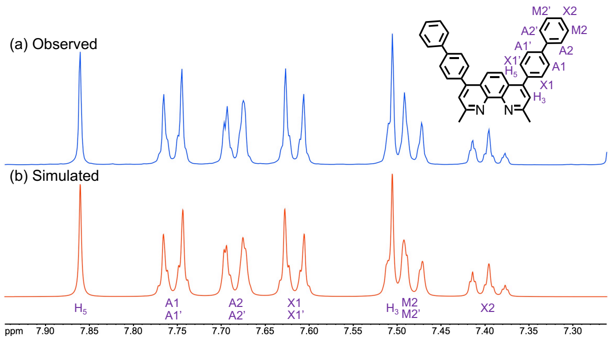

dmp-NCph

nucleus	n	δ / ppm	multiplicity	J / Hz					
A	2	7.8352	ddd	J(AX)	8.1	J(AA')	1.8	J(AX')	0.5
A'	2	7.8343	ddd	J(A'X')	7.7	J(AA')	1.8	J(XA')	0.5
X'	2	7.6423	ddd	J(A'X')	7.7	J(XX')	1.8	J(AX')	0.5
X	2	7.6421	ddd	J(AX)	8.1	J(XX')	1.8	J(XA')	0.5
H_5	2	7.6257	s						
H_3	2	7.4498	s						

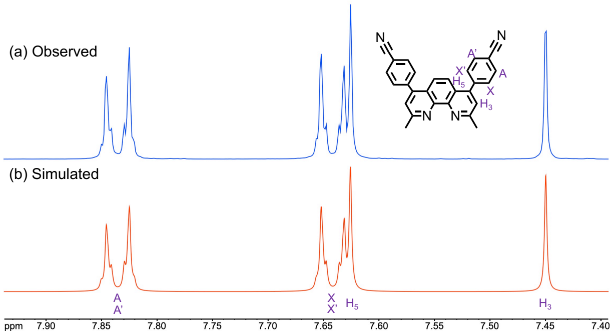

dmp-NO_2ph

nucleus	n	δ / ppm	multiplicity	J / Hz					
A	2	8.4092	ddd	J(AX)	8.8	J(AA')	2.4	J(AX')	0.5
A'	2	8.4092	ddd	J(A'X')	8.8	J(AA')	2.4	J(XA')	0.5
X	2	7.7143	ddd	J(AX)	8.8	J(XX')	2.4	J(XA')	0.5
X'	2	7.7139	ddd	J(A'X')	8.8	J(XX')	2.4	J(AX')	0.5
H_5	2	7.6394	s						
H_3	2	7.4855	s						

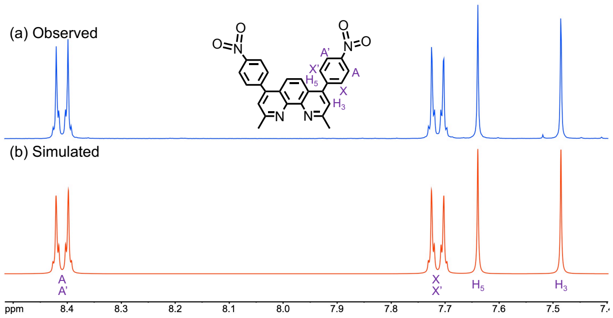

dmp-3fu

nucleus	n	δ / ppm	multiplicity	J / Hz			
H_5	2	8.0525	s				
H_{fur2}	2	7.7898	dd	$J(H_{fur2}H_{fur5})$	1.8	$J(H_{fur2}H_{fur4})$	1.1
H_{fur5}	2	7.6342	dd	$J(H_{fur5}H_{fur4})$	2.1	$J(H_{fur2}H_{fur5})$	1.8
H_3	2	7.4782	s				
H_{fur4}	2	6.7710	dd	$J(H_{fur5}H_{fur4})$	2.1	$J(H_{fur2}H_{fur4})$	1.1

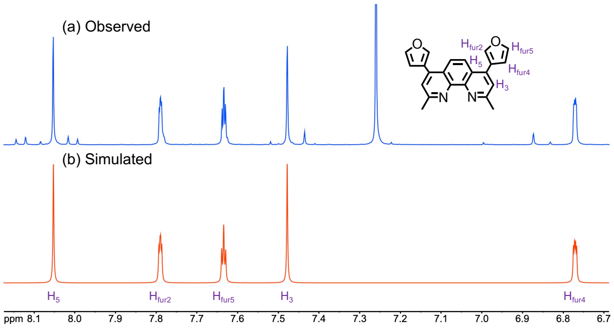

dmp-3th

nucleus	n	δ / ppm	multiplicity	J / Hz			
H_5	2	7.9402	s				
A	2	7.5361	dd	J(AB)	3.0	J(AX)	1.5
B	2	7.5183	dd	J(BX)	5.0	J(AB)	3.0
H_3	2	7.4988	s				
X	2	7.3580	dd	J(BX)	5.0	J(AX)	1.5

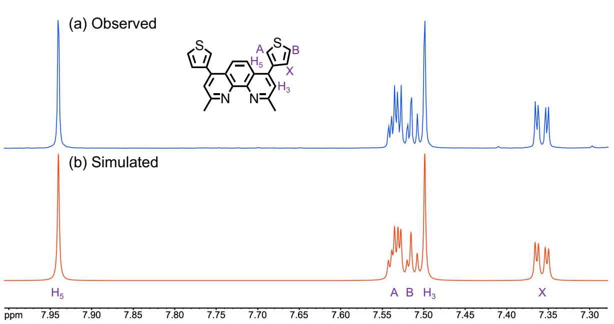

dmp-3Bzth

nucleus	n	δ / ppm	multiplicity	J / Hz					
X	2	7.9447	ddd	J(BX)	7.8	J(AX)	1.0	J(CX)	1.0
H_3	2	7.5703	s						
H_5	2	7.5601	s						
H_{Bzthi2}	2	7.5102	s						
C	2	7.3957	ddd	J(CA)	8.0	J(CB)	1.0	J(CX)	1.0
B	2	7.3844	ddd	J(BX)	7.8	J(AB)	7.2	J(CB)	1.0
A	2	7.2963	ddd	J(CA)	8.0	J(AB)	7.2	J(AX)	1.0

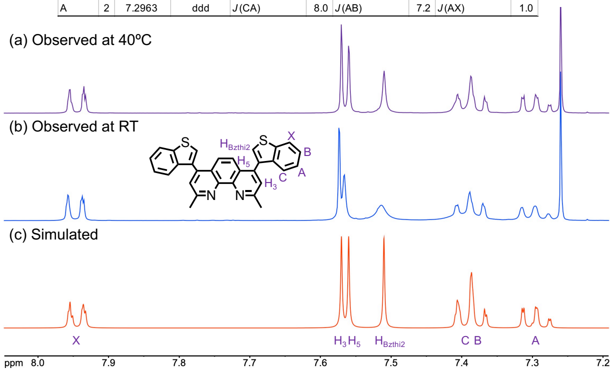

Figure S30. Peak analysis of the ^{1}H NMR spectra (400 MHz, chloroform-*d*) of **dmp-Bph**, **dmp-NCph**, **dmp-NO₂ph**, **dmp-3fu**, **dmp-3th**, and **dmp-3Bzth**.

dmp-2th

nucleus	n	δ / ppm	multiplicity	J / Hz			
H_5	2	8.1782	s				
H_3	2	7.5652	s				
H_{thi5}	2	7.5305	dd	$J(H_{thi5}H_{thi4})$	5.3	$J(H_{thi5}H_{thi3})$	1.2
H_{thi3}	2	7.3859	dd	$J(H_{thi4}H_{thi3})$	3.5	$J(H_{thi5}H_{thi3})$	1.2
H_{thi4}	2	7.2330	dd	$J(H_{thi5}H_{thi4})$	5.3	$J(H_{thi4}H_{thi3})$	3.5

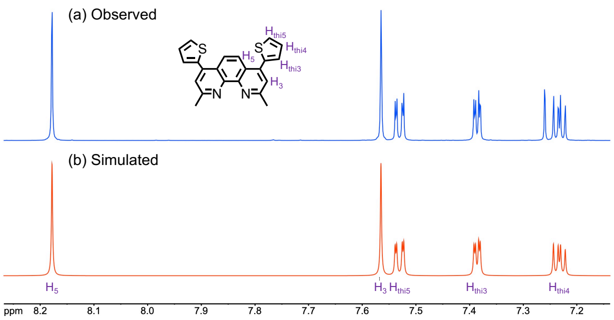

dmp-2Bzth

nucleus	n	δ / ppm	multiplicity	J / Hz							
H_5	2	8.2513	s								
X	2	7.9349	ddd	J(A'X)	7.9	J(AX)	1.1	J(X'X)	0.8		
X'	2	7.8876	dddd	J(X'A)	8.0	J(X'A')	0.9	J(X'X)	0.8	J(MX')	0.6
H_3	2	7.6652	s								
M	2	7.6002	d	J(MX')	0.6						
A	2	7.4509	ddd	J(X'A)	8.0	J(AA')	7.4	J(AX)	1.1		
A'	2	7.4295	ddd	J(A'X)	7.9	J(AA')	7.4	J(X'A')	0.9		

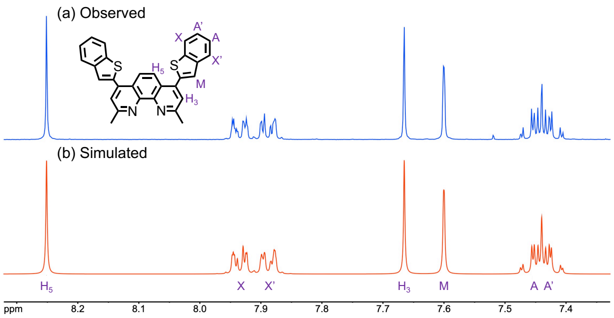

dmp-2Bzfu

nucleus	n	δ / ppm	multiplicity	J / Hz							
H_5	2	8.5440	s								
H_3	2	7.9241	s								
X	2	7.7318	ddd	J(A'X)	8.2	J(AX)	1.1	J(X'X)	0.8		
X'	2	7.6550	dddd	J(X'A)	8.1	J(X'A')	0.9	J(BX')	0.8	J(X'X)	0.8
A	2	7.4249	ddd	J(X'A)	8.1	J(AA')	7.1	J(AX)	1.1		
B	2	7.3583	d	J(BX')	0.9						
A'	2	7.3451	ddd	J(A'X)	8.2	J(AA')	7.1	J(X'A')	0.8		

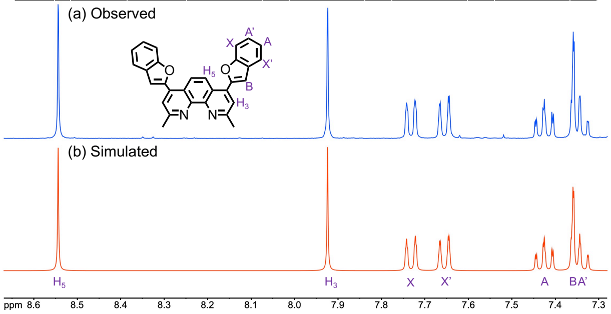

Figure S31. Peak analysis of the ^1H NMR spectra (400 MHz, chloroform-*d*) of **dmp-2th**, **dmp-2Bzth**, and **dmp-2Bzfu**.

*Cu^I(dmp-Bph)(DPEphos)(PF_6) (**Cu(Bph)**)* This complex was synthesized according to a literature method.[S3] [Cu^I(CH_3CN)$_4$](PF_6) (68.7 mg, 0.134 mmol) and DPEphos (72.2 mg, 0.134 mmol) were dissolved in CH_2Cl_2 (20 ml). The solution was stirred at r.t. for 0.5 h, and a **dmp-Bph** (68.7 mg, 0.134 mmol) powder was added to it. After additional stirring at r.t. for 2 h, the solvent was removed with a rotary evaporator. Column chromatography on silica gel (2.0 cm × 7.0 cm; silica gel 60 (Kanto)) using CH_2Cl_2–Et_2O (1:1 v/v) provided a solution containing **Cu(Bph)**. The product was purified further by

reprecipitation from a CH_3OH-Et_2O–*n*-hexane. The resulting yellow powder was filtered off and dried *in vacuo*. Yield: 147.6 mg (87.4%). ^1H NMR (chloroform-*d*): δ (ppm) 7.94 (2H, s, phen-*5*), 7.81 (2H, A1A1'X1X1'm, *J*(A1X1) = 8.1 Hz, *J*(A1A1') = 1.8 Hz, *J*(A1X1') = 0.9 Hz, bph-*2* (A1)), 7.81 (2H, A1A1'X1X1'm, *J*(A1'X1') = 7.1 Hz, *J*(A1A1') = 1.8 Hz, *J*(X1A1') = 0.9 Hz, bph-*6* (A1')), 7.69 (2H, A2A2'M2M2'X2m, *J*(A2M2) = 7.6 Hz, *J*(A2'A2) = 1.6 Hz, *J*(X2A2) = 1.2 Hz, *J*(M2'A2) = 0.4 Hz, bph-*2′* (A2)), 7.68 (2H, A2A2'M2M2'X2m, *J*(A2'M2') = 8.3 Hz, *J*(A2'X2) = 1.6 Hz, *J*(A2'A2) = 1.1 Hz, *J*(A2'M2) = 0.6 Hz, bph-*6′* (A2')), 7.61 (2H, A1A1'X1X1'm, *J*(A1X1) = 8.1 Hz, *J*(X1X1') = 1.5 Hz, *J*(X1A1') = 0.9 Hz, bph-*3* (X1)), 7.61 (2H, A1A1'X1X1'm, *J*(A1'X1') = 8.1 Hz, *J*(X1X1') = 1.5 Hz, *J*(A1X1') = 0.9 Hz, bph-*5* (X1')), 7.57 (2H, s, phen-*3*), 7.50 (2H, A2A2'M2M2'X2m, *J*(A2M2) = *J*(X2M2) = 7.6 Hz, *J*(M2'M2) = 0.9 Hz, *J*(A2'M2) = 0.6 Hz, bph-*3′* (M2)), 7.50 (2H, A2A2'M2M2'X2m, *J*(A2'M2') = 8.3 Hz, *J*(M2'X2) = 7.4 Hz, *J*(M2'M2) = 0.9 Hz, *J*(M2'A2) = 0.4 Hz, bph-*5′* (M2')), 7.42 (2H, A2A2'M2M2'X2m, *J*(X2M2) = 7.6 Hz, *J*(M2'X2) = 7.4 Hz, *J*(A2'X2) = 1.2 Hz, *J*(X2A2') = 1.1 Hz, bph-*4′* (X2)), 7.4-6.9 (28H, m, DPEphos), 2.54 (6H, s, phen-CH_3). Elemental Anal. Calcd (%) for $C_{74}H_{56}CuF_6N_2OP_3$: C, 70.56; H, 4.48; N, 2.22. Found: C, 70.72; H, 4.50; N, 2.23.

Cu^I(dmp-NCph)(DPEphos)(PF_6) ***(Cu(NCph))*** This complex was synthesized according to a literature method.[S3] [Cu^I(CH_3CN)$_4$](PF_6) (22.7 mg, 0.061 mmol) and DPEphos (32.9 mg, 0.061 mmol) were dissolved in CH_2Cl_2 (20 ml). The solution was stirred at r.t. for 2 h, and **dmp-NCph** (25.0 mg, 0.061 mmol) was added to it. After additional stirring at r.t. for 2 h, the solvent was removed with a rotary evaporator. The product was purified by reprecipitation from a CH_3OH–CH_2Cl_2–*n*-hexane solution. The resulting yellow powder was filtered off and dried *in vacuo*. Yield: 69.5 mg (87.9 %). ^1H NMR (chloroform-*d*): δ (ppm) 7.87 (2H, AA'XX'm, *J*(AX) = 7.9 Hz, *J*(AA') = 1.8 Hz, *J*(AX') = 0.5 Hz,

benzonitrile-*2* (A)), 7.87 (2H, AA'XX'm, *J*(A'X') = 7.9 Hz, *J*(AA') = 1.8 Hz, *J*(XA') = 0.5 Hz, benzonitrile-*6* (A')), 7.72 (2H, s, phen-*5*), 7.68 (2H, AA'XX'm, *J*(AX) = 7.9 Hz, *J*(XX') = 1.8 Hz, *J*(XA') = 0.5 Hz, benzonitrile-*3* (X)), 7.68 (2H, AA'XX'm, *J*(A'X') = 7.9 Hz, *J*(XX') = 1.8 Hz, *J*(AX') = 0.5 Hz, benzonitrile-*5* (X')), 7.55 (2H, s, phen-*3*), 7.4-6.9 (28H, m, DPEphos), 2.55 (6H, s, phen-C*H*3). Anal. Calcd (%) for $C_{54}H_{46}CuF_6N_4OP_3$: C, 66.41; H, 4.01; N, 4.84. Found: C, 66.12; H, 3.97; N, 4.75.

Cu^I(dmp-NO$_2$ph)(DPEphos)(PF$_6$) ***(Cu(NO$_2$ph))*** This complex was synthesized according to a literature method.[S3] [Cu^I(CH_3CN)$_4$](PF_6) (20.9 mg, 0.056 mmol) and DPEphos (30.2 mg, 0.056 mmol) were dissolved in CH_2Cl_2 (20 ml). The solution was stirred at r.t. for 0.5 h, and **dmp-NO$_2$ph** (25.0 mg, 0.056 mmol) was added to it. After additional stirring at r.t. for 2 h, the solvent was removed with a rotary evaporator. The product was purified by reprecipitation from a CH_3OH–CH_2Cl_2–*n*-hexane solution. The resulting yellow powder was filtered off and dried *in vacuo*. Yield: 55.1 mg (84.9 %). ^{1}H NMR (chloroform-*d*): δ (ppm) 8.42 (2H, AA'XX'm, *J*(AX) = 8.4 Hz, *J*(AA') = 2.2 Hz, *J*(AX') = 0.5 Hz, NO_2ph-*3* (A)), 8.42 (2H, AA'XX'm, *J*(A'X') = 8.4 Hz, *J*(AA') = 2.2 Hz, *J*(XA') = 0.5 Hz, NO_2ph-*5* (A')), 7.74 (2H, AA'XX'm, *J*(AX) = 8.4 Hz, *J*(XX') = 2.2 Hz, *J*(XA') = 0.5 Hz, NO_2ph-*2* (X)), 7.74 (2H, AA'XX'm, *J*(A'X') = 8.4 Hz, *J*(XX') = 2.2 Hz, *J*(AX') = 0.5 Hz, NO_2ph-*6* (X')), 7.72 (2H, s, phen-*5*), 7.59 (2H, s, phen-*3*), 7.4-6.9 (28H, m, DPEphos), 2.57 (6H, s, phen-C*H*3). Anal. Calcd (%) for $C_{62}H_{46}CuF_6N_4O_5P_3$: C, 62.18; H, 3.87; N, 4.68. Found: C, 62.05; H, 3.84; N, 4.65.

Cu^I(dmp-3fu)(DPEphos)(PF$_6$) ***(Cu(3fu))*** This complex was synthesized according to a literature method.[S3] [Cu^I(CH_3CN)$_4$](PF_6) (50.0 mg, 0.134 mmol) and DPEphos (72.2 mg, 0.134 mmol) were dissolved in CH_2Cl_2 (20 ml). The solution was stirred at r.t. for 0.5 h, and **dmp-3fu** (45.6 mg, 0.134

mmol) was added to it. After additional stirring at r.t. for 2 h, the solvent was removed with a rotary evaporator. The product was purified by reprecipitation from a CH_3OH–Et_2O–*n*–hexane solution. Column chromatography on silica gel (2.0 cm × 10 cm; silica gel 60 (Kanto)) using CH_2Cl_2–Et_2O (1:1 v/v) provided a solution containing **Cu(3fu)**. The product was further purified by reprecipitation from a CH_3OH–Et_2O–*n*-hexane. The resulting yellow powder was filtered off and dried *in vacuo*. Yield: 90.2 mg (61.9%). ^{1}H NMR (chloroform-*d*): δ (ppm) 8.14 (2H, s, phen-*5*), 7.88 (2H, dd, $J(H_{fur2}H_{fur5})$ = 1.5 Hz, $J(H_{fur2}H_{fur4})$ = 0.9 Hz, furyl-*2*), 7.68 (2H, dd, $J(H_{fur5}H_{fur4})$ = 1.9 Hz, $J(H_{fur2}H_{fur5})$ = 1.5 Hz, furyl-*5*), 7.55 (2H, s, phen-*3*), 6.82 (2H, dd, $J(H_{fur5}H_{fur4})$ = 1.9 Hz, $J(H_{fur2}H_{fur4})$ = 0.9 Hz, furyl-*4*), 7.4-6.9 (28H, m, DPEphos), 2.48 (6H, s, phen-C*H*$_3$). Anal. Calcd (%) for $C_{58}H_{44}CuF_6N_2O_3P_3$: C, 64.06; H, 4.08; N, 2.58. Found: C, 63.69; H, 4.20; N, 2.39.

Cu^I(dmp-3th)(DPEphos)(PF$_6$) ***(Cu(3th))*** This complex was synthesized according to a literature method.[S3] $[Cu^I(CH_3CN)_4](PF_6)$ (25.0 mg, 0.067 mmol) and DPEphos (36.1 mg, 0.067 mmol) were dissolved in CH_2Cl_2 (20 ml). The solution was stirred at r.t. for 0.5 h, and **dmp-3th** (25.0 mg, 0.067 mmol) was added to it. After additional stirring at r.t. for 2 h, the solvent was removed with a rotary evaporator. The product was purified by reprecipitation from a CH_3OH–CH_2Cl_2–*n*-hexane solution. Column chromatography on silica gel (2.0 cm × 7.5 cm; silica gel 60 (Kanto)) using CH_2Cl_2-Et_2O (1:1 v/v) provided a solution containing **Cu(3th)**. The solvent was removed with a rotary evaporator and dried *in vacuo*, provided a yellow powder. Yield: 57.0 mg (76.0%). ^{1}H NMR (chloroform-*d*): δ (ppm) 8.03 (2H, s, phen-*5*), 7.64 (2H, dd, $J(H_{thi2}H_{thi5})$ = 2.9 Hz, $J(H_{thi2}H_{thi4})$ = 1.3 Hz, thiophen-*2*), 7.59 (2H, dd, $J(H_{thi5}H_{thi4})$ = 4.8 Hz, $J(H_{thi2}H_{thi5})$ = 2.9 Hz, thiophen-*5*), 7.56 (2H, s, phen-*3*), 7.37 (2H, dd, $J(H_{thi5}H_{thi4})$ = 4.8 Hz, $J(H_{thi2}H_{thi4})$ = 1.3 Hz, thiophen-*4*), 7.4-6.9 (28H, m, DPEphos), 2.50 (6H, s, phen-

CH_3). Anal. Calcd (%) for $C_{58}H_{44}CuF_6N_2OP_3S_2$: C, 62.22; H, 3.96; N, 2.50; S, 5.73. Found: C, 62.08; H, 4.07; N, 2.44; S, 5.74.

Cu^I(dmp-3Bzth)(DPEphos)(PF_6) ***(Cu(3Bzth))*** This complex was synthesized according to a literature method.[S3] [$Cu^I(CH_3CN)_4$](PF_6) (50.0 mg, 0.134 mmol) and DPEphos (72.2 mg, 0.134 mmol) were dissolved in CH_2Cl_2 (20 ml). The solution was stirred at r.t. for 0.5 h, and **dmp-3Bzth** (63.3 mg, 0.134 mmol) was added to it. After additional stirring at r.t. for 2 h, the solvent was removed with a rotary evaporator. The product was purified by reprecipitation from a CH_3OH–CH_2Cl_2–*n*-hexane solution. The resulting yellow powder was filtered off and dried *in vacuo*. Yield: 129.0 mg (78.9%). ^{1}H NMR (chloroform-*d*): δ (ppm) 7.98 (2H, ABCXm, *J*(CX) = 8.2 Hz, *J*(AX) = 1.0 Hz, *J*(BX) = 0.2 Hz, Benzothiophen-*7* (X)), 7.71 (2H, s, phen-*5*), 7.65 (2H, s, phen-*3*), 7.62 (2H, br, benzothiophen-*2*), 7.43 (2H, ABCXm, *J*(CX) = 8.2 Hz, *J*(BC) = 8.0 Hz, *J*(AC) = 0.6 Hz, Benzothiophen-*6* (C)), 7.35 (2H, ABCXm, *J*(BC) = 8.0 Hz, *J*(AB) = 7.6 Hz, *J*(BX) = 0.2 Hz, Benzothiophen-*5* (B)), 7.35 (2H, ABCXm, *J*(AB) = 7.6 Hz, *J*(AX) = 1.0 Hz, *J*(AC) = 0.6 Hz, Benzothiophen-*4* (A)), 7.3-6.9 (28H, m, DPEphos), 2.56 (6H, s, phen-CH_3). Anal. Calcd (%) for $C_{66}H_{48}CuF_6N_2OP_3S_2$: C, 64.99; H, 3.97; N, 2.30; S, 5.26. Found: C, 64.65; H, 4.33; N, 2.16; S, 4.91.

Cu^I(dmp-2th)(DPEphos)(PF_6) ***(Cu(2th))*** This complex was synthesized according to a literature method.[S3] [$Cu^I(CH_3CN)_4$](PF_6) (25.0 mg, 0.067 mmol) and DPEphos (36.1 mg, 0.067 mmol) were dissolved in CH_2Cl_2 (20 ml). The solution was stirred at r.t. for 0.5 h, and **dmp-2th** (25.0 mg, 0.067 mmol) was added to it. After additional stirring at r.t. for 2 h, the solvent was removed with a rotary evaporator. The product was purified by reprecipitation from a CH_3OH–CH_2Cl_2–*n*-hexane solution.

The resulting yellow powder was filtered off and dried in vacuo. Yield: 40.9 mg (54.5%). ^{1}H NMR (chloroform-*d*): δ (ppm) 8.28 (2H, s, phen-*5*), 7.63 (2H, dd, J($H_{thi5}H_{thi4}$) = 5.3 Hz, J($H_{thi5}H_{thi3}$) = 1.2 Hz, thiophen-*5*), 7.60 (2H, s, phen-*3*), 7.49 (2H, dd, J($H_{thi4}H_{thi3}$) = 3.5 Hz, J($H_{thi5}H_{thi3}$) = 1.2 Hz, thiophen-*3*), 7.31 (2H, dd, J($H_{thi5}H_{thi4}$) = 5.3 Hz, J($H_{thi4}H_{thi3}$) = 3.5 Hz, thiophen-*4*), 7.4-6.9 (28H, m, DPEphos), 2.50 (6H, s, phen-CH_3). Anal. Calcd (%) for $C_{58}H_{44}CuF_6N_2OP_3S_2$: C, 62.22; H, 3.96; N, 2.50; S, 5.73. Found: C, 62.32; H, 4.02; N, 2.31; S, 5.46.

Cu^I(dmp-2Bzth)(DPEphos)(PF_6) ***(Cu(2Bzth))*** This complex was synthesized according to a literature method.[S3] [$Cu^I(CH_3CN)_4$](PF_6) (24.7 mg, 0.066 mmol) and DPEphos (35.5 mg, 0.066 mmol) were dissolved in CH_2Cl_2 (20 ml). The solution was stirred at r.t. for 0.5 h, and **dmp-2Bzth** (31.3 mg, 0.066 mmol) was added to it. After additional stirring at r.t. for 2 h, the solvent was removed with a rotary evaporator. The product was purified by reprecipitation from a CH_3OH–CH_2Cl_2–*n*-hexane solution. The resulting yellow powder was filtered off and dried *in vacuo*. Yield: 68.6 mg (85.2%). ^{1}H NMR (chloroform-*d*): δ (ppm) 8.37 (2H, s, phen-*5*), 8.00 (2H, AAMXX'm, J(XA) = 7.8 Hz, J(XA') = 1.3 Hz, J(XX') = 0.8 Hz, J(MX) = 0.5 Hz, Benzothiophen-*4* (X)), 7.93 (2H, AAMXX'm, J(A'X') = 7.9 Hz, J(AX') = 1.2 Hz, J(XX') = 0.8 Hz, Benzothiophen-*7* (X')), 7.77 (2H, AAMXX'd, J(MX) = 0.5 Hz, Benzothiophen-*3* (M)), 7.71 (2H, s, phen-*3*), 7.49 (2H, AAMXX'm, J(XA) = 7.8 Hz, J(AA') = 7.3 Hz, J(AX') = 1.2 Hz, Benzothiophen-*5* (A)), 7.47 (2H, AAMXX'm, J(A'X') = 7.9 Hz, J(AA') = 7.3 Hz, J(XA') = 1.3 Hz, Benzothiophen-*6* (A')), 7.4-6.9 (28H, m, DPEphos), 2.54 (6H, s, phen-CH_3). Anal. Calcd (%) for $C_{66}H_{48}CuF_6N_2OP_3S_2$: C, 64.99; H, 3.97; N, 2.30; S, 5.26. Found: C, 64.71; H, 4.02; N, 2.27; S, 5.45.

Cu^I(dmp-2Bzfu)(DPEphos)(PF_6) ***(Cu(2Bzfu))*** This complex was synthesized according to a literature method.[S3] [$Cu^I(CH_3CN)_4$](PF_6) (41.4 mg, 0.11 mmol) and DPEphos (59.2 mg, 0.11 mmol) were dissolved in THF (20 ml). The solution was stirred at r.t. for 0.5 h, and **dmp-2Bzfu** (50.0 mg, 0.11 mmol) was added to it. After additional stirring at r.t. for 2 h, the solvent was removed with a rotary evaporator. The product was purified by reprecipitation from a CH_2Cl_2–CH_3OH–*n*-hexane solution. The resulting yellow powder was filtered off and dried *in vacuo*. Yield: 111.2 mg (85.1%). ^1H NMR (chloroform-*d*): δ (ppm) 8.72 (2H, s, phen-*5*), 8.00 (2H, s, phen-*3*), 7.82 (2H, ddd, $J(H_{Bzfur6}H_{Bzfur7})$ = 8.1 Hz, $J(H_{Bzfur5}H_{Bzfur7})$ = 1.0 Hz, $J(H_{Bzfur4}H_{Bzfur7})$ = 0.6 Hz, Benzofuran-*7*), 7.65 (2H, d, $J(H_{Bzfur3}H_{Bzfur4})$ = 0.9 Hz, Benzofuran-*3*), 7.65 (2H, dddd, $J(H_{Bzfur4}H_{Bzfur5})$ = 8.1 Hz, $J(H_{Bzfur4}H_{Bzfur6})$ = $J(H_{Bzfur3}H_{Bzfur4})$ = 0.9 Hz, $J(H_{Bzfur4}H_{Bzfur7})$ = 0.6 Hz, Benzofuran-*4*), 7.46 (2H, ddd, $J(H_{Bzfur4}H_{Bzfur5})$ = 8.1 Hz, $J(H_{Bzfur5}H_{Bzfur6})$ = 7.6 Hz, $J(H_{Bzfur5}H_{Bzfur7})$ = 1.1 Hz, Benzofuran-*5*), 7.37 (2H, ddd, $J(H_{Bzfur6}H_{Bzfur7})$ = 8.1 Hz, $J(H_{Bzfur5}H_{Bzfur6})$ = 7.6 Hz, $J(H_{Bzfur4}H_{Bzfur6})$ = 0.9 Hz, Benzofuran-*6*), 7.4-6.9 (28H, m, DPEphos), 2.55 (6H, s, phen-CH_3). Anal. Calcd (%) for $C_{66}H_{48}CuF_6N_2O_3P_3$: C, 66.75; H, 4.07; N, 2.36. Found: C, 66.57; H, 4.08; N, 2.37.

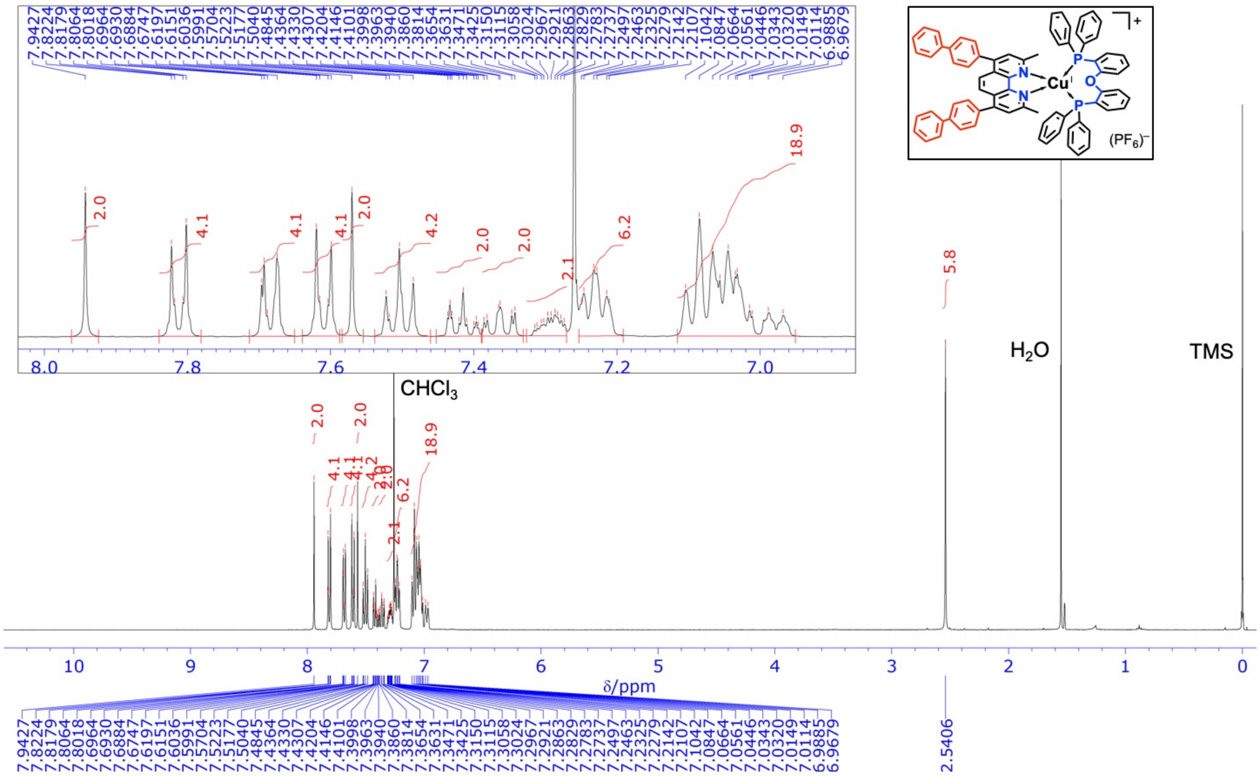

Figure S32. ^{1}H NMR spectrum (400 MHz, chloroform-*d*) of **Cu(Bph)**.

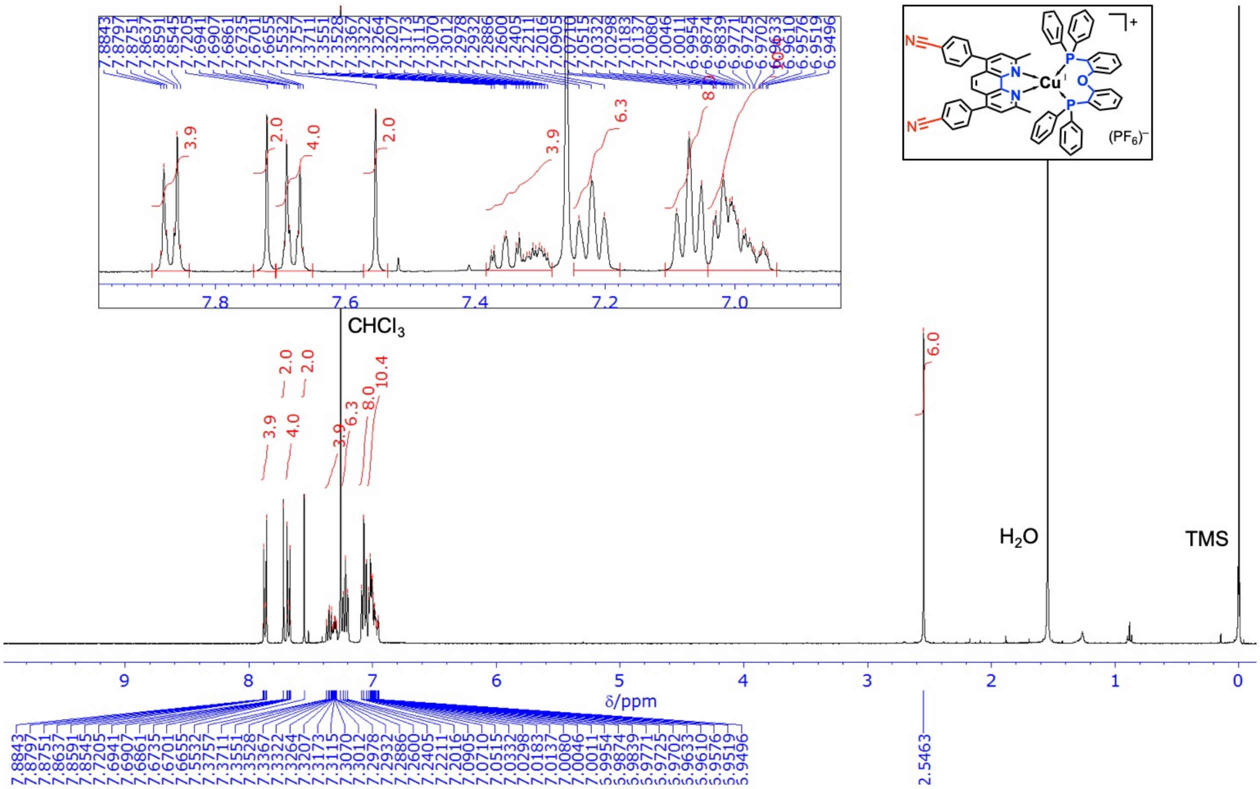

Figure S33. ^{1}H NMR spectrum (400 MHz, chloroform-*d*) of **Cu(NCph)**.

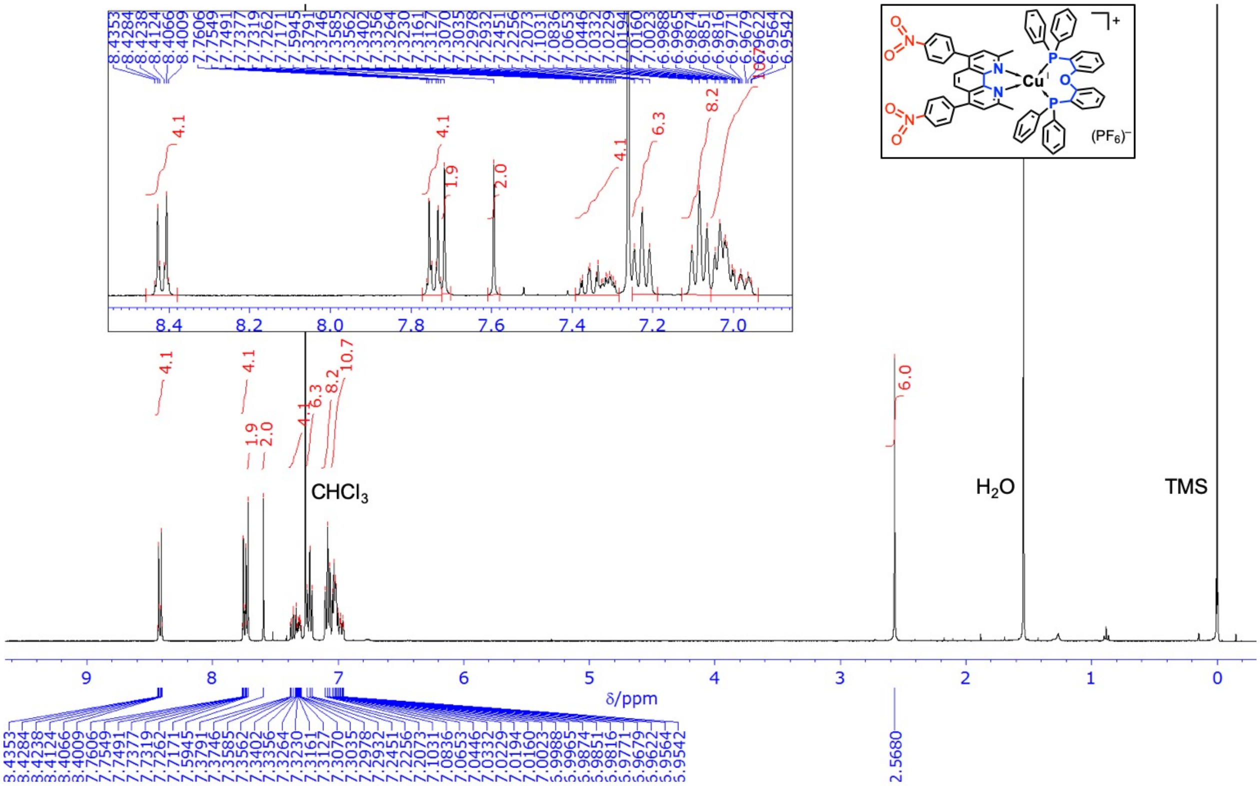

Figure S34. ^{1}H NMR spectrum (400 MHz, chloroform-*d*) of **Cu(NO$_2$ph)**.

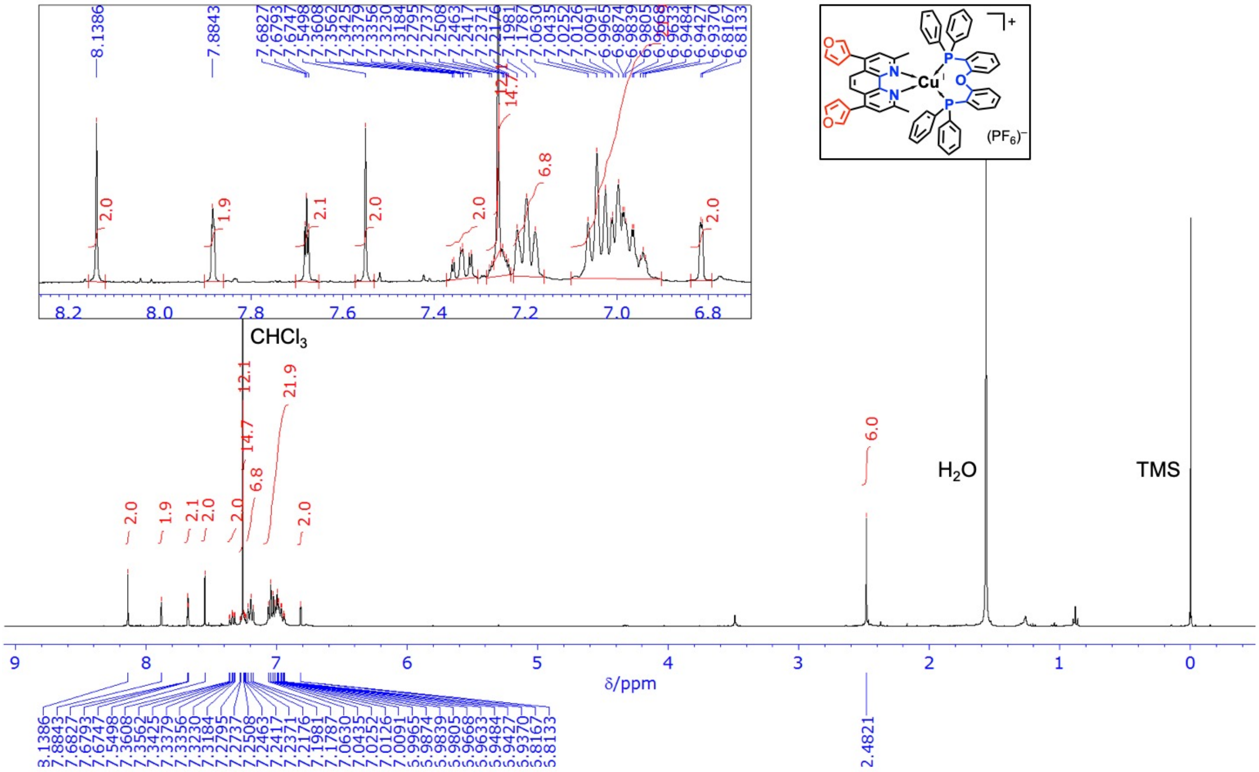

Figure S35. ^{1}H NMR spectrum (400 MHz, chloroform-*d*) of **Cu(3fu)**.

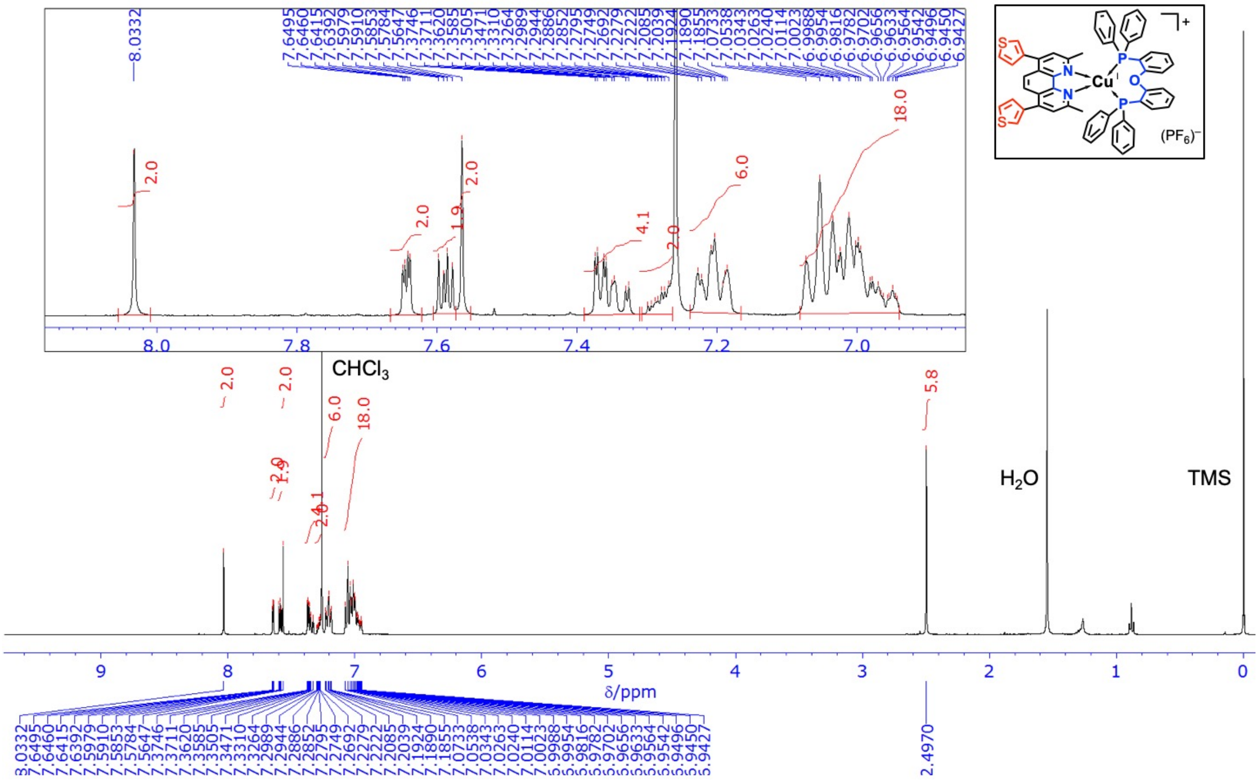

Figure S36. ^{1}H NMR spectrum (400 MHz, chloroform-*d*) of **Cu(3th)**.

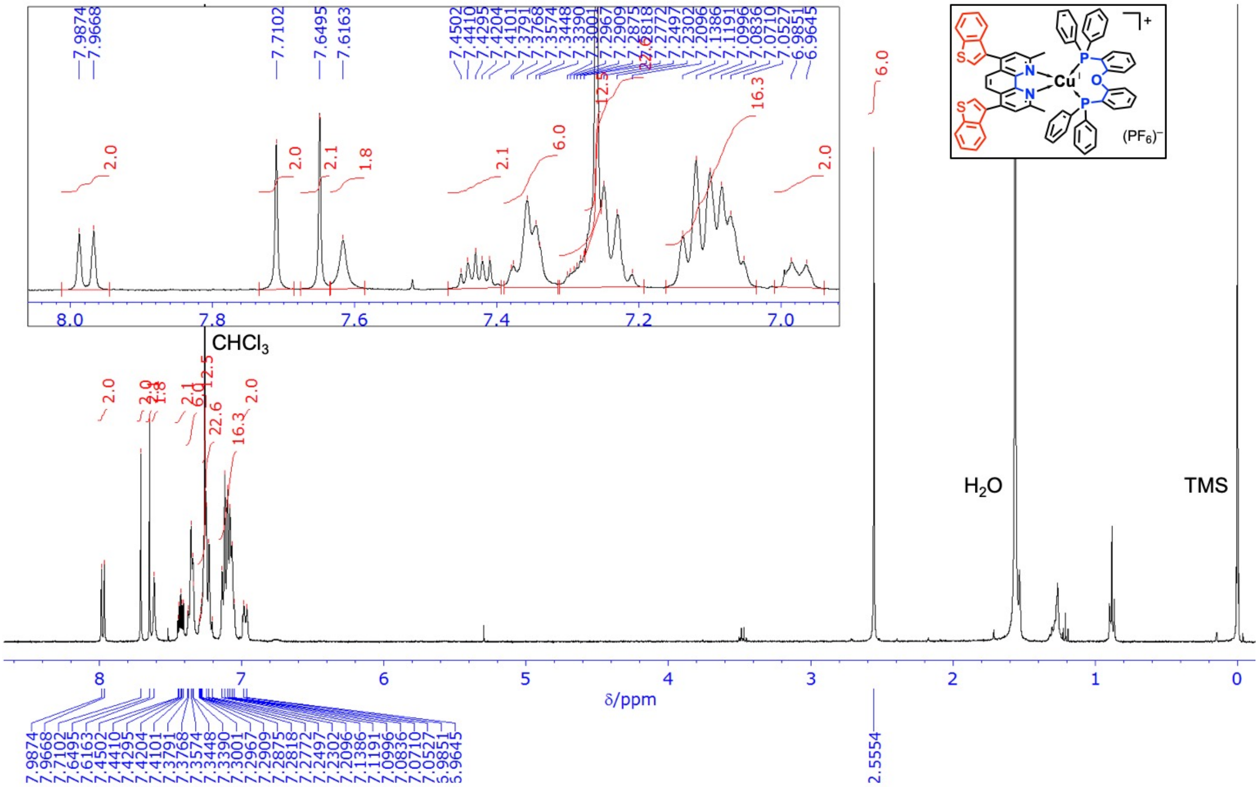

Figure S37. ^{1}H NMR spectrum (400 MHz, chloroform-*d*) of **Cu(3Bzth)**.

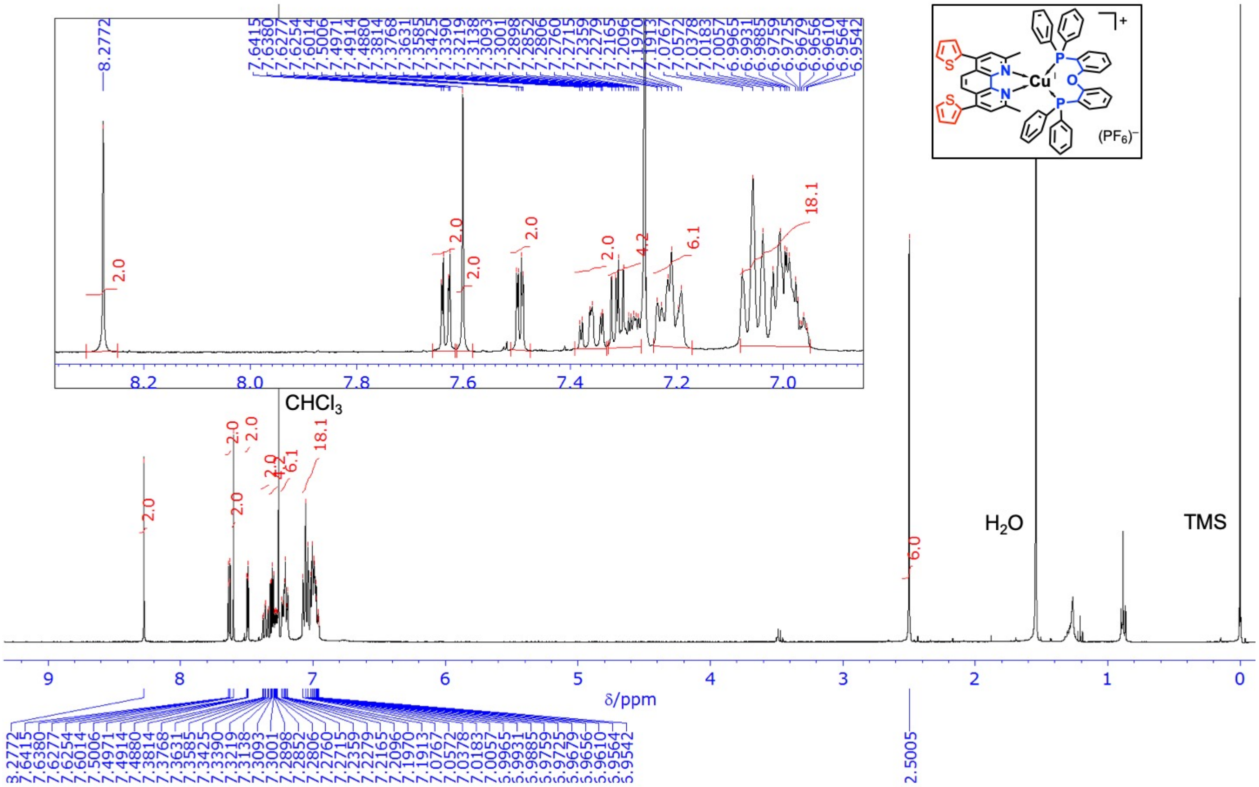

Figure S38. ^{1}H NMR spectrum (400 MHz, chloroform-*d*) of **Cu(2th)**.

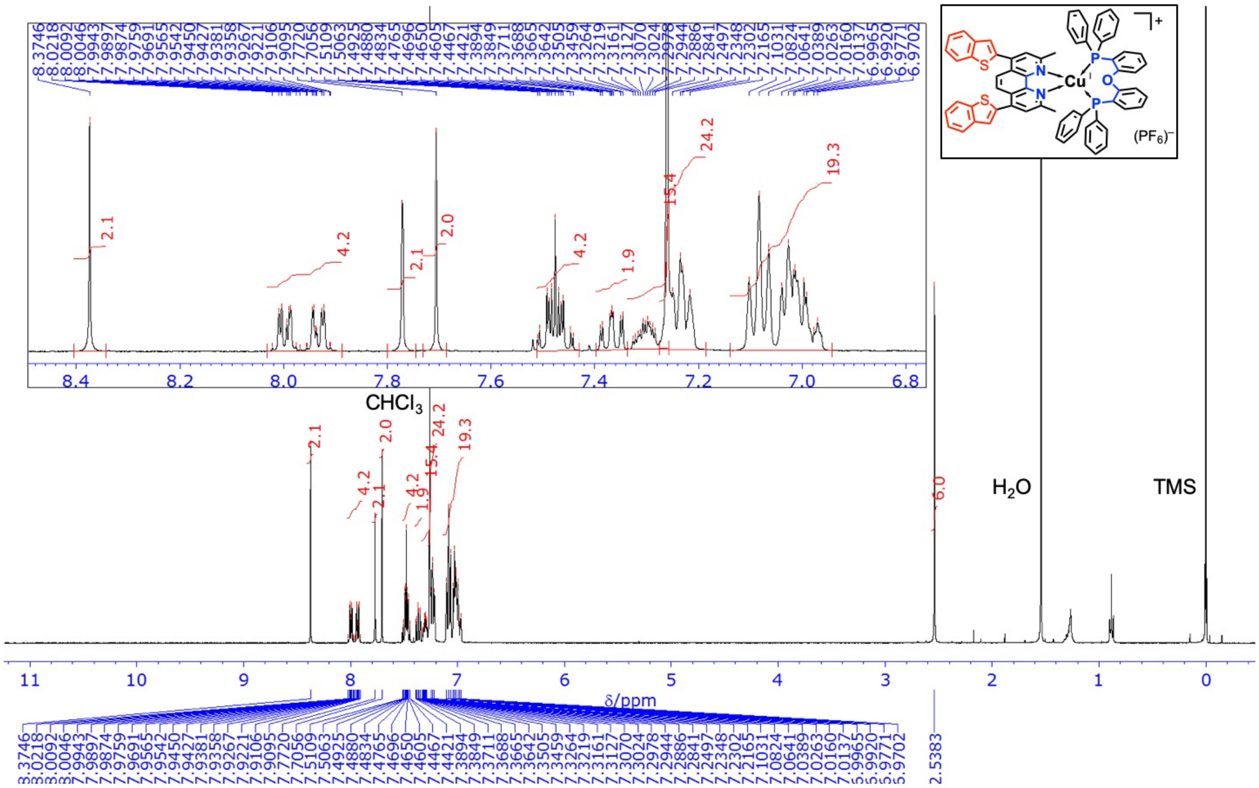

Figure S39. ^{1}H NMR spectrum (400 MHz, chloroform-*d*) of **Cu(2Bzth)**.

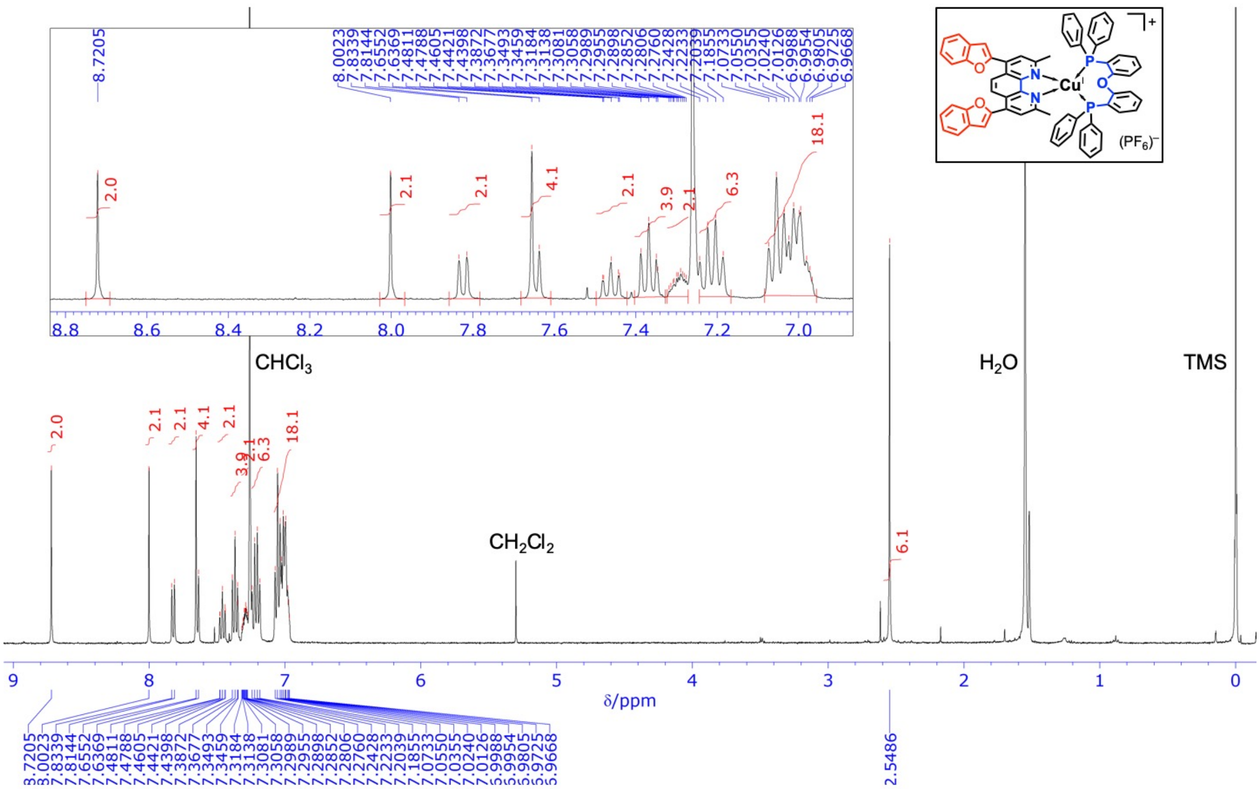

Figure S40. ^{1}H NMR spectrum (400 MHz, chloroform-*d*) of **Cu(2Bzfu)**.

Cu(Bph)

nucleus	n	δ / ppm	multiplicity	*J* / Hz							
H_5	2	7.9429	s								
A1’	2	7.8120	ddd	*J*(A1’X1’)	8.1	*J*(A1A1’)	1.8	*J*(X1A1’)	0.9		
A1	2	7.8109	ddd	*J*(A1X1)	7.1	*J*(A1A1’)	1.8	*J*(A1X1’)	0.9		
A2	2	7.6850	dddd	*J*(A2M2)	7.6	*J*(A2’A2)	1.6	*J*(X2A2)	1.2	*J*(M2'A2)	0.4
A2’	2	7.6830	dddd	*J*(A2’M2')	8.3	*J*(A2’X2)	1.6	*J*(A2’A2)	1.1	*J*(A2’M2)	0.6
X1	2	7.6106	ddd	*J*(A1X1)	7.1	*J*(X1X1’)	1.5	*J*(X1A1’)	0.9		
X1’	2	7.6103	ddd	*J*(A1’X1’)	8.1	*J*(X1X1’)	1.5	*J*(A1X1’)	0.9		
H_3	2	7.5705	s								
M2	2	7.5036	dddd	*J*(A2M2)	7.6	*J*(X2M2)	7.6	*J*(M2'M2)	0.9	*J*(A2’M2)	0.6
M2'	2	7.5034	dddd	*J*(A2’M2')	8.3	*J*(M2'X2)	7.4	*J*(M2'M2)	0.9	*J*(M2'A2)	0.4
X2	2	7.4175	dddd	*J*(X2M2)	7.6	*J*(M2'X2)	7.4	*J*(A2’X2)	1.2	*J*(X2A2)	1.1

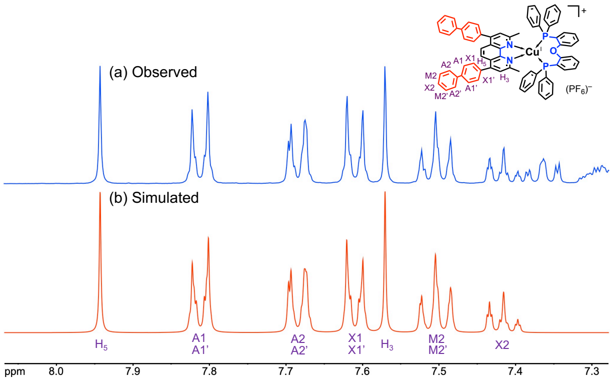

Cu(NCph)

nucleus	n	δ / ppm	multiplicity	*J* / Hz					
A’	2	7.8699	ddd	*J*(A’X’)	7.9	*J*(AA’)	1.8	*J*(XA’)	0.5
A	2	7.8685	ddd	*J*(AX)	7.9	*J*(AA’)	1.8	*J*(AX’)	0.5
H_5	2	7.7213	s						
X	2	7.6815	ddd	*J*(AX)	7.9	*J*(XX’)	1.8	*J*(XA’)	0.5
X’	2	7.6805	ddd	*J*(A’X’)	7.9	*J*(XX’)	1.8	*J*(AX’)	0.5
H_3	2	7.5539	s						

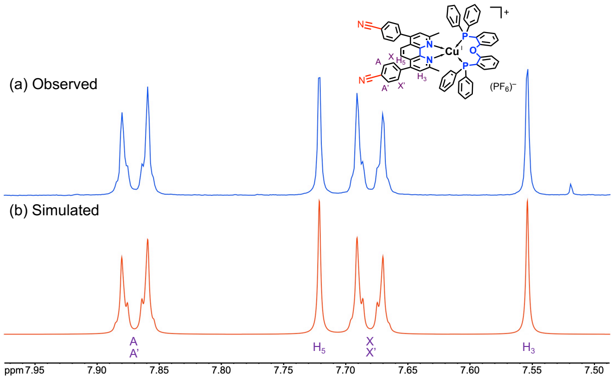

Figure S41. Peak analysis of the ^{1}H NMR spectra (400 MHz, chloroform-*d*) of **Cu(Bph)** and **Cu(NCph)**.

Cu(NO$_2$ph)

nucleus	n	δ / ppm	multiplicity	J / Hz					
A	2	8.4177	ddd	J(AX)	8.4	J(AA')	2.2	J(AX')	0.5
A'	2	8.4165	ddd	J(A'X')	8.4	J(AA')	2.2	J(XA')	0.5
X	2	7.7432	ddd	J(AX)	8.4	J(XX')	2.2	J(XA')	0.5
X'	2	7.7428	ddd	J(A'X')	8.4	J(XX')	2.2	J(AX')	0.5
H_5	2	7.7164	s						
H_3	2	7.5939	s						

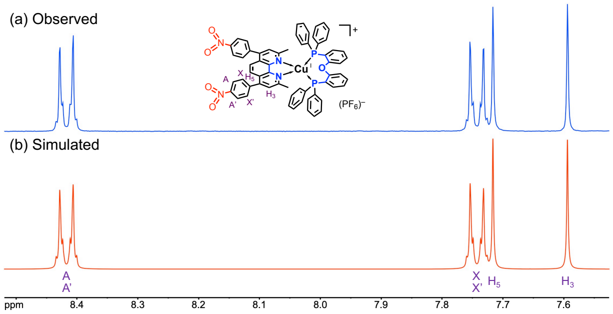

Cu(3fu)

nucleus	n	δ / ppm	multiplicity	J / Hz			
H_5	2	8.1391	s				
H_{fur2}	2	7.8838	dd	$J(H_{fur2}H_{fur5})$	1.5	$J(H_{fur2}H_{fur4})$	0.9
H_{fur5}	2	7.6791	dd	$J(H_{fur5}H_{fur4})$	1.9	$J(H_{fur2}H_{fur5})$	1.5
H_3	2	7.5503	s				
H_{fur4}	2	6.8159	dd	$J(H_{fur5}H_{fur4})$	1.9	$J(H_{fur2}H_{fur4})$	0.9

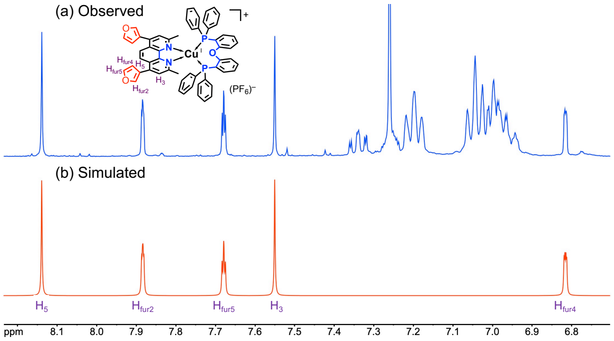

Cu(3th)

nucleus	n	δ / ppm	multiplicity	J / Hz			
H_5	2	8.0328	s				
H_{thi2}	2	7.6440	dd	$J(H_{thi2}H_{thi5})$	2.9	$J(H_{thi2}H_{thi4})$	1.3
H_{thi5}	2	7.5883	dd	$J(H_{thi5}H_{thi4})$	4.8	$J(H_{thi2}H_{thi5})$	2.9
H_3	2	7.5648	s				
H_{thi4}	2	7.3668	dd	$J(H_{thi5}H_{thi4})$	4.8	$J(H_{thi2}H_{thi4})$	1.3

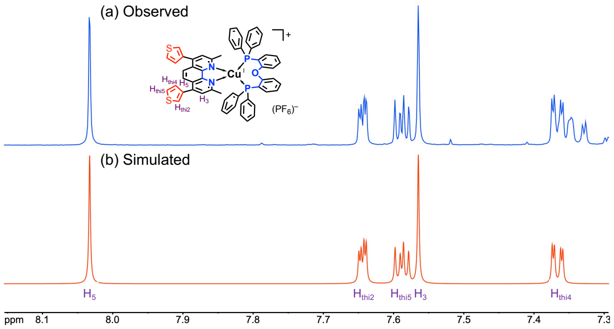

Cu(3Bzth)

nucleus	n	δ / ppm	multiplicity	J / Hz					
X	2	7.9775	ddd	J(CX)	8.2	J(AX)	1.0	J(BX)	0.2
H_5	2	7.7104	s						
H_3	2	7.6492	s						
H_{Bzthi2}	2	7.6167	s						
C	2	7.4290	ddd	J(CX)	8.2	J(BC)	8.0	J(AC)	0.6
B	2	7.3532	ddd	J(BC)	8.0	J(AB)	7.6	J(BX)	0.2
A	2	7.3532	ddd	J(AB)	7.6	J(AX)	1.0	J(AC)	0.6

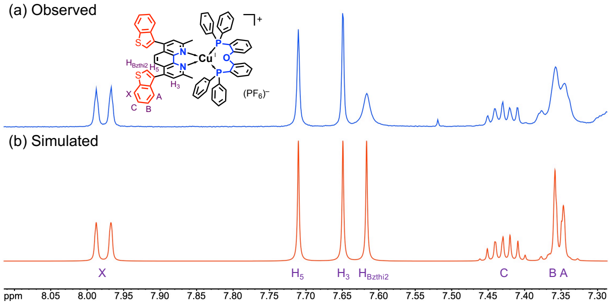

Cu(2th)

nucleus	n	δ / ppm	multiplicity	J / Hz			
H_5	2	8.2764	s				
H_{thi5}	2	7.6330	dd	$J(H_{thi5}H_{thi4})$	5.3	$J(H_{thi5}H_{thi3})$	1.2
H_3	2	7.6013	s				
H_{thi3}	2	7.4942	dd	$J(H_{thi4}H_{thi3})$	3.5	$J(H_{thi5}H_{thi3})$	1.2
H_{thi4}	2	7.3116	dd	$J(H_{thi5}H_{thi4})$	5.3	$J(H_{thi4}H_{thi3})$	3.5

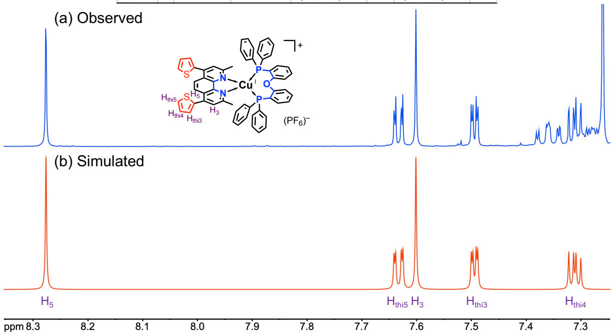

Cu(2Bzth)

nucleus	n	δ / ppm	multiplicity	J / Hz							
H_5	2	8.3741	s								
X	2	7.9974	dddd	J(XA)	7.8	J(XA')	1.3	J(XX')	0.8	J(MX)	0.5
X'	2	7.9327	ddd	J(A'X')	7.9	J(AX')	1.2	J(XX')	0.8		
M	2	7.7707	d	J(MX)	0.5						
H_3	2	7.7056	s								
A	2	7.4868	ddd	J(XA)	7.8	J(AA')	7.3	J(AX')	1.2		
A'	2	7.4660	ddd	J(A'X')	7.9	J(AA')	7.3	J(XA')	1.3		

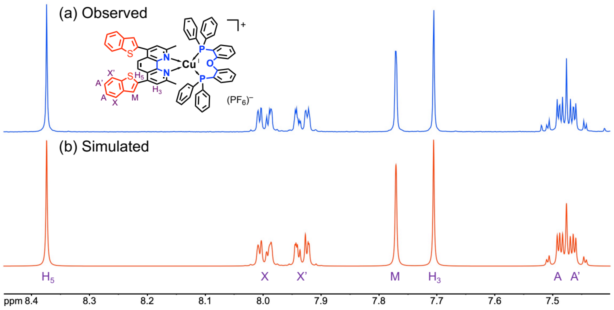

Figure S42. Peak analysis of the ^{1}H NMR spectra (400 MHz, chloroform-*d*) of **Cu(NO$_2$ph)**, **Cu(3fu)**, **Cu(3th)**, **Cu(3Bzth)**, **Cu(2th),** and **Cu(2Bzth)**.

Cu(2Bzfu)

nucleus	n	δ / ppm	multiplicity	J / Hz							
H_5	2	8.7208	s								
H_3	2	8.0017	s								
H_{Bzfur7}	2	7.8227	ddd	$J(H_{Bzfur6}H_{Bzfur7})$	8.1	$J(H_{Bzfur5}H_{Bzfur7})$	1.0	$J(H_{Bzfur4}H_{Bzfur7})$	0.6		
H_{Bzfur3}	2	7.6541	d	$J(H_{Bzfur3}H_{Bzfur4})$	0.9						
H_{Bzfur4}	2	7.6468	dddd	$J(H_{Bzfur4}H_{Bzfur5})$	8.1	$J(H_{Bzfur4}H_{Bzfur6})$	0.9	$J(H_{Bzfur3}H_{Bzfur4})$	0.9	$J(H_{Bzfur4}H_{Bzfur7})$	0.6
H_{Bzfur5}	2	7.4605	ddd	$J(H_{Bzfur4}H_{Bzfur5})$	8.1	$J(H_{Bzfur5}H_{Bzfur6})$	7.6	$J(H_{Bzfur5}H_{Bzfur7})$	1.1		
H_{Bzfur6}	2	7.3682	ddd	$J(H_{Bzfur6}H_{Bzfur7})$	8.1	$J(H_{Bzfur5}H_{Bzfur6})$	7.6	$J(H_{Bzfur4}H_{Bzfur6})$	0.9		

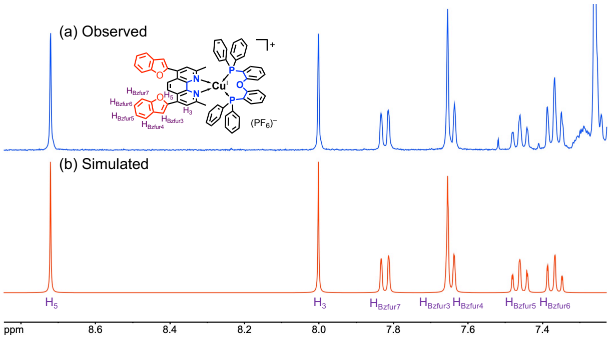

Figure S43. Peak analysis of the ^{1}H NMR spectra (400 MHz, chloroform-*d*) of **Cu(2Bzfu)**.

Crystal Structure Determination.

Suitable crystals for X-ray diffraction analysis were obtained for **Cu(ph)** as **Cu(ph)**·1.85(Et_2O)·0.3(MeOH) (yellow crystals, 0.4 × 0.7 × 0.3 mm^3) by slow diffusion of Et_2O vapor into a hot solution of MeOH at 4ºC, for **Cu(NCph)** as **Cu(NCph)**•Et_2O (yellow platelet crystals, 0.27 × 0.12 × 0.02 mm^3) by slow diffusion of Et_2O vapor into a solution of MeOH from –20 ºC to r.t., for **Cu(2Bzth)** (yellow platelet crystals, 0.11 × 0.08 × 0.04 mm^3) by slow diffusion of Et_2O vapor into a solution of MeOH from –20º C to r.t., and for **Cu(2Bzfu)** (yellow platelet crystals, 0.22 × 0.07 × 0.02 mm^3) by slow diffusion of Et_2O vapor a solution of MeOH at r.t.

Diffraction data were collected on a Rigaku R-AXIS RAPID II (SPIDER) imaging-plate diffractometer equipped with a Rigaku VariMax confocal optical system for Cu-Kα radiation (λ = 1.54184 Å) at 93 or 121 K using a Rigaku low-temperature apparatus. The initial structure were solved by direct methods, using the SIR2014[S11] or a charge flipping method, using the SUPERFLIP[S12]/EDMA[S13] programs, and was refined on F^2 by means of full-matrix least-squares procedures, using the SHELXL-2014/6 or SHELXL-2018/1 programs.[S14] Tables S2-S6 provide crystallographic data details.

In the least-squares refinements, all non-hydrogen atoms were refined using anisotropic displacement parameters. For the methyl and hydroxyl groups, the H atoms were generated in the calculated positions with torsion angles from the electron densities around the central C or O atoms, respectively, using a riding-model with isotropic thermal parameters 1.5 times those of the attached C or O atoms, respectively. For methylene group and aromatic C–H bonds, the H atoms were geometrically generated and refined as a riding-model with isotropic thermal parameters 1.2 times those of the attached C atoms.

The following treatments were used for the disordered parts in the crystal structures:

Cu(ph) The structures of the disordered PF_6 anions were refined by dividing one PF_6 moiety into two parts with restraints. The "DFIX" command was applied to the P–F distances, which were fixed at 1.579 Å. For neighboring F–F distances, which were fixed at 2.233 Å, the "SIMU" command was used for each F atom in the different parts of the PF_6 anions to preserve their octahedral structures and unify F atoms' thermal ellipsoids. The three types of Et_2O molecules present in the unit cell were refined with restraints. The "DFIX" command was used to fix the C–O distances at 1.43 Å, neighboring C–C bond distances at 1.52 Å, the 1,3 distances of the C–C–O bonds at 2.41 Å, and the 1,3 distances of the C–O–C bonds at 2.27 Å to preserve a suitable structure for Et_2O. The MeOH molecules were refined with restraints. "DFIX" was used to fix each C–O distance at 1.43 Å to preserve a suitable structure for MeOH. The structures of two types of disordered solvent molecules on inversion centers were refined as single 0.5 occupied Et_2O molecules, using no restraints for the thermal factors or by dividing them into two parts and summing the occupation as 0.5 each of one Et_2O molecule or two MeOH molecules. The thermal factors of the latter case were restrained using the "RIGU" command for all five atoms in the Et_2O part and "SIMU" for the same types of atoms in the two

parts of the MeOH molecules. The other disordered Et_2O molecule was refined by dividing it into two parts using the "SIMU" restraint for the same type of atoms in the different parts.

Cu(2Bzth) One PF_6 anion was refined by dividing it into two parts with restraints. The "DFIX" command was used to fix P–F distances at 1.579 Å and neighboring F–F distances at 2.233 Å, and the "SIMU" command was used for the same F and P atoms in the different parts of the PF_6 anion to preserve its octahedral structure and unify the F atoms' thermal ellipsoids. One DPEphos ligand was refined by dividing into two parts: Two P atoms and two phenyl rings were shared, with restraints, using the "SADI" command for C–O bonds and P–C bonds in the different parts. The "AFIX66" command fixed the structures of the phenyl groups as aromatic 6-membered rings, and the "EADP" command constrained the same types of C atoms and O atoms in the different parts to unify these atoms' thermal ellipsoids. The occupancy of the minor DPEphos part was 0.16, which made it difficult to refine the crystal structure without these restraints and constraints.

Cu(Bzfu) One DPEphos ligand was refined by dividing it into two parts. One phenyl ring was shared, with restraints, using the "SADI" command for the same types of Cu–P, Cu–phenyl, P–phenyl, C–O, and the 1,3-distance of the C–O–C bonds in the different parts. The "AFIX66" command fixed the structure of the phenyl groups as aromatic 6-membered rings, and the "FLAT" command fixed the planarity of the partial structure of the P–phenyl and P–phenyl–O in a minor part of the structure. The "EADP" command constrained the same C and O atoms in the different parts to unify these atoms' thermal ellipsoids. The occupancy of the minor DPEphos part was 0.07, which made it difficult to refine the crystal structure without these restraints and constraints.

Crystallographic data for the structures has been deposited at the Cambridge Crystallographic Data Center (CCDC numbers: 1895526-1895529).

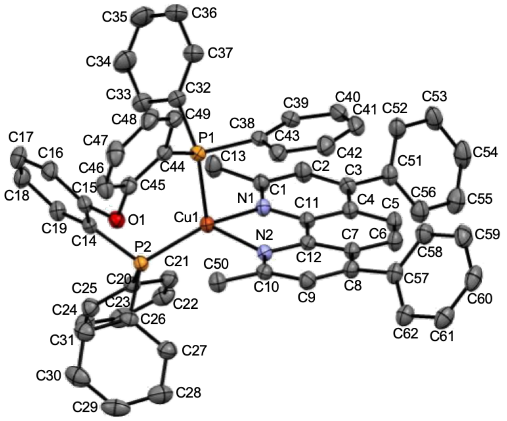

Figure S44. ORTEP model of the crystal structure of **Cu(ph)**·1.85(Et_2O)·0.3(MeOH). H atoms, PF_6^- anions, and solvent molecules are omitted for clarity. Displacement ellipsoids are drawn at the 50% probability level.

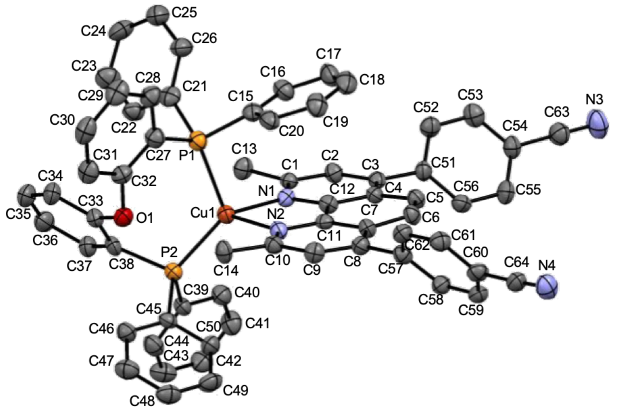

Figure S45. ORTEP model of the crystal structure of **Cu(NCph)**•Et_2O. H atoms, PF_6^- anions, and solvent molecules are omitted for clarity. Displacement ellipsoids are drawn at the 50% probability level.

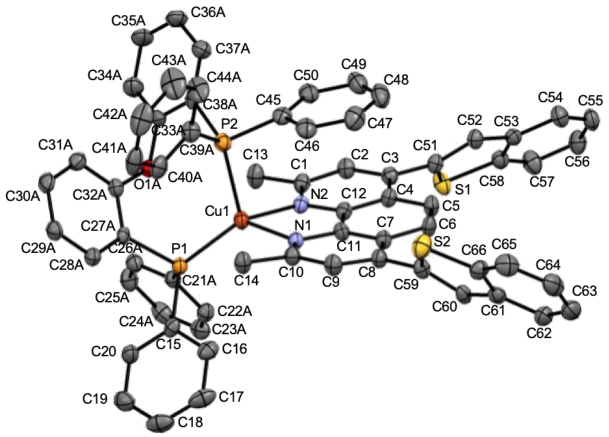

Figure S46. ORTEP model of the crystal structure of **Cu(2Bzth)**. H antoms, PF_6^- anions, and the other part of DPEphos are omitted for clarity. Displacement ellipsoids are drawn at the 50% probability level.

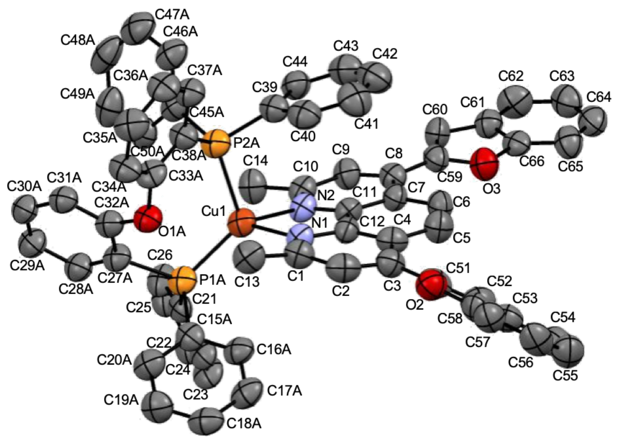

Figure S47. ORTEP model of the crystal structure of **Cu(2Bzfu)**. H atoms, PF_6^- anions, and the other part of DPEphos are omitted for clarity. Displacement ellipsoids are drawn at the 50% probability level.

Table S2. Crystallographic Data for the Cu Complexes

	**Cu(ph)**·1.85(Et_2O)·0.3(MeOH)	**Cu(NCph)**·Et_2O	**Cu(2Bzth)**	**Cu(2Bzfu)**
Empirical formula	$C_{69.7}H_{67.7}CuF_6N_2O_{3.15}P_3$	$C_{68}H_{56}CuF_6N_4O_2P_3$	$C_{66}H_{48}CuF_6N_2OP_3S_2$	$C_{66}H_{48}CuF_6N_2O_3P_3$
Formula weight	1254.2	1231.6	1219.6	1187.5
Crystal system	Triclinic	Monoclinic	Monoclinic	Monoclinic
Space group	$P-1$	Cc	$P2_1/n$	$P2_1/c$
a / Å	13.0866(2)	25.2690(5)	12.8297(2)	16.5427(9)
b / Å	13.8935(3)	10.3808(2)	31.7439(6)	19.4269(10)
c / Å	17.7742(3)	23.3271(4)	13.4204(3)	17.0695(10)
α / deg	91.467(1)	90	90	90
β / deg	106.610(1)	105.514(1)	90.793(1)	94.292(2)
γ / deg	92.064(1)	90	90	90
V / Å^3	3092.65(10)	5896.04(19)	5465.13(18)	5470.3(5)
Z	2	4	4	4
T / K	121	93	93	93
R_{int}	0.0590	0.0545	0.0455	0.1311
Number of total reflections	11116	10394	9920	10005
Number of parameters	973	761	776	712
$R1$ [$I > 2\sigma(I)$]^a	0.0478	0.0530	0.0455	0.0794
$wR2$ [$I > 2\sigma(I)$]^b	0.1268	0.1286	0.1189	0.2031
GOF^c on F^2	1.100	1.111	1.043	1.016

$^aR1 = \Sigma(||F_O| - |F_c||) / \Sigma|F_O|$. $^bwR2 = [\Sigma[w(F_o^2 - F_c^2)^2] / \Sigma[w(F_o^2)^2]]^{1/2}$. ^{c}GOF $= [\Sigma w(|F_o^2| - |F_c^2|)^2 / (m - n)]^{1/2}$, where m = the number of reflections and n = the number of parameters.

Table S3. Selected Bond Lengths (Å) and Angles (deg) for **Cu(ph)**·1.85(Et_2O)·0.3(MeOH)

Bond Lengths					
Cu(1)–P(1)	2.3104(6)	Cu(1)–P(2)	2.2220(5)	Cu(1)–N(1)	2.0747(17)
Cu(1)–N(2)	2.0906(16)	P(1)–C(32)	1.830(2)	P(1)–C(38)	1.835(2)
P(1)–C(44)	1.838(2)	P(2)–C(14)	1.827(2)	P(2)–C(20)	1.833(2)
P(2)–C(26)	1.826(2)	C(3)–C(51)	1.487(3)	C(8)–C(57)	1.488(3)
Bond Angles					
P(1)–Cu(1)–P(2)	116.79(2)	P(1)–Cu(1)–N(1)	101.69(5)	P(1)–Cu(1)–N(2)	99.18(5)
P(2)–Cu(1)–N(1)	126.00(5)	P(2)–Cu(1)–N(2)	124.81(5)	N(1)–Cu(1)–N(2)	80.57(7)
Cu(1)–P(1)–C(32)	122.08(8)	Cu(1)–P(1)–C(38)	103.20(7)	Cu(1)–P(1)–C(44)	119.85(7)
C(32)–P(1)–C(38)	103.56(10)	C(32)–P(1)–C(44)	102.15(10)	C(38)–P(1)–C(44)	103.46(10)
Cu(1)–P(2)–C(14)	109.48(6)	Cu(1)–P(2)–C(20)	119.89(7)	Cu(1)–P(2)–C(26)	116.92(7)
C(14)–P(2)–C(20)	103.78(9)	C(14)–P(2)–C(26)	105.41(9)	C(20)–P(2)–C(26)	99.69(9)

Table S4. Selected Bond Lengths (Å) and Angles (deg) for **Cu(NCph)**·Et_2O

Bond Lengths					
Cu(1)–P(1)	2.280(2)	Cu(1)–P(2)	2.2213(18)	Cu(1)–N(1)	2.052(5)
Cu(1)–N(2)	2.082(5)	P(1)–C(15)	1.825(7)	P(1)–C(21)	1.827(7)
P(1)–C(27)	1.833(7)	P(2)–C(38)	1.832(7)	P(2)–C(39)	1.810(7)
P(2)–C(45)	1.828(6)	C(3)–C(51)	1.482(8)	C(8)–C(57)	1.496(9)
Bond Angles					
P(1)–Cu(1)–P(2)	118.91(7)	P(1)–Cu(1)–N(1)	103.93(15)	P(1)–Cu(1)–N(2)	103.12(15)
P(2)–Cu(1)–N(1)	124.37(14)	P(2)–Cu(1)–N(2)	117.87(15)	N(1)–Cu(1)–N(2)	81.30(19)
Cu(1)–P(1)–C(15)	101.5(2)	Cu(1)–P(1)–C(21)	124.8(2)	Cu(1)–P(1)–C(27)	119.3(2)
C(15)–P(1)–C(21)	104.9(3)	C(15)–P(1)–C(27)	105.9(3)	C(21)–P(1)–C(27)	98.6(3)
Cu(1)–P(2)–C(38)	109.8(2)	Cu(1)–P(2)–C(39)	119.0(2)	Cu(1)–P(2)–C(45)	115.4(2)
C(38)–P(2)–C(39)	105.2(3)	C(38)–P(2)–C(45)	106.0(3)	C(39)–P(2)–C(45)	100.2(3)

Table S5. Selected Bond Lengths (Å) and Angles (deg) for **Cu(2Bzth)**

Bond Lengths					
Cu(1)–P(1)	2.2228(8)	Cu(1)–P(2)	2.3195(8)	Cu(1)–N(1)	2.065(2)
Cu(1)–N(2)	2.083(2)	P(1)–C(15)	1.824(3)	P(1)–C(21A)	1.8225(18)
P(1)–C(27A)	1.8530(15)	P(2)–C(38A)	1.853(3)	P(2)–C(39A)	1.877(3)
P(2)–C(45)	1.821(3)	C(3)–C(51)	1.478(3)	C(8)–C(59)	1.479(4)
Bond Angles					
P(1)–Cu(1)–P(2)	116.66(3)	P(1)–Cu(1)–N(1)	125.89(6)	P(1)–Cu(1)–N(2)	123.93(6)
P(2)–Cu(1)–N(1)	102.36(6)	P(2)–Cu(1)–N(2)	99.67(6)	N(1)–Cu(1)–N(2)	80.81(9)
Cu(1)–P(1)–C(15)	121.36(9)	Cu(1)–P(1)–C(21A)	118.70(8)	Cu(1)–P(1)–C(27A)	107.03(7)
C(15)–P(1)–C(21A)	97.88(12)	C(15)–P(1)–C(27A)	102.72(11)	C(21A)–P(1)–C(27A)	107.48(10)
Cu(1)–P(2)–C(38A)	119.66(16)	Cu(1)–P(2)–C(39A)	121.93(19)	Cu(1)–P(2)–C(45)	102.70(9)
C(38A)–P(2)–C(39A)	102.7(3)	C(38A)–P(2)–C(45)	104.57(17)	C(39A)–P(2)–C(45)	102.8(2)

Table S6. Selected Bond Lengths (Å) and Angles (deg) for **Cu(2Bzfu)**

Bond Lengths					
Cu(1)–P(1A)	2.2278(19)	Cu(1)–P(2A)	2.276(2)	Cu(1)–N(1)	2.081(4)
Cu(1)–N(2)	2.061(4)	P(1A)–C(15A)	1.828(3)	P(1A)–C(21A)	1.856(3)
P(1A)–C(27A)	1.860(3)	P(2A)–C(38A)	1.846(3)	P(2A)–C(39)	1.819(6)
P(2A)–C(45A)	1.833(4)	C(3)–C(51)	1.467(7)	C(8)–C(59)	1.462(6)
Bond Angles					
P(1A)–Cu(1)–P(2A)	117.42(9)	P(1A)–Cu(1)–N(1)	119.29(15)	P(1A)–Cu(1)–N(2)	126.70(12)
P(2A)–Cu(1)–N(1)	104.47(16)	P(2A)–Cu(1)–N(2)	101.80(15)	N(1)–Cu(1)–N(2)	79.99(16)
C(15A)–P(1A)–C(21A)	102.2(2)	C(15A)–P(1A)–C(27A)	106.2(2)	C(21A)–P(1A)–C(27A)	104.0(2)
Cu(1)–P(2A)–C(38A)	117.15(18)	Cu(1)–P(2A)–C(39)	103.75(18)	Cu(1)–P(2A)–C(45A)	121.7(2)
C(38A)–P(2A)–C(39)	104.7(4)	C(38A)–P(2A)–C(45A)	101.1(2)	C(39)–P(2A)–C(45A)	107.1(4)

References

S1. Larsen, A. F., and Ulven, T. (2011). Efficient Synthesis of 4,7-Diamino Substituted 1,10-Phenanthroline-2,9-dicarboxamides. *Org. Lett.* 13, 3546-3548. doi: 10.1021/ol201321z

S2. Kubas, G. (1990). Tetrakis(Acetonitrile)Copper(1+) Hexafluorophosphate(1-). *Inorg. Synth.* 28, 68–70. doi: 10.1002/9780470132593.ch15

S3. Takeda, H., Ohashi, K., Sekine, A., and Ishitani, O. (2016). Photocatalytic CO_2 Reduction Using Cu(I) Photosensitizers with a Fe(II) Catalyst. *J. Am. Chem. Soc.* 138, 4354–4357. doi: 10.1021/jacs.6b01970

S4. Cuttell, D. G., Kuang, S.-M., Fanwick, P. E., McMillin, D. R., and Walton, R. A. (2002). Simple Cu(I) Complexes with Unprecedented Excited-State Lifetimes. *J. Am. Chem. Soc.* 124, 6–7. doi: 10.1021/ja012247h

S5. Luo, S., Mejía, E., Friedrich, A., Pazidis, A., Junge, H., Surkus, A.-E., Jackstell, R., Denurra, S., Gladiali, S., Lochbrunner, S., and Beller, M. (2013). Photocatalytic water reduction with copper-based photosensitizers: A noble-metal-free system. *Angew. Chem. Int. Ed.* 52, 419–423. doi: 10.1002/anie.201205915

S6. König, E., Ritter, G., Madeja, K., Kobetić, R., Gembarovski, D., Baranović, G., and Gabelica, V. (1981). Metal Complexes of 2,9-Dimethyl-1,10-Phenanthroline and Derivatives—I. Iron(II) Complexes. *J. Inorg. Nucl. Chem.* 43, 2273–2280. doi: 10.1016/0022-1902(81)80248-5

S7. Elliott, C. M., and Hershenhart, E. (1982). Electrochemical and spectral investigations of ring-substituted bipyridine complexes of ruthenium. *J. Am. Chem. Soc.* 104, 7519–7526. doi: 10.1021/ja00390a022

S8. Takeda, H., Koizumi, H., Okamoto, K., and Ishitani, O. (2014). Photocatalytic CO_2 reduction using a Mn complex as a catalyst. *Chem. Commun.* 50, 1491–1493. doi: 10.1039/C3CC48122K

S9. (a) Tamaki, Y., Koike, K., Morimoto, T., and Ishitani, O. (2013). Substantial improvement in the efficiency and durability of a photocatalyst for carbon dioxide reduction using a benzoimidazole derivative as an electron donor. *J. Catal.* 304, 22–28. doi: 10.1016/j.jcat.2013.04.002, (b) Hasegawa, E., Seida, T., Chiba, N., Takahashi, T., and Ikeda, H. (2005). Contrastive photoreduction pathways of benzophenones governed by regiospecific deprotonation of imidazoline radical cations and additive effects. *J. Org. Chem.* 70, 9632–9635. doi: 10.1021/jo0514220, (c) Zhu, X.-Q., Zhang, M.-T., Yu, A., Wang, C.-H., and Cheng, J.-P. (2008). Hydride, hydrogen atom, proton, and electron transfer driving forces of various five-membered heterocyclic organic hydrides and their reaction intermediates in acetonitrile. *J. Am. Chem. Soc.* 130, 2501–2516. doi: 10.1021/ja075523m

S10. Yang, J., Liu, S., Zheng, J.-F., and Zhou, J. (S.) (2012). Room-Temperature Suzuki–Miyaura Coupling of Heteroaryl Chlorides and Tosylates. *Eur. J. Org. Chem.*, 6248-6259. doi: 10.1002/ejoc.201200918

S11. Burla, M. C., Caliandro, R., Carrozzini, B., Cascarano, G. L., Cuocci, C., Giacovazzo, C., Mallamo, M., Mazzone, A., and Polidori, G. (2015). Crystal structure determination and refinement via SIR2014. *J. Appl. Crystallogr.* 48, 306–309. doi: 10.1107/S1600576715001132

S12. Palatinus, L., and Chapuis, G. (2007). SUPERFLIP-A computer program for the solution of crystal structures by charge flipping in arbitrary dimensions. *J. Appl. Crystallogr.* 40, 786–790. doi: 10.1107/S0021889807029238

S13. Palatinus, L., Prathapa, S. J., and van Smaalen, S. (2012). EDMA: A computer program for topological analysis of discrete electron densities. *J. Appl. Crystallogr.* 45, 575–580. doi: 10.1107/S0021889812016068

S14. Sheldrick, G. M. (2008). A short history of SHELX. *Acta Cryst.* A64, 112–122. doi: 10.1107/S0108767307043930
